# Coordination-Induced Spin Modulation: Overcoming Spin Blocking in C–H Methylation with High-Spin Ferrous Complexes

**DOI:** 10.1021/acscatal.6c01157

**Published:** 2026-03-04

**Authors:** Tianyi Zhang, Matthew V. Pecoraro, Paul J. Chirik

**Affiliations:** † Department of Chemistry, 6740 Princeton University, Princeton, New Jersey 08544, United States

**Keywords:** Earth-Abundant Metals, C−H Functionalization, Reactivity Control, Spin State, Mechanism

## Abstract

Understanding spin state changes and their influence on the reactivity of earth-abundant transition metal complexes is essential for unlocking the full potential of these elements. While precedented in biological contexts and related model complexes, control of metal spin state is underexplored as a mode for reactivity control in organometallic chemistry. Here we describe coordination-induced spin modulation as a strategy to overcome spin blocking to enable C–H functionalization with four-coordinate high-spin iron­(II) dimethyl complexes. The addition of monodentate phosphines induced room-temperature C–H methylation of arenes, while the evaluation of sterics and σ-donacity demonstrated PhPMe_2_ to be an optimal spin modulator (L). Mechanistic experiments, kinetics, a stereochemical probe, and computations corroborated the spin-state lowering upon the coordination of L, enabling the previously spin-blocked substrate association to form a five-coordinate (bisphosphine)­(L)­Fe­(CH_3_)_2_ intermediate directly observed by NMR spectroscopy. The lability of L allowed ligand dissociation and the formation of the σ-agostic complex en route to C–H functionalization.

## Graphical abstract

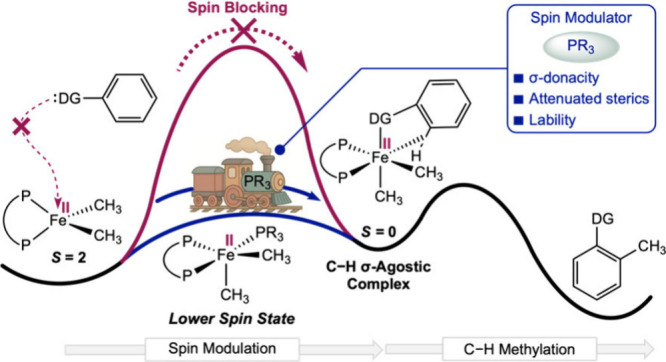

## Introduction

Spin state changes constitute an intrinsic component of the reactivity of transition metal complexes, especially with earth-abundant first-row metals, as their higher density of states contributes to an increased tendency for spin crossover.^1^ Known studies on spin state changes have been largely constrained to the C–H oxidation reactivity of cytochrome P450 enzymes, typically proceeding through an outer-sphere radical pathway.^2–4^ For example, Shaik and co-workers proposed the two-state reactivity (TSR) model, where the crossover between high- and low-spin states of an iron center lowers the activation barrier for heme-mediated C–H oxidation (Figure 1A). Key principles of the TSR model have since been computationally established^5–7^ and with synthetic and spectroscopic studies on related compounds.^8–13^ Likewise, spin state switching due to reversible ligand coordination has been observed in porphyrin metal complexes^14–16^ and in solid-state materials.^17,18^

**Figure 1:**
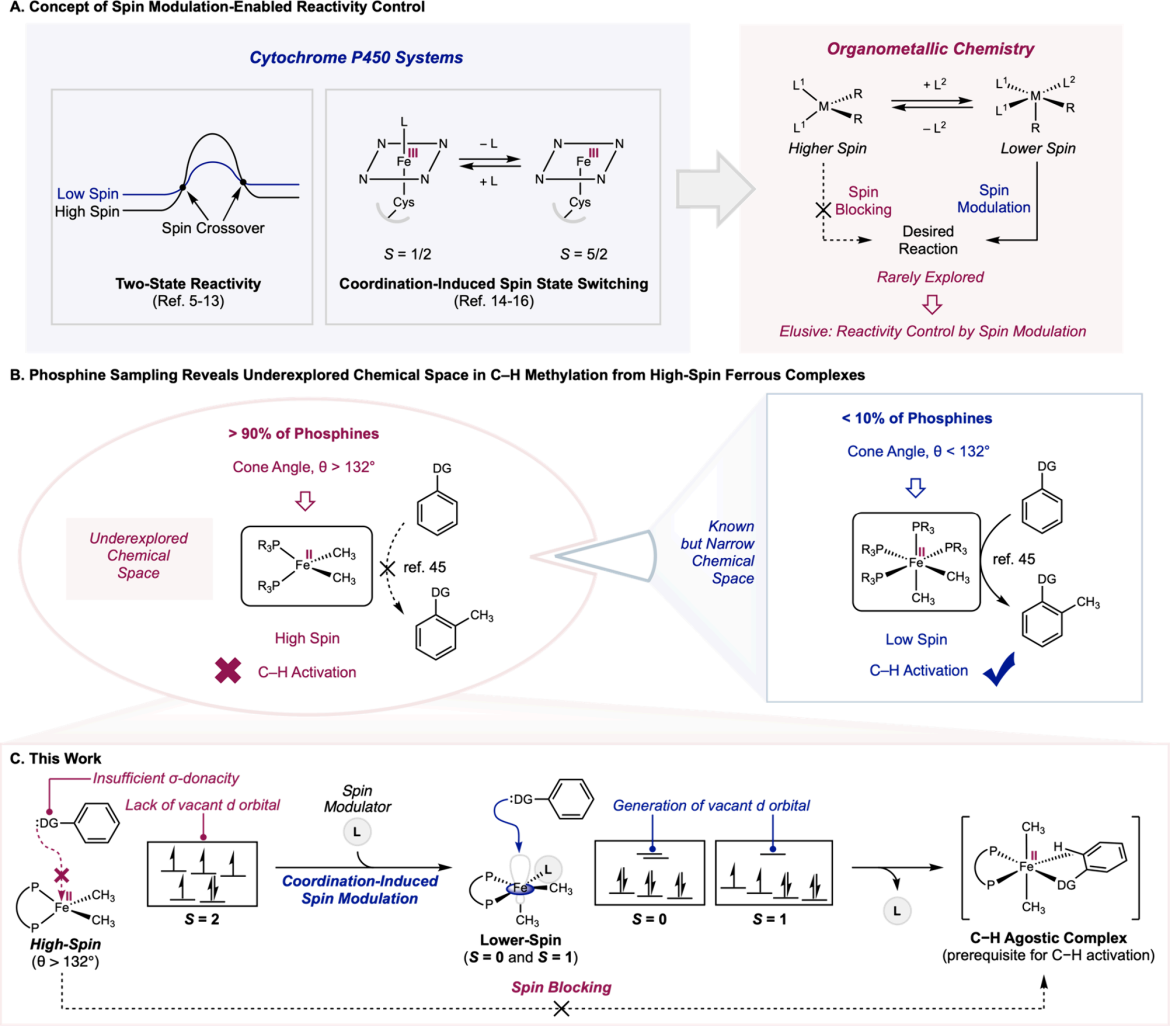
Proposed strategy of spin modulation for promoting directed arene C–H methylation starting from a high-spin iron­(II) dimethyl complex. (A) Concept of spin modulation-enabled reactivity control. (B) Phosphine sampling reveals underexplored chemical space in C–H methylation from high-spin ferrous complexes. (C) This work. DG = directing group.

Translation of these principles to organometallic chemistry and molecular catalysis is by comparison underexplored (Figure 1A).^19^ Spin modulation is an important component of reactivity control, where access to a lower spin state is facilitated by the introduction of an exogenous ligand to a higher-spin metal center, thus unlocking the desired reactivity by circumventing the spin blocking effect. In this context, transition-metal mediated C–H functionalization reactions were targeted given the potential applications in synthesis,^20–28^ and the dominance of low-spin precious metal complexes that operate by predictable two-electron redox pathways.^29–33^ C–H functionalization with earth-abundant first-row transition metals has become increasingly appealing given the advantages in terrestrial abundance,^34^ cost,^35^ and biocompatibility.^36,37^ However, there is limited precedent demonstrating the effect of metal spin states and the corresponding strategies for reactivity control, especially with organometallic iron complexes.^35,38–42^

Prior studies on iron-mediated C–H functionalization have principally focused on low-spin iron complexes.^43–51^ For example, our laboratory has previously reported directed C­(sp^2^)–H methylation of arenes mediated by low-spin, six-coordinate iron­(II) dimethyl complexes supported by phosphines with a narrow cone angle (θ) range of 107–132° (Figure 1B, right side).^45^ This set of ligands only accounts for a small fraction (<10%) of phosphines based on the sampling of the KRAKEN database.^52^ The majority (>90%) of phosphines feature comparatively large cone angles (θ > 132°) and have been shown to prefer the formation of high-spin four-coordinate ferrous complexes that are unreactive toward C–H activation due to spin blocking (Figure 1B, left side).^45,53^ Coupled with the suggested involvement of spin crossover in iron-mediated C–H activation from computational and spectroscopic studies,^54–59^ this motivated investigations into spin modulation to unlock C–H activation reactivity from a relatively large underexplored chemical space of high-spin iron­(II) complexes.

Here we describe coordination-induced spin modulation as a mode of reactivity control to enable C–H methylation from otherwise unreactive high-spin ferrous complexes (Figure 1C). Key to this approach is the circumvention of the high kinetic barrier for substrate coordination imposed by the lack of vacant *d* orbitals on a high-spin iron­(II) center and insufficient σ-donacity of the directing group. Reversible coordination of a strongly donating spin modulator (L) generated a five-coordinate intermediate with lower spin states (*S* = 0 or 1), (bisphosphine)­(L)­Fe­(CH_3_)_2_, enabled by the stronger ligand field associated with an increased coordination number and ligand donacity. This spin modulation generated a vacant *d* orbital to allow substrate coordination, while subsequent dissociation of the labile spin modulator led to the formation of the corresponding C–H agostic complex,^45^ and eventual arene C–H methylation at ambient temperature.

## Results

### Discovery of C–H Methylation from High-Spin Iron­(II) Complexes

The high-spin, tetrahedral (dcype)­Fe­(CH_3_)_2_ (dcype ≡ 1,2-bis­(dicyclohexylphosphino)­ethane, **Fe1**) complex was selected for these studies as it was reported to be inert toward the C–H methylation of pivalophenone (**1**) at 23 or 80 °C (Scheme 1A).^45^ Phosphines were targeted as potential spin modulators due to their widespread availability, donacity, lability and precedented reactivity in iron-mediated C–H alkylation.^45–47,60–63^

**Scheme 1:**
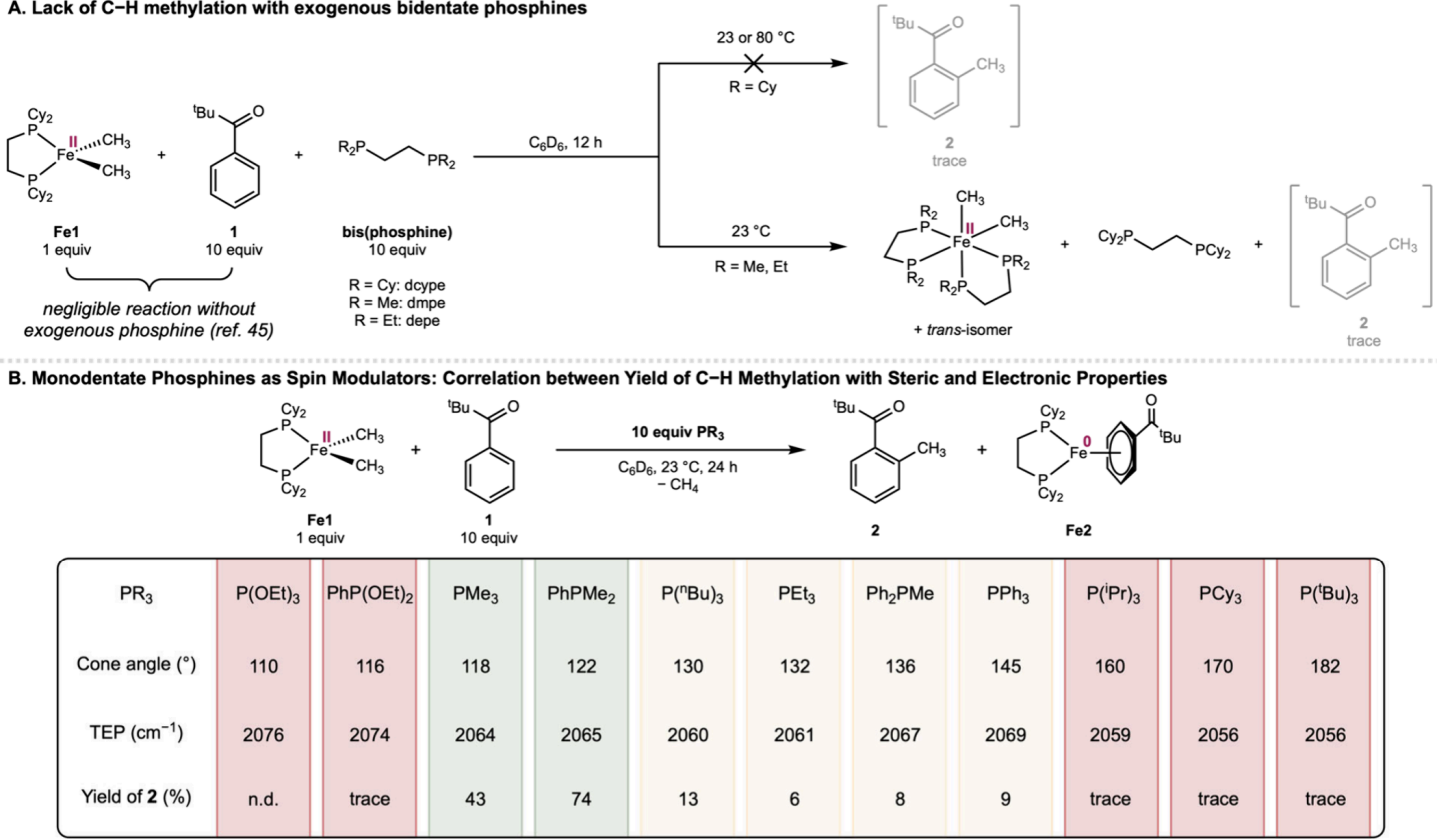
Evaluation of Exogenous Phosphines as Spin Modulators^a^

a (A) Lack of C–H methylation with exogenous bidentate phosphines. (B) Monodentate phosphines as spin modulators: correlation between yield of C–H methylation with steric and electronic properties.

Initial experiments to induce spin modulation were conducted by adding 10 equiv of the dcype ligand to a benzene-*d*_6_ solution of 1 equiv of **Fe1** and arene **1**. The desired C–H methylation product **2** was not detected after 12 h at 23 or 80 °C (Scheme 1A). Addition of sterically attenuated bis­(phosphines) such as dmpe (dmpe ≡ 1,2-bis­(dimethylphosphino)­ethane) and depe (depe ≡ 1,2-bis­(diethylphosphino)­ethane) furnished negligible quantities of **2**; instead, competing ligand substitution to form the 6-coordinate complex (dmpe)_2_Fe­(CH_3_)_2_ or (depe)_2_Fe­(CH_3_)_2_ was observed, resulting in negligible C–H methylation reactivity at room temperature.^45^ These observations inspired the exploration of monodentate phosphines as spin modulators (Scheme 1B) with increased lability over their bidentate counterparts.^64^ In the presence of 10 equiv of PPh_3_ (θ = 145°),^65^ **2** was obtained in 9% yield while switching to PMe_3_ (θ = 118°), a more sterically attenuated and more σ-donating trialkylphosphine, increased the yield to 43%. Further evaluation of phosphine ligands identified dimethylphenylphosphine (PhPMe_2_) as an optimal spin modulator. Addition of 10 equiv of PhPMe_2_ furnished **2** in 76% yield after 24 h at room temperature, along with (dcype)­Fe­(η^6^-**1**) (**Fe2**) as the organometallic product. By comparison, C–H methylation of **1** mediated by previously reported six-coordinate (depe)_2_Fe­(CH_3_)_2_ did not proceed at room temperature and required heating overnight at 80 °C.^45^

A negative correlation between the size of the added phosphine and the yield of C–H methylation product **2** was observed (Scheme 1B). Among trialkyl phosphines, the yield dropped from 43% with PMe_3_ to 13% with P­(^n^Bu)_3_ (θ = 130°) and 6% with PEt_3_ (θ = 132°), while trace amounts of **2** were observed with P­(^i^Pr)_3_, PCy_3_, and P­(^t^Bu)_3_ with θ > 160°. This lack of reactivity persisted despite the latter three being slightly stronger σ-donors as is evident from their lower Tolman electronic parameters (TEP).^65^ A similar trend was observed with PhPMe_2_ (74% yield, θ = 122°), Ph_2_PMe (8% yield, θ = 136°) and PPh_3_ (9% yield, θ = 145°) when the methyl substituent was stepwise replaced with a larger phenyl ring. Aside from steric effects, the donacity of the spin modulator was also critical (Scheme 1B). Compared with PMe_3_ (TEP = 2064 cm^–1^), PhP­(OEt)_2_ has a similar steric profile (θ = 116°) but lower donacity (TEP = 2074 cm^–1^), and produced no detectable yield of **2**. A similar lack of reactivity was observed with P­(OEt)_3_ (TEP = 2076 cm^–1^). In general, the monophosphines effective in spin modulation feature cone angles of 118–122° and TEP of 2064–2065 cm^–1^.

Spin modulation with ligands other than phosphines proved less straightforward (Figure S8). Weaker donors such as amines, pyridine, nitriles, olefins, and ethers provided low (4–14%) yields of **2** whereas strong donors such as an isonitrile and CO resulted in trace C–H methylation, potentially owing to competing ligand substitution and formation of a six-coordinate iron­(II) complex analogous to dmpe and depe. By comparison, the distinctive steric and electronic profiles of monophosphines rendered them uniquely effective as spin state modulators for unlocking C–H methylation from high-spin iron­(II) complexes.

Examination of ancillary ligand effects provided additional mechanistic insight. Bis­(phosphines) with bite angles around 85° were optimal supporting ligands for coordination-induced spin modulation (Figure S9). Using a tridentate phosphine ligand (triphos in complex **Fe5**), the *in situ* generated (triphos)­Fe­(CH_3_)_2_ afforded negligible methylation product **2**, highlighting σ-donacity and lability as the key characteristics of monodentate phosphines as effective spin-state modulators (Scheme 2). The lower lability of tridentate phosphines likely raises the barrier for the coordination of the C­(sp^2^)–H bond owing to the chelate effect,^64^ thus hindering the formation of the C–H agostic complex and the subsequent C–H methylation.

**Scheme 2:**
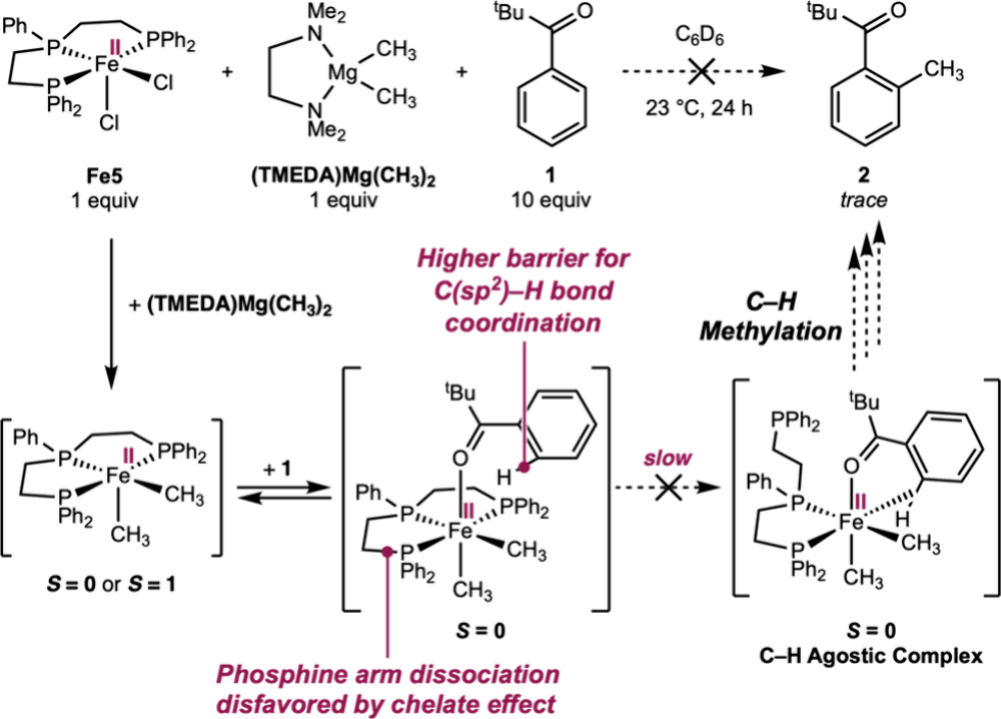
Rationale for Lack of C–H Methylation with Tridentate Phosphine Ligand

### Mechanistic Investigations

To account for the unprecedented reactivity of an apparent redox-neutral C–H functionalization from a well-defined high-spin iron­(II) alkyl complex **Fe1**, two plausible mechanistic pathways were considered (Figure 2A). Both possibilities feature the formation of a lower-spin (*S* = 0 or 1) five-coordinate complex (**Fe3** or **Fe3′**) en route to subsequent arene coordination and C–H functionalization. The key distinction is that the bidentate ligand, dcype, remains coordinated to the iron throughout Pathway 1 (left, Figure 2A), leading to a first-order rate dependence on the spin modulator PhPMe_2_. In Pathway 2 (right, Figure 2A), dcype ligand is displaced by excess PhPMe_2_, generating a five-coordinate iron­(II) complex **Fe3′** bearing only monodentate phosphines, and leading to a third-order rate dependence on PhPMe_2_. This ligand environment about iron is known to promote facile directed C–H activation at room temperature.^45,46,66^

**Figure 2:**
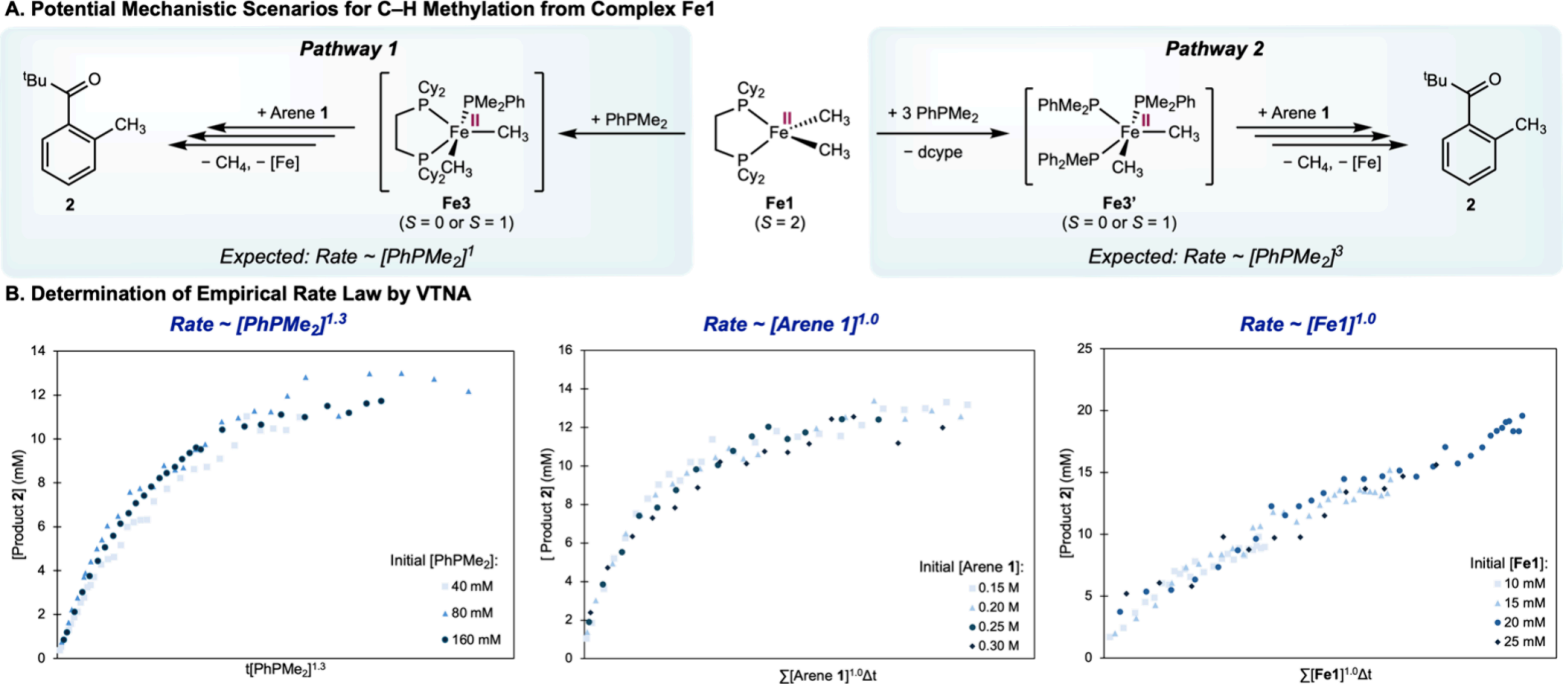
Mechanistic investigation of C–H methylation of arene **1** from high-spin iron­(II) dimethyl complex **Fe1** by kinetic studies. (A) Possible mechanistic pathways for C–H methylation from **Fe1**. (B) Determination of empirical rate law by VTNA.

To distinguish between Pathways 1 and 2, stoichiometric experiments were conducted to detect the proposed five-coordinate intermediate. Addition of 2 equiv of PhPMe_2_ to 1 equiv of **Fe1** in benzene-*d*_6_ resulted in no detectable change in the ^1^H and ^31^P­{^1^H} NMR spectra after 24 h at room temperature. By comparison, when 20 equiv of PhPMe_2_ were added, disappearance of the broad ^1^H NMR resonances characteristic of **Fe1** occurred within 15 min at room temperature (Figure S12). In the benzene-*d*_6_^1^H NMR spectrum (Figure 3), two new resonances of equal area were observed at −0.01 (triplet, labeled as “***a***”) and −0.43 ppm (quartet, labeled as “***b***”), signaling formation of a diamagnetic iron­(II) complex **Fe3** containing two inequivalent methyl ligands.^67^ In the benzene-*d*_6_^31^P­{^1^H} NMR spectrum, two roofing resonances centered at 31.2 ppm (doublet, labeled as “***A***”) and 19.7 ppm (triplet, labeled as “***B***”) were observed in a 2:1 ratio, consistent with a diamagnetic complex **Fe3** with *cis*-oriented methyl ligands (one axial and the other equatorial) in an idealized trigonal bipyramidal geometry.

**Figure 3:**
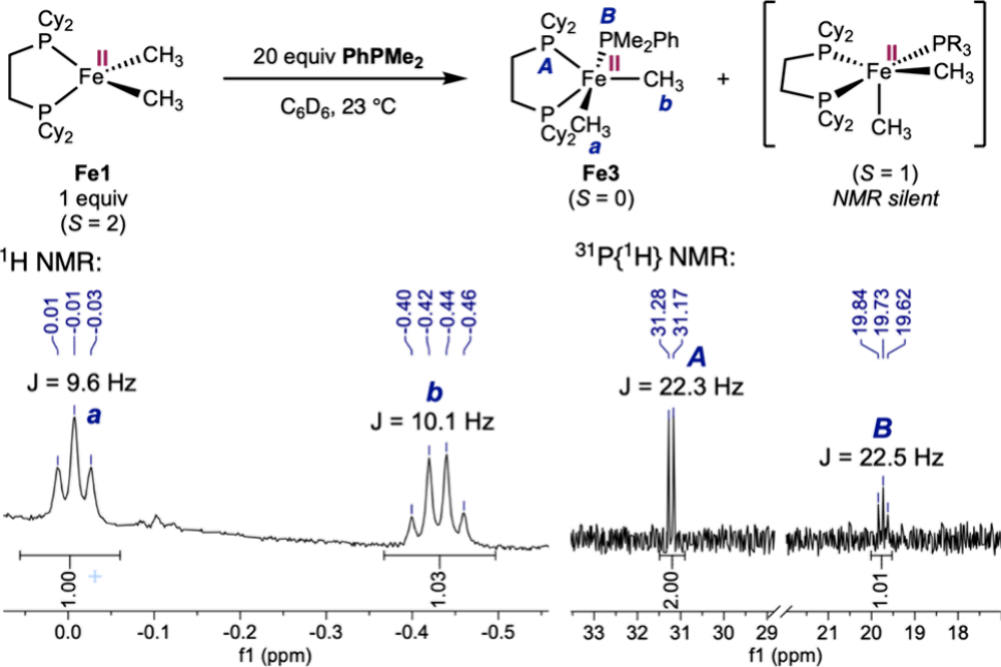
Direct observation of low-spin five-coordinate iron­(II) dimethyl complex **Fe3** by NMR spectroscopy.

In a separate titration experiment, sequential addition of 0 to 30 equiv of PhPMe_2_ to a benzene-*d*_6_ solution of **Fe1** resulted in a decrease in the effective magnetic susceptibility of the solution (Figures S17–18), consistent with an overall lowering of the spin state induced by PhPMe_2_ coordination. Together with the detection of diamagnetic **Fe3**, these observations support the involvement of coordination-induced spin modulation and corroborate Pathway 1 in Figure 2A. The experimental evidence also suggests that the formation of **Fe3** is endergonic. Meanwhile, the lack of detection of free dcype ligand by ^31^P NMR is also inconsistent with Pathway 2.

### Kinetic Studies

The difference in the stoichiometry of the PhPMe_2_ ligand involved suggests kinetic distinction of the two mechanistic scenarios. Derivation of the rate law using the steady-state approximation (Scheme S1)^68^ predicts a first and third order rate dependence on [PhPMe_2_] in Pathways 1 and 2, respectively, in the presence of a large excess of arene **1**. By applying the Variable Time Normalization Analysis (VTNA) technique developed by Burés and co-workers,^69–71^ a first-order rate dependence on [PhPMe_2_] was determined (Figure 2B) from monitoring the reaction mixture in a J. Young tube by ^1^H NMR spectroscopy. Analogous VTNA studies enabled the determination of the following empirical rate law in Equation 1:

1 $$\mathrm{Rate}=k_{\mathrm{obs}}{\left[\mathrm{arene}1\right]}^{1}{\left[\mathrm{Fe}1\right]}^{1}{\left[{\mathrm{PhPMe}}_{2}\right]}^{1}$$

Coupled with the observed primary parallel deuterium kinetic isotope effect (KIE) of *k*_H_/*k*_D_ = 4.4(3) (Figure S30), the empirical rate law is more consistent with Pathway 1 and supports the involvement of C–H cleavage in the rate-determining step. The larger magnitude of the KIE than previously observed^45^ is likely a result of the lower barrier for substrate coordination arising from the more labile monodentate PhPMe_2_ as the spin modulator.

### Stereochemical Probe

To further substantiate that the bidentate dcype ligand remains coordinated to the iron center, an analogous iron dimethyl complex supported by a chiral bis­(phosphine) ligand was synthesized to serve as a stereochemical probe. Accordingly, ((*R*,*R*)-BenzP*)­FeCl_2_ was prepared by the metalation of (*R*,*R*)-BenzP* ligand with FeCl_2_ (Scheme 3A),^72^ followed by treatment with one equivalent of (TMEDA)­Mg­(CH_3_)_2_ in diethyl ether. Warming from –35 °C to ambient temperature followed by filtration after 30 min afforded the desired complex **Fe4**, ((*R*,*R*)-BenzP*)­Fe­(CH_3_)_2_, in 70% yield as a pale yellow crystalline solid. The identity of the **Fe4** and its idealized tetrahedral geometry were corroborated by single-crystal X-ray diffraction. The magnetic moment of μ_eff_ = 5.0(2) μ_B_ of a benzene-*d*_6_ solution of **Fe4** as measured by Evans method is in agreement with a high spin (*S* = 2) Fe­(II) center.

**Scheme 3:**
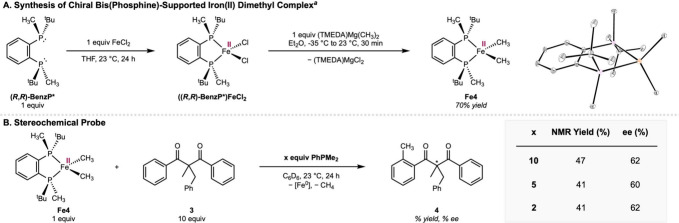
Chiral Bis­(Phosphine)-Supported Iron­(II) Dimethyl Complex as Stereochemical Probe^a^

a (A) Synthesis of chiral bis­(phosphine)-supported iron­(II) dimethyl complex. Representation of the solid-state structure of **Fe4** at 30% probability ellipsoids as determined from single-crystal X-ray diffraction. H atoms are omitted for clarity. (B) Stereochemical probe.

Treatment of a benzene-*d*_6_ solution of **Fe4** with 10 equiv of the prochiral arene **3** in the presence of 10 equiv of PhPMe_2_ (Scheme 3B) at ambient temperature for 24 h resulted in the formation of the monomethylated and desymmetrized arene **4** in 47% assay yield and 62% ee as determined by chiral supercritical fluid chromatography. The observation of a significant enantiomeric excess is consistent with the coordination of the bidentate phosphine to the iron center throughout the C–H methylation reaction (Pathway 1 in Figure 2A).

When the loading of added PhPMe_2_ ligand was reduced from 10 to 5 and then to 2 equiv, little variation in the yield (41–47%) and the enantioselectivity (60–62%) of arene **4** was observed. These observations not only provide further support for Pathway 1, but also demonstrate that the non-enantioselective Pathway 2, where the chiral bis­(phosphine) would have completely dissociated, is unlikely to occur concurrently with Pathway 1. If the latter were true, the rate dependence of Pathway 2 on the concentration of PhPMe_2_ ligand would have resulted in a negative correlation between the ee of the arene **4** formed and the PhPMe_2_ ligand loading. Overall, the combination of mechanistic evidence from experiments in Figures 2–3 and Scheme 3 supports the formation of a lower-spin (*S* = 0 and/or 1) five-coordinate iron­(II) complex P_2_(L)­Fe­(CH_3_)_2_ as a result of coordination-induced spin modulation and as a key precursor to subsequent C–H activation and functionalization.

### Computational Studies

The rationale behind the observed correlation between the steric parameters of the spin modulator and C–H methylation reactivity was further supported by DFT computations (Table 1). As evident from the mechanistic studies above, the formation of the five-coordinate monophosphine-chelate **Fe3** was essential to provide a lower-energy pathway by spin lowering to enable subsequent arene C–H methylation. Therefore, the thermodynamic accessibility of the analogous five-coordinate intermediate with different phosphines, **Fe3–PR**_**3**_, was computed as the Gibbs free energy change of the monophosphine association, ΔG_assoc_, for three trialkyl phosphines. As with PhPMe_2_, **Fe3–PR**_**3**_ was computed to have a triplet ground state with a higher-lying but thermally accessible singlet excited state. From PMe_3_ to PEt_3_ and then to P^i^Pr_3_, ΔG_assoc_ became increasingly endergonic at +3.0, + 7.6 and +18.0 kcal/mol, respectively, which showed strong correlation with the yields of **2** (43%, 6% and trace). The improved performance of PhPMe_2_ over PMe_3_, despite the former being bulkier and less donating, was attributed to its ability to form a stabilizing CH-π interaction between the C­(sp^2^)–H bond of pivalophenone **1** and the π-system of the PhPMe_2_ phenyl ring in the singlet state,^73,74^ as evident from the optimized structure of ^**1**^ **[Int1]** (Figure S32).

**Table 1:**
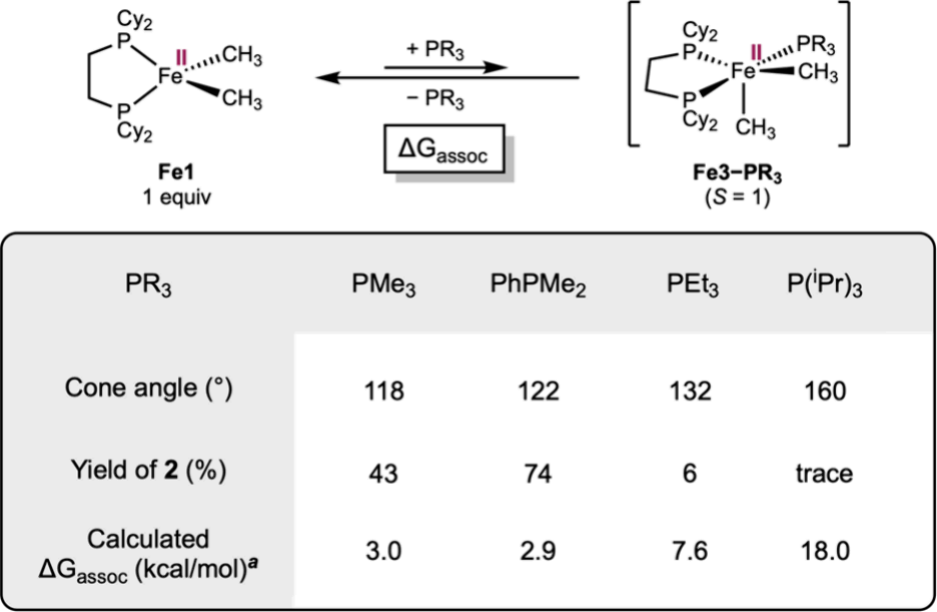
Correlation between Thermodynamic Accessibility of Five-Coordinate Intermediate **Fe3**–PR_3_ and Yield of C–H Methylation Product **2**

PR_3_	PMe_3_	PhPMe_2_	PEt_3_	P(^i^Pr)_3_
Cone angle (°)	118	122	132	160
Yield of **2** (%)	43	74	6	trace
Calculated ΔG_assoc_ (kcal/mol)^a^	3.0	2.9	7.6	18.0

a Based on DFT computations performed at (U)­TPSSh/def2-TZVPP/SMD­(benzene)//(U)­TPSS/def2-TZVP level of theory.

Additional mechanistic support for the critical role of coordination-induced spin modulation in enabling the C–H methylation of arene **1** starting from the high-spin complex **Fe1** was illustrated through the computed potential energy surface (PES) (Figure 4). Complex **Fe1** was computed to have a quintet ground state ^**5**^ **[Fe1]** with an idealized tetrahedral geometry (τ_4_’ = 0.89),^75^ consistent with all of the experimental data.^45,53^ A thermally accessible triplet excited state ^**3**^ **[Fe1]** (+0.1 kcal/mol) was located, along with a substantially higher-energy singlet excited state ^**1**^ **[Fe1]** (+17.9 kcal/mol). The coordination of PhPMe_2_ to **Fe1** to form the five-coordinate intermediate **Fe3** was endergonic by 2.9 kcal/mol. The triplet ground state ^**3**^ **[Fe3]** features a distorted square pyramidal geometry (τ_5_ = 0.38).^76^ A thermally accessible singlet state ^**1**^ **[Fe3]** (+5.9 kcal/mol) lies 3.0 kcal/mol above ^**3**^ **[Fe3]**. The computed data are consistent with the observation of a diamagnetic complex by NMR spectroscopy only upon the addition of a large excess of PhPMe_2_ (Figure 3). This provides further support for the role of PhPMe_2_ in enabling C–H functionalization reactivity through spin modulation of the iron center.

**Figure 4:**
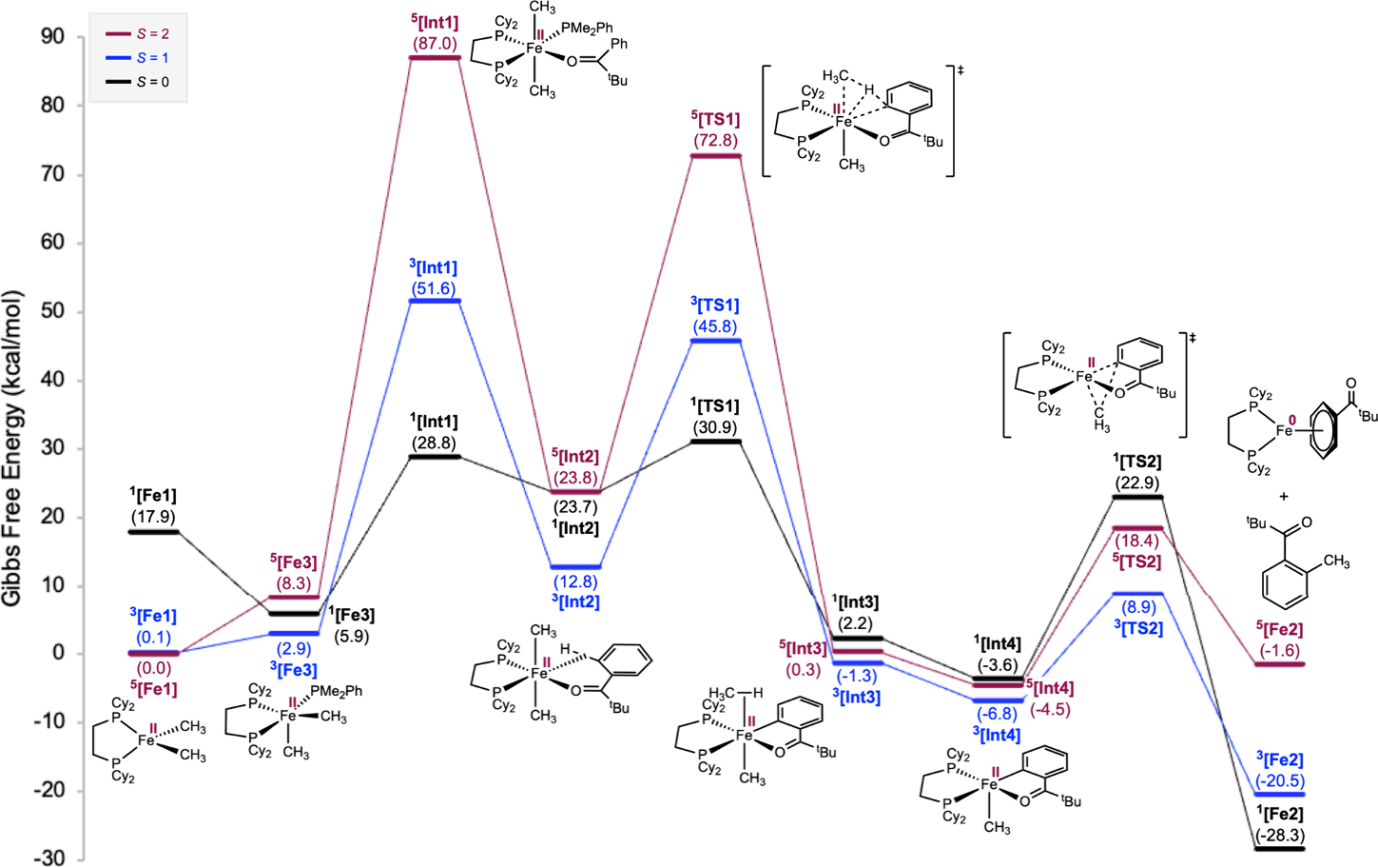
Computational insights on mechanism of spin-modulation–enabled C–H methylation starting from **Fe1** at the (U)­TPSSh/def2-TZVPP/SMD­(benzene)//(U)­TPSS/def2-TZVP level of theory.

The minimum energy crossing point (MECP) for the spin crossover from ^**5**^ **[Fe1]** to ^**3**^ **[Fe3]** during PhPMe_2_ coordination was located at +13.9 kcal/mol, corresponding to a half-life of 2 ms at 23 °C, consistent with the experimental observations in Figure 3. In comparison, the MECP from the quintet surface (^**5**^ **[Fe1]**) to the singlet surface (^**1**^ **[Fe3]**) was calculated to be +21.5 kcal/mol and likely less favored compared to the former.

Coordination of arene **1** to the five-coordinate **Fe3** generates the 6-coordinate chelate **Int1** through a triplet-to-singlet spin crossover with an accessible MECP located at +7.4 kcal/mol. While attempting to locate the transition state for this step, relaxed surface scans suggested a barrierless conversion from **Fe3** to **Int1**. The singlet ground state ^**1**^ **[Int1]** (+28.8 kcal/mol) is strongly favored over the triplet and quintet states (+51.6 and +87.0 kcal/mol). Attempts to converge to a 6-coordinate intermediate on either the triplet or quintet PES were unsuccessful. Instead, the optimized geometries of ^**3**^ **[Int1]** and ^**5**^ **[Int1]** suggested a complete dissociation of the ketone group in arene **1**, evident from the long Fe···O distances of 4.389 Å and 4.089 Å, respectively, in contrast to ^**1**^ **[Int1]** where d­(Fe–O) = 2.084 Å (Figure S32). The geometric data provide additional evidence for the substantial energetic cost for substrate coordination to a high-spin iron­(II) center, a consequence of spin blocking.

From ^**1**^ **[Int1]**, dissociation of the labile PhPMe_2_ ligand enables the chelation of one *ortho* C­(sp^2^)–H bond in arene **1** to the iron­(II) center to form the C–H σ-agostic complex ^**1**^ **[Int2]** (+23.7 kcal/mol) which is exergonic by 5.1 kcal/mol. The Fe···H distance was found to be 1.777 Å in ^**1**^ **[Int2]**, while the formation of C–H σ-agostic interaction was disfavored on the triplet and quintet PES, as suggested by d­(Fe···H) of 2.503 Å and 2.456 Å in ^**3**^ **[Int2]** and ^**5**^ **[Int2]**, respectively (Figure S33). This is consistent with the weakening of the ligand field resulting from replacing a more σ-donating phosphine with a less σ-donating C–H bond as the ligand, leading to the population of antibonding molecular orbitals and the weaker backbonding ability of the metal center in the triplet and quintet states.

The subsequent C–H cleavage occurred preferentially on the singlet surface, proceeding through a concerted four-membered transition state ^**1**^ **[TS1]**, consistent with a σ-complex assisted metathesis (σ-CAM) pathway.^77^ The calculated barrier of 30.9 kcal/mol relative to ^**5**^ **[Fe1]** was 3.5 kcal/mol lower than that for C–H methylation starting from the diamagnetic (depe)_2_Fe­(CH_3_)_2_ complex, which is consistent with the observed difference in the temperatures required for C–H methylation: 23 °C for **Fe1** (with added monodentate phosphines as the spin modulator) and 80 °C for (depe)_2_Fe­(CH_3_)_2_.^45^ Coupled with a calculated primary deuterium KIE of *k*_H_/*k*_D_ = 2.3, the calculated PES supports the C–H cleavage as the rate-determining step, consistent with the experimental findings described above. It is noted that the computed barrier (30.9 kcal/mol) is higher than expected (∼25 kcal/mol) based on the experimental observations. This overestimation has been observed and reported with related iron compounds, suggesting this is a consistent and systematic error associated with the functional and the computational method.^45,78^

From the cyclometalated iron complex **Int3**, dissociation of methane gave rise to the five-coordinate **Int4**, from which facile C–C reductive elimination proceeded preferentially through the triplet transition state ^**3**^ **[TS2]** (+8.9 kcal/mol) to afford the methylated arene **2** and diamagnetic iron(0) complex ^**1**^ **[Fe2]** with a ΔG of −28.3 kcal/mol for the overall transformation.

## Discussion

Mechanistic studies on iron­(II)-catalyzed C–H alkylation and arylation of arenes bearing bidentate monoanionic directing groups have previously been reported by Gutierrez, Neidig, Ackermann, and others.^40,57–59,79^ The C–H activation step commonly features the formation of a low-spin (*S* = 0) cyclometalated iron­(II) intermediate from a high-spin (*S* = 2) tetrahedral iron­(II) bromide complex, in the presence of a Grignard or organoaluminum reagent and a bidentate phosphine (Scheme 4, top half). This contrasts with the lack of C–H activation reactivity with high-spin tetrahedral iron­(II) dimethyl complexes such as (dcype)­Fe­(CH_3_)_2_.^45^

**Scheme 4:**
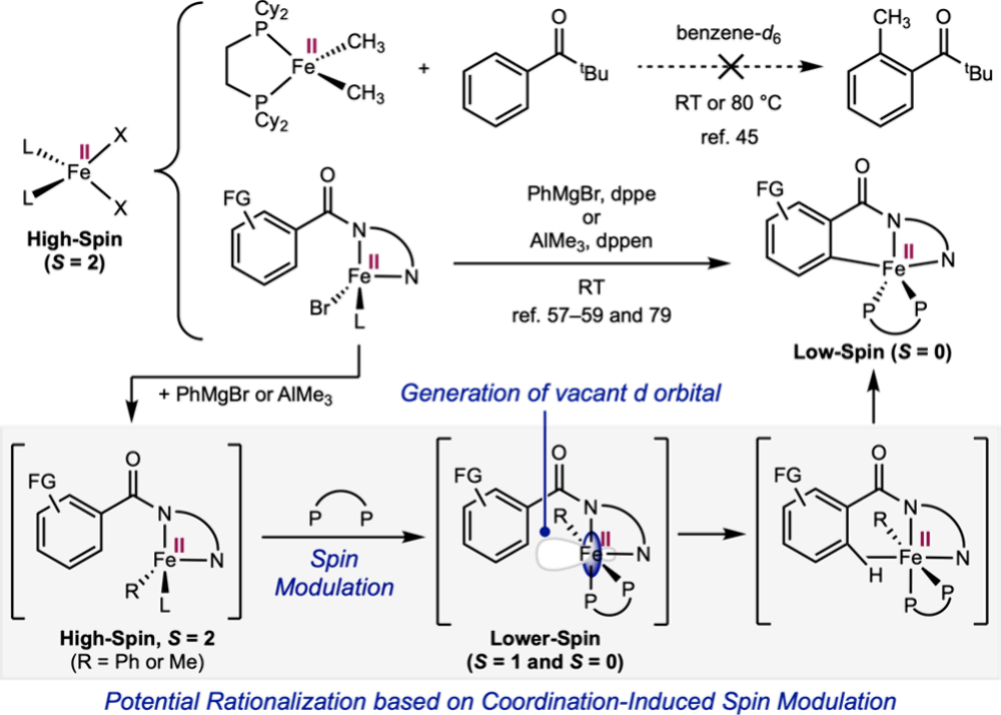
Potential Rationalization of Contrasting C–H Activation Reactivity by High-Spin Tetrahedral Iron­(II) Complexes L_2_FeX_2_ Based on Coordination-Induced Spin Modulation

The concept of coordination-induced spin modulation demonstrated here may serve as a framework to rationalize the seemingly divergent reactivity of high-spin L_2_FeX_2_ complexes and offer insights into the likely identity of the C–H activating intermediate (Scheme 4, bottom half). It may be reasoned that the transmetalation from the iron­(II) bromide likely generates a high-spin (*S* = 2) iron alkyl/aryl complex, while coordination of the bidentate phosphine (dppe or dppen) induces the lowering of the spin state of the iron­(II) center, analogous to our observations above. The generation of a vacant *d* orbital then likely enabled subsequent coordination and cleavage of the C­(sp^2^)–H bond.

## Conclusions

In summary, the concept of spin modulation as a means to promote C–H methylation reactivity has been demonstrated with organometallic iron compounds. Reversible coordination of exogenous ligands as spin modulators to high-spin four-coordinate 14-electron iron­(II) dimethyl complexes resulted in the formation of a low-to-intermediate spin five-coordinate iron­(II) complex. The adjustment of the spin state of the iron was key to overcoming spin blocking and unlocking facile redox-neutral arene C–H methylation from an underexplored class of four-coordinate high-spin iron­(II) complexes. This mechanistic pathway was corroborated by mechanistic experiments, stereochemical probes, kinetics, and computational investigations. Key steric, electronic, and geometric properties of monodentate phosphines as optimal spin state modulators were identified, and the applicability of the concept of coordination-induced spin modulation to different ancillary ligand classes was also demonstrated. Overall, the utilization of spin modulation as a strategy of tuning the reactivity of organometallic complexes provides the foundation for the future development of organometallic catalysis and bond activation, particularly with earth-abundant first-row transition metals.

## Methods

### General Procedure for C–H Methylation of Pivalophenone with High-Spin Fe­(II) Complexes

In a typical experiment, 1 equiv of a bis­(phosphine) iron­(II) dimethyl complex was added as a benzene-*d*_6_ solution to a J. Young NMR tube containing 1 equiv of 1,3,5-trimethoxybenzene, 10 equiv of dimethylphenylphosphine, and 10 equiv of pivalophenone **1** inside a N_2_-filled glovebox. The J. Young NMR tube was sealed, allowed to stand at room temperature (25 °C) for 16 h, and analyzed by quantitative ^1^H NMR spectroscopy through integration of the known product NMR resonances against the 1,3,5-trimethoxybenzene internal standard.

### Density Functional Theory (DFT) Calculations

DFT calculations were performed with the ORCA 5.0.3 program package. Geometry optimizations and single-point calculations were carried out using the TPSSh functional in combination with Grimme’s dispersion correction D3BJ. Ahlrichs’ all-electron Gaussian triple-ζ basis set def2-TZVP was employed on all atoms. Auxiliary basis sets were chosen to match the orbital basis. The RIJCOSX approximation was used to accelerate the calculations. Stationary points were identified as intermediates or transition states by frequency calculations and the presence or absence of a single imaginary frequency. Intrinsic reaction coordinate (IRC) analyses were carried out to ensure all transition states connect the appropriate starting materials and products. Single-point calculations were conducted using the optimized geometries with a larger DKH-def2-TZVPP basis and the SMD implicit solvation model for benzene. Final Gibbs free energies were then obtained by using the single-point electronic energies and the thermal corrections from the frequency calculations. The optimization of minimum energy crossing points (MECP) was conducted by using the SurfCrossOpt command. Molecular graphics and analyses were performed with UCSF Chimera, developed by the Resource for Biocomputing, Visualization, and Informatics at the University of California, San Francisco, with support from NIH P41-GM103311.

## Supplementary Material









## Notes

Preparation of iron complexes and organic substrates, optimization of spin modulator, mechanistic studies, kinetic data, X-ray crystallographic data, computational analyses, and references (PDF)
Crystal structure of **Fe1** (CIF)
Crystal structure of **Fe4** (CIF)
Cartesian coordinates for calculated structures (ZIP)

## Notes

Deposition Numbers 2505728 and 2515364 contain the supplementary crystallographic data for this paper. These data can be obtained free of charge via the joint Cambridge Crystallographic Data Centre (CCDC) and Fachinformationszentrum Karlsruhe Access Structures service.
