# Supplementary material for: Coordination-Induced Spin Modulation: Overcoming Spin Blocking in C–H Methylation with High-Spin Ferrous Complexes
Source: ACS Catal. 2026 Mar 4;16(6):6088–97. doi: 10.1021/acscatal.6c01157 (PMC13001654; doi:10.1021/acscatal.6c01157)
Supplement: Supplementary file 1 [file cs6c01157_si_001.pdf]

Supporting Information

Coordination-Induced Spin Modulation: Overcoming Spin Blocking in C–H Methylation with High-Spin Ferrous Complexes

*Tianyi Zhang,[a] Matthew V. Pecoraro,[a] and Paul J. Chirik[a]**

[a] Department of Chemistry, Princeton University
Princeton, New Jersey 08544, United States

pchirik@princeton.edu

Table of Contents

I. General Considerations S3

II. Preparation of Iron Complexes S7

III. Preparation of Organic Substrates S10

IV. Optimization of the Spin Modulator S14

IV.1 Evaluation of Sterics and Electronics of Spin State Modulators S14
IV.2 Non-Phosphine Exogenous Ligands as Spin State Modulators S16
IV.3 Effect of Bite Angle of Ancillary Ligands S17
IV.4 Spin State Modulation with (N,N)-Ancillary Ligands S18

V. Mechanistic Studies S19

V.1 Direct Observation of Five-Coordinate, Lower-Spin Intermediate S19
V.2 NMR Titration Experiment by Evans Method S22
V.3 Chiral Bis(Phosphine) Iron(II) Complexes as Stereochemical Probe S24

VI. Kinetic Data S29

VI.1 Kinetic Distinction between Pathway 1 and Pathway 2 S29
VI.2 Determination of Empirical Rate Law S30
VI.3 Parallel Deuterium Kinetic Isotope Effect Experiments S35

VII. X-Ray Crystallographic Data S36

VIII. Computational Analyses S39

VIII.1 Sample Geometry Optimization Input File S44
VIII.2 Sample Transition State Geometry Optimization Input File S45
VIII.3 Sample Single Point Calculation Input File S46
VIII.4 Coordinates and Energies S47

IX. References S50

I. General Considerations

All air- and moisture-sensitive manipulations were carried out using vacuum line, Schlenk and cannula techniques or in an MBraun inert atmosphere nitrogen dry box unless otherwise noted. All glassware was stored in a pre-heated oven (≥ 150° C) prior to use. Pentane, hexane, benzene, toluene, diethyl ether, and tetrahydrofuran used for air- and moisture-sensitive manipulations were dried and deoxygenated using literature procedures.[1] C_6D_6 used for NMR spectroscopy was distilled from sodium metal and stored over 4 Å molecular sieves. Tetramethylethylenediamine (TMEDA) and 1,2-dimethoxyethane (DME) used for stoichiometric studies were dried using sodium-benzophenone ketyl,[2] vacuum transferred and stored over 4 Å molecular sieves prior to use. Celite, alumina, silica, and glass fiber filter paper were dried at 180 ºC under vacuum for 3 days prior to use in the glovebox.

Pivalophenone (arene **1**) and *tert*-butylnitrile (^{t}BuCN) was dried over calcium hydride under vacuum for at least 48 hours, filtered through dry alumina, and stored over 4 Å molecular sieves in the glovebox prior to use. Solid reagents, including 1,3,5-trimethoxybenzene (TMB), 1-adamantyl isocyanide (AdNC), 4-(dimethylamino)pyridine (DMAP), triphenylphosphine (PPh_3), tricyclohexylphosphine (PCy_3), and tri-*tert*-butylphosphine (P^tBu_3), were dried at room temperature on a high-vacuum line (< 10 mtorr) for at least 24 hours prior to use in the glovebox. Air-sensitive liquid phosphine ligands, including trimethylphosphine (PMe_3), dimethylphenylphosphine ($PhPMe_2$), methyldiphenylphosphine (Ph_2PMe), triethylphosphine (PEt_3), tri-*n*-butylphosphine ($P(^nBu)_3$), triisopropylphosphine ($P(^iPr)_3$), triethylphosphite ($P(OEt)_3$), diethyl phenylphosphonite ($PhP(OEt)_2$), 1,2-bis(dimethylphosphino)ethane (dmpe), and 1,2-bis(diethylphosphino)ethane (depe), were purchased from commercial sources (Sigma-Aldrich®, Oakwood Chemicals®, Ambeed®, TCI America®, Combi-Blocks®, AABlocks®, Strem Chemicals®, or ChemScene®), and stored over 4 Å molecular sieves in the glovebox prior to use. CO gas was purchased from Airgas® and passed through a column of 4 Å molecular sieves before being introduced to glassware. Ethylene gas was purchased from Sigma-Aldrich®,

vacuum-transferred into and stored in an oven-dried Schlenk bomb over 4 Å molecular sieves for at least 48 hours before being introduced to glassware. The following compounds were prepared according to literature procedures: (TMEDA)Mg(CH_3)$_2$,[3] **Fe1**,[3] (dcypp)FeCl_2,[4] (dppm)FeCl_2,[5] (dppe)FeCl_2,[6] (dppp)FeCl_2,[5] (triphos)FeCl_2.[7]

^{1}H NMR spectra were recorded on either Bruker AVANCE 300, 400 or 500 spectrophotometers operating at 300.13 MHz, 399.8 MHz and 500.46 MHz, respectively. ^{13}C NMR spectra were recorded on either Bruker Avance 300, 400 or 500 spectrometers operating at 75.48 MHz, 100.54 MHz and 125.85 MHz, respectively. All ^{1}H and ^{13}C NMR chemical shifts are reported in ppm relative to SiMe_4 using the ^{1}H and ^{13}C chemical shifts of the solvent[8] as a standard. ^{1}H NMR data for diamagnetic compounds are reported as follows: chemical shift, multiplicity (s = singlet, d = doublet, t = triplet, q = quartet, p = pentet, br = broad, m = multiplet, app = apparent, obsc = obscured), coupling constants (Hz), integration, assignment. ^{1}H NMR data for paramagnetic compounds are reported as follows: chemical shift, integration, peak width at half height (Hz). ^{13}C NMR data for diamagnetic compounds are reported as follows: chemical shift, number of protons attached to carbon (e.g. CH_2), assignment. ^{31}P NMR spectra were recorded on either Bruker Avance 400 or 500 spectrometers operating at 161.84 MHz and 202.00 MHz, respectively, and were referenced to 85% H_3PO_4 as an external standard.

Single crystals suitable for X-ray diffraction were coated with polyisobutylene oil in the drybox, transferred to a nylon loop and then quickly transferred to either the goniometer head of a diffractometer equipped with a Bruker PHOTON III detector and Cu X-Ray tube (λ = 1.54178 Å) or a Rigaku XtaLAB Synergy-i equipped with a Mo X-ray tube (λ = 0.71073 Å) and a Cu X-ray tube (λ = 1.54178 Å). Preliminary data revealed the crystal system. The data collection strategy was optimized for completeness and redundancy using either the Bruker APEXII software suite or Rigaku CrysAlis^{Pro} software suite. The space group was identified, and the data were processed and corrected for absorption. The structures were solved using intrinsic phasing (SHELXT) and

completed by subsequent Fourier synthesis and refined by full-matrix least-squares procedures in Olex2. Unless otherwise specified, hydrogen atoms were modelled as riding atoms.

Enantiomeric excesses were determined by Chiral SFC performed by Lotus Separations, LLC, with a Mettler SFC supercritical CO_2 analytical chromatography system utilizing ColumnTek Enantiocel A4-5 columns (250 x 4.6 mm) obtained from ColumnTek, LLC. Specifically, the chiral SFC separation was conducted with a flow rate of 2.0 mL/min using 35% methanol (0.1% diethylamine) / 65% CO_2 (100 bar) as the eluent.

High-resolution mass spectra were obtained at Princeton University mass spectrometry facilities using either an Agilent 6230 TOF LC/MS, Agilent 7200 Accurate-Mass Q-TOF GC/MS or 7250 GC/Q-TOF.

Preparative thin-layer chromatography (TLC) was performed using E. Merck silica gel 60 F254 precoated glass plates (0.25 mm) and visualized by UV fluorescence quenching. Silicycle SiliaFlash® P60 Academic Silica gel (particle size 40–63 μm) was used for flash column chromatography separations.

DFT calculations were performed with the ORCA 5.0.3 program package.[9,10,11] Geometry optimizations and single-point calculations were carried out using the TPSSh functional in combination with Grimme's dispersion correction D3BJ.[12,13] Ahlrichs' all-electron Gaussian triple zeta basis set def2-TZVP was employed on all atoms.[14] Auxiliary basis sets were chosen to match the orbital basis.[15,16,17] The RIJCOSX approximation was used to accelerate the calculations.[18,19,20] Stationary points were identified as intermediates or transition states by frequency calculations and the presence or absence of a single imaginary frequency. Intrinsic reaction coordinate (IRC) analyses were carried out to ensure all transition states connect the appropriate starting materials and products.[21] Single-point calculations were conducted using the optimized geometries with a larger DKH-def2-TZVPP basis[22] and the SMD implicit solvation model[23] for benzene. Final Gibbs free energies were then obtained by using the single-point

electronic energies and the thermal corrections from the frequency calculations. The optimization of minimum energy crossing points (MECP) was conducted by using the SurfCrossOpt command based on the work of Harvey and co-workers.[24] Molecular graphics and analyses were performed with UCSF Chimera, developed by the Resource for Biocomputing, Visualization, and Informatics at the University of California, San Francisco, with support from NIH P41-GM103311.[25]

II. Preparation of Iron Complexes

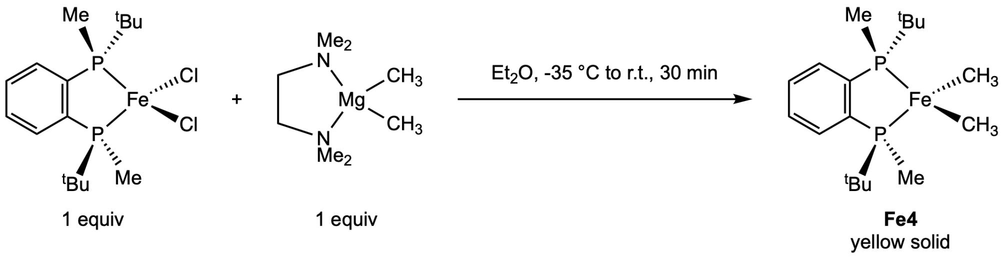

Preparation of ((*R*,*R*)-BenzP*)Fe(CH$_3$)$_2$ (Fe4). In a N_2-filled glovebox, to a suspension of 50 mg ((*R*,*R*)-BenzP*)FeCl$_2$ (0.12 mmol, 1.0 equiv) in 5 mL Et$_2$O (pre-cooled at -35 °C for 15 min), a colorless solution of 23 mg (TMEDA)MgMe$_2$ (0.13 mmol, 1.1 equiv) in 5 mL Et$_2$O (pre-cooled at -35 °C for 15 min) was added. The reaction mixture was stirred for 30 minutes at room temperature. The resultant yellow suspension was filtered through a pad of celite on top of a filter frit and the yellow filtrate was concentrated in vacuo. The resultant yellow-white solid was then re-suspended in pentane and filtered through Celite through a glass microfiber filter. Concentration of the clear yellow filtrate in vacuo, followed by recrystallization in Et$_2$O containing 1% (v/v) benzene at -35 °C afforded the desired product **Fe4** as yellow-brown crystals (32 mg, 0.087 mmol, 71% yield).

Single crystals suitable for X-ray diffraction were obtained through recrystallization at -35 °C using the vapor diffusion method, with a saturated diethyl ether solution of **Fe4** in a dram vial placed in a sealed 20 mL scintillating vial containing ~ 10 mL of *n*-pentane. The solid-state structure was determined by single-crystal X-ray diffraction (SC-XRD) and confirmed the assignment of **Fe4**. The SC-XRD structure (**Figure S1**) clearly showed an idealized tetrahedral geometry around the iron center, similar to that of (dcype)Fe(CH$_3$)$_2$ (**Fe1**).

The yellow crystals of **Fe4** were dissolved in benzene-d_6 and characterized by ^{1}H and ^{31}P{^{1}H} NMR spectroscopies. The lack of observable resonances in the ^{31}P{^{1}H} NMR spectrum (**Figure S3**) is consistent with its paramagnetism. Only trace amount of free (*R*,*R*)-BenzP* ligand was observed together with the gradual formation of black precipitate, likely due to slow decomposition due to C–C bond reductive elimination and ethane evolution.

^1H NMR (400 MHz, benzene-d_6, 23 °C): δ 106.54 (br s, Δv = 632 Hz), 10.01(br s, Δv = 703 Hz), 8.37 (br s, Δv = 141 Hz), 7.78 (br s, Δv = 107 Hz), 1.11 (br s, Δv = 198 Hz).

Magnetic Susceptibility (Evans method, 23 ºC): μ_{eff} = 5.0(2) μ_B.

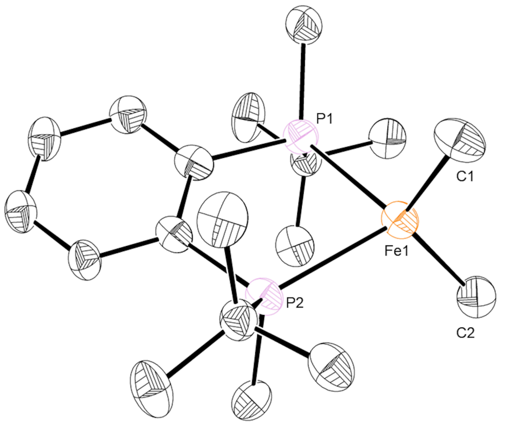

Figure S1. Representation of the solid-state structure of ((*R*,*R*)-BenzP*)Fe(CH_3)$_2$ (**Fe4**) at 30% probability ellipsoids as determined from single-crystal X-ray diffraction. H atoms are omitted for clarity. CCDC number 2515364.

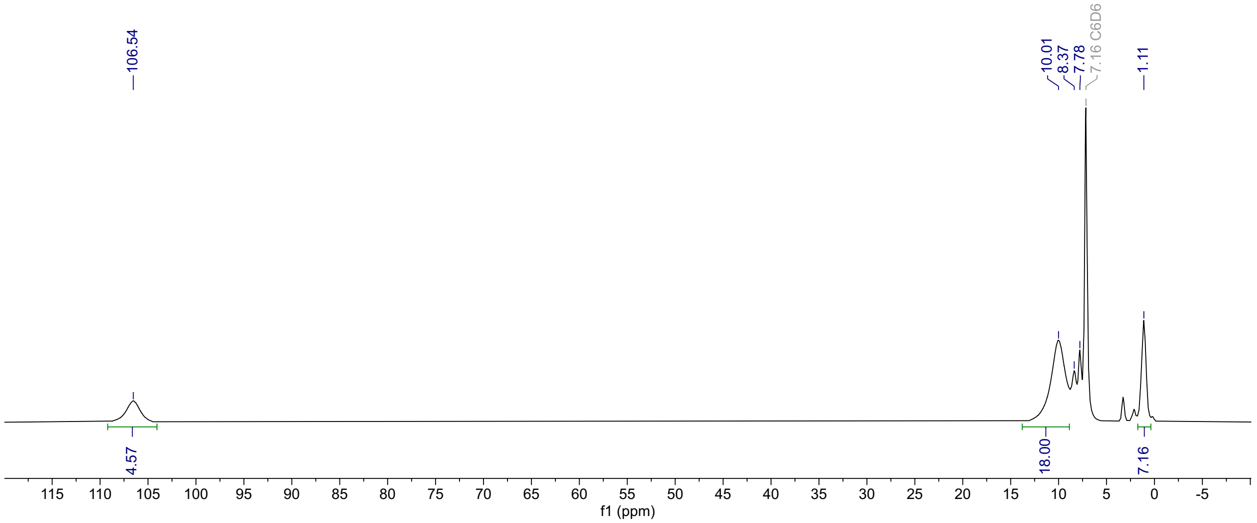

Figure S2. ^1H NMR (400 MHz, benzene-d_6, 23 °C) spectrum of complex **Fe4**. No other resonances were observed beyond this window (120 to -10 ppm).

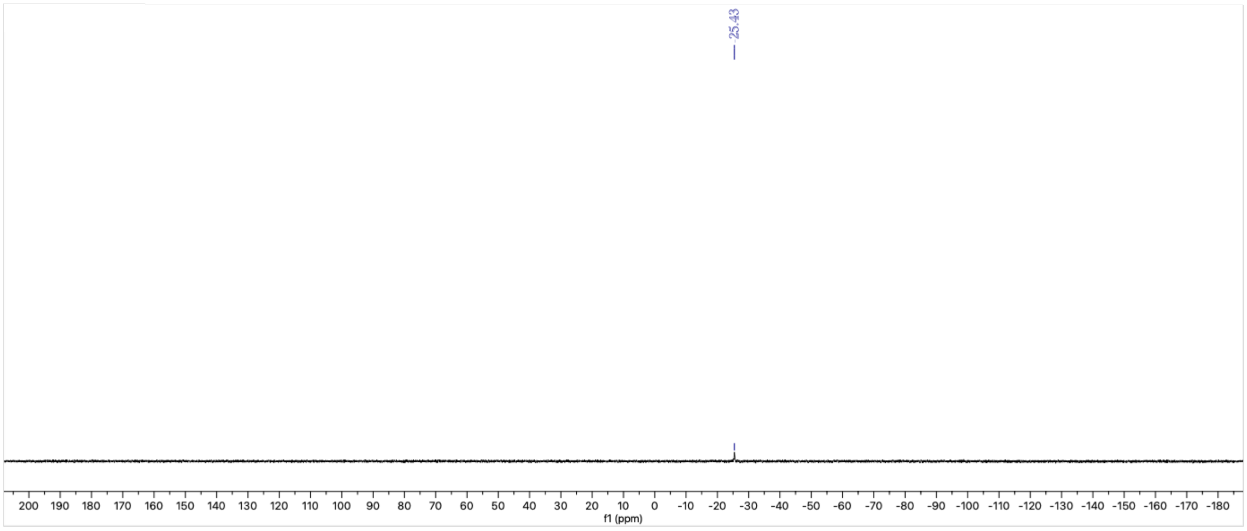

Figure S3. $^{31}P\{^{1}H\}$ NMR (162 MHz, benzene-d_6, 23 °C) spectrum of complex **Fe4**.

III. Preparation of Organic Substrates

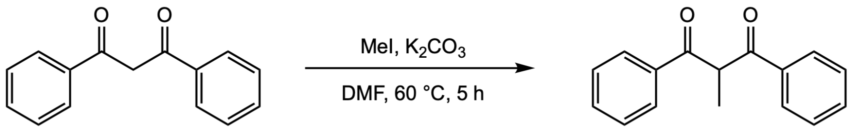

Preparation of 2-methyl-1,3-diphenylpropane-1,3-dione. Following a previously reported protocol, a 100 mL round-bottomed flask was charged with a stirring bar, 1,3-diphenylpropane-1,3-dione (5.00 g, 22.3 mmol), iodomethane (1.53 mL, 24.5 mmol) and potassium carbonate (4.62 g, 33.4 mmol), 22.3 mL of anhydrous *N*,*N*-dimethylformamide (DMF). The mixture was then heated at 60 °C for 5 hours under an N_2 atmosphere (using a N_2-filled balloon). Upon complete consumption of the substrate by analyzing an aliquot of the solution using thin-layer chromatography, the reaction was quenched by water, and the resulting suspension was extracted with ethyl acetate (3 x 30 mL) and brine (50 mL) and dried over anhydrous sodium sulfate. The residue was purified by flash column chromatography (ethyl acetate/hexanes = 5:1) to afford 2-methyl-1,3-diphenylpropane-1,3-dione as a pale-yellow solid (4.12 g, 78% yield). The analytical data were consistent with literature data that were reported previously.[26]

^{1}**H NMR** (300 MHz, $CDCl_3$, 23 ºC): δ 8.00 – 7.92 (m, 4H), 7.61 – 7.53 (m, 2H), 7.51 – 7.39 (m, 4H), 5.26 (q, *J* = 7.0 Hz, 1H), 1.61 (d, *J* = 7.0 Hz, 3H).

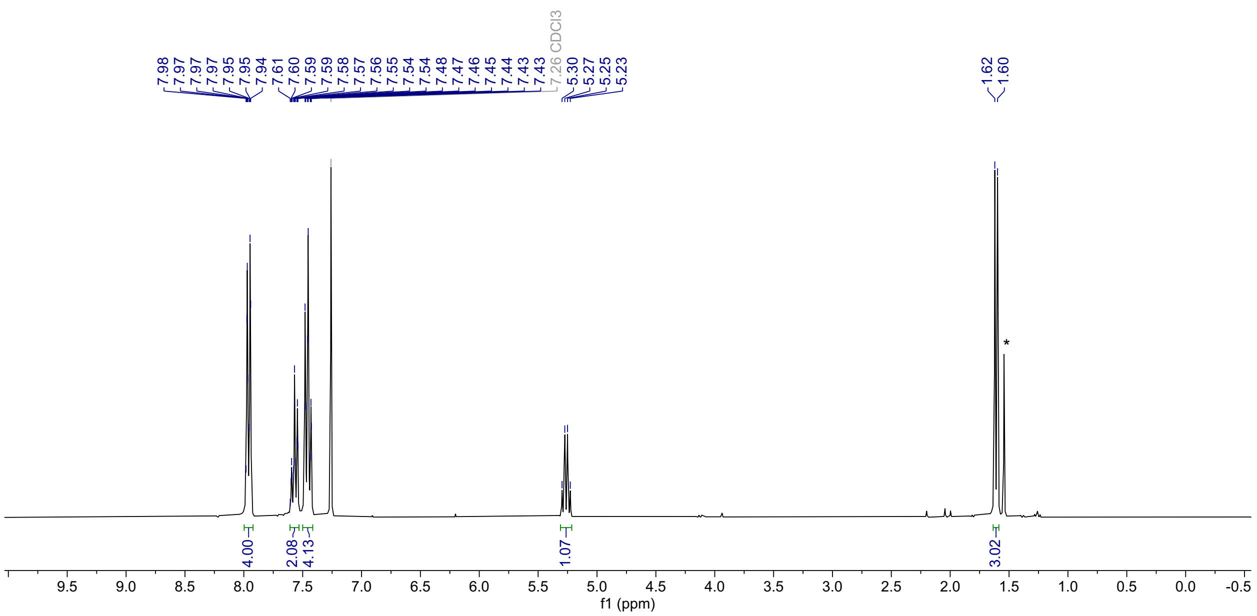

Figure S4. ^{1}H NMR (300 MHz, $CDCl_3$, 23 °C) spectrum of 2-methyl-1,3-diphenylpropane-1,3-dione after purification by silica gel column chromatography. * ≡ residual water.

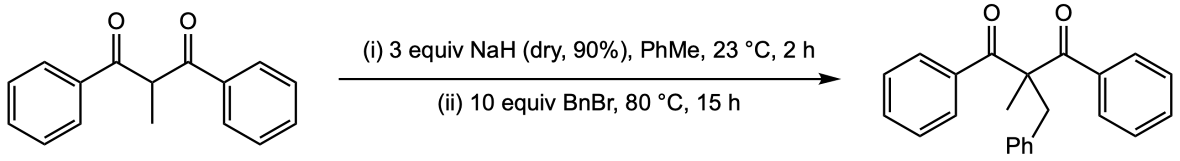

2-methyl-1,3-diphenylpropane-1,3-dione
1 equiv

3
1.1 gram white solid (80% yield)

Preparation of Stereochemical Probe 3. Using a modified version of the reported protocol,[27] 300 mg of NaH powder (12.5 mmol, 3 equiv) was weighed out in a N_2-filled glovebox, to which a solution of 1.00 g (4.2 mmol) of 2-methyl-1,3-diphenylpropane-1,3-dione in 42 ml of toluene was added. The reaction mixture was stirred for 2 h at room temperature until the evolution of hydrogen stopped. 5.0 mL of benzyl bromide (42 mmol, 10 equiv) was then added and the mixture was stirred for 15 h at 80 °C. An aqueous 10% $NaHCO_3$ (50 ml) solution was added and the organic phase was separated, washed with water, brine and dried (Na2SO4). Solvent and excess benzyl bromide were removed in vacuo. The crude product was loaded onto a pad of silica gel (L = 20 cm, D = 4 cm) and purified by flash column chromatography (dichloromethane:hexanes = 1:1), yielding 2-benzyl-2-methyl-1,3-diphenylpropane-1,3-dione (**3**) as a white solid (1.1 g, 80% yield).

The analytical data were consistent with literature data that were reported previously.[28] Compound **3** was attached to a high-vacuum Schlenk line (< 10 mtorr) and dried under vacuum for 48 hours prior to use in the glovebox.

^{1}H NMR (500 MHz, $CDCl_3$, 23 °C): δ 7.89 – 7.83 (m, 4H), 7.47 – 7.40 (m, 2H), 7.34 (t, J = 7.7 Hz, 4H), 7.22 – 7.12 (m, 3H), 6.88 – 6.81 (m, 2H), 3.61 (s, 2H), 1.57 (s, 3H).

^{13}C{^{1}H} NMR (126 MHz, $CDCl_3$, 23 °C) δ 199.5, 136.2, 136.2, 133.2, 130.8, 129.4, 128.9, 128.1, 127.0, 64.1, 43.1, 22.6.

HRMS (ESI+) *m/z* calculated for $[C_{23}H_{21}O_2]+$ ($[M+H]^+$) 329.1536, found *m/z* 329.1542.

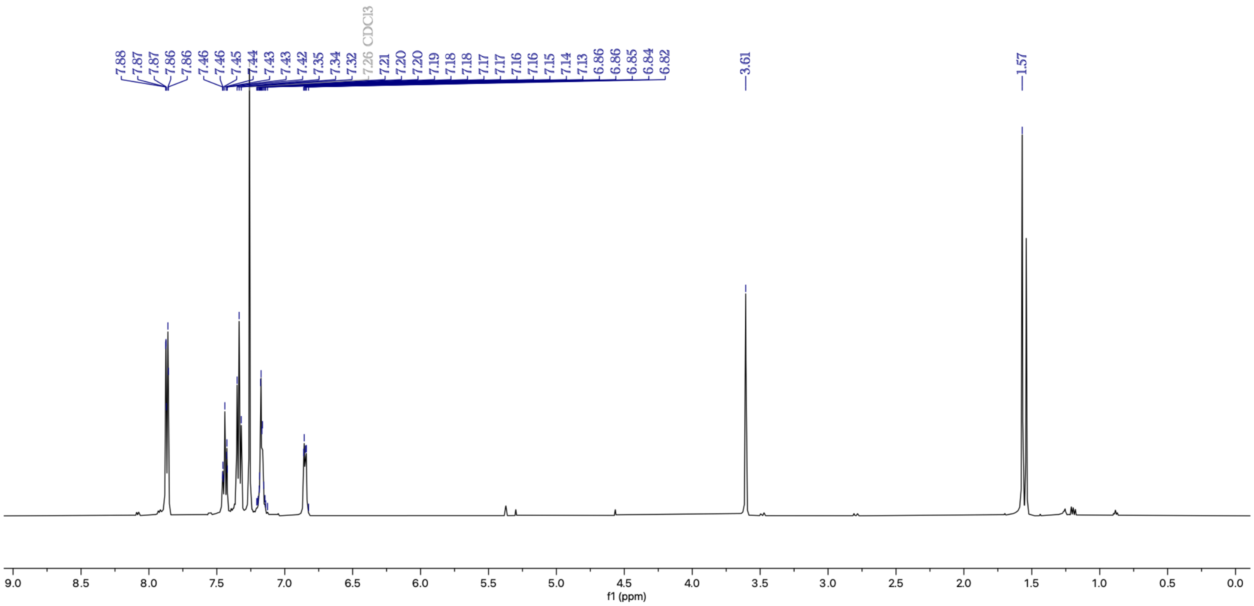

Figure S5. ^{1}H NMR (500 MHz, $CDCl_3$, 23 °C) spectrum of stereochemical probe **3** after purification by silica gel flash column chromatography.

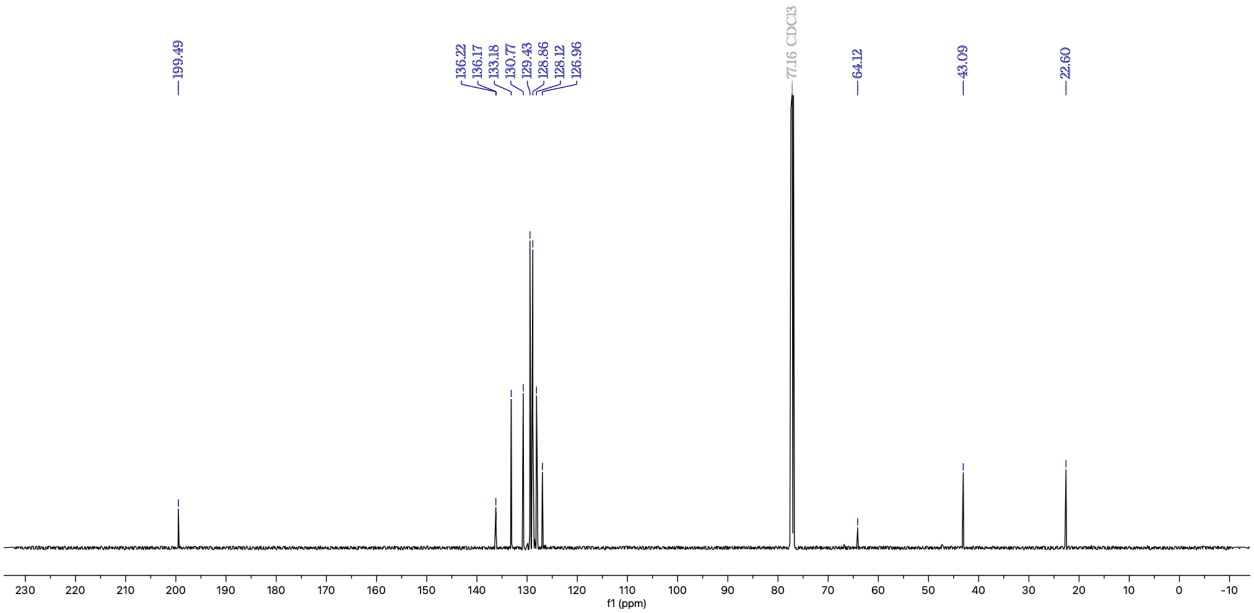

Figure S6. $^{13}C\{^{1}H\}$ NMR (126 MHz, $CDCl_3$, 23 °C) spectrum of stereochemical probe **3** after purification by silica gel column chromatography.

IV. Optimization of the Spin Modulator

General Procedure for C–H Methylation of Pivalophenone with High-Spin Fe(II) Complexes. In a typical experiment, 1 equivalent of a bis(phosphine) iron(II) dimethyl complex was added as a benzene-d_6 solution to a J. Young NMR tube containing 1 equivalent of TMB, 10 equivalents of dimethylphenylphosphine, and 10 equivalents of pivalophenone **1** inside a N_2-filled glovebox. The J. Young NMR tube was sealed, allowed to stand at room temperature (25 °C) for 16 hours, and analyzed by quantitative ^1H NMR spectroscopy. Integration of the known product NMR resonances against TMB internal standard enabled the determination of the amount of C–H methylation product formed.

IV.1 Evaluation of Sterics and Electronics of Spin State Modulators

Stoichiometric experiments were conducted based on the General Procedure above, with 5.1 mg of (dcype)Fe$(CH_3)_2$ (**Fe1**, 0.010 mmol, 1 equiv), 0.10 mmol of monodentate phosphine/phosphinite/phosphite (10 equiv), 1.7 mg of TMB (0.010 mmol, 1 equiv), 16 mg of pivalophenone **1** (0.10 mmol, 10 equiv) and 0.5 mL of benzene-d_6. Note that **Fe1** and TMB was added each as a separate 0.100 mL benzene-d_6 stock solution, made from dissolving 51 mg **Fe1** in 1.0 mL benzene-d_6 and 17 mg TMB in 1.0 mL benzene-d_6, respectively.

For the experiment with PMe_3, the contents of the J. Young tube were frozen in a liquid N_2 bath and the tube was evacuated on a high-vacuum line. 10 equivalents of PMe_3 (14.2 cmHg) were added by vacuum transfer with the assistance of a calibrated gas bulb (13.1 cm^3). The contents of the J. Young NMR tube were thawed and the progress of the C–H methylation of pivalophenone **1** was monitored by quantitative ^1H NMR spectroscopy (**Figure S7**).

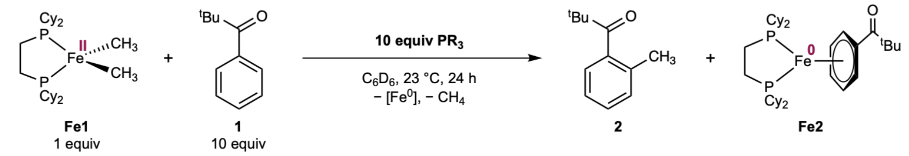

PR_3	$P(OEt)_3$	$PhP(OEt)_2$	PMe_3	$PhPMe_2$	$P(^nBu)_3$	PEt_3	Ph_2PMe	PPh_3	$P(^iPr)_3$	PCy_3	$P(^tBu)_3$
Cone angle (°)	110	116	118	122	130	132	136	145	160	170	182
TEP (cm^{-1})	2076	2074	2064	2065	2060	2061	2067	2069	2059	2056	2056
Yield of **2** (%)	n.d.	trace	43	74	13	6	8	9	trace	trace	trace

Figure S7. Evaluation of steric and electronic properties of monodentate phosphine/phosphinite/phosphite ligands on their effectiveness as spin state modulators.

IV.2 Non-Phosphine Exogenous Ligands as Spin State Modulators

In a N_2-filled glovebox, 36 mg (dcype)Fe(CH_3)$_2$ (**Fe1**, 0.070 mmol, 1 equiv), 114 mg of pivalophenone **1** (0.70 mmol, 10 equiv) and 12 mg of TMB (0.70 mmol, 10 equiv) were dissolved in 2.1 mL of benzene-d_6 and distributed evenly to seven J. Young tubes. To five of the seven J. Young tubes, 10 equivalents of the exogenous ligands L (L = DMAP, TMEDA, DME, ^{t}BuCN, AdNC) was weighed out in a 1-dram vial, dissolved in 0.2 mL of benzene-d_6 and added.

For the remaining two J. Young tubes, 0.2 mL of benzene-d_6 was added to maintain a constant total volume of 0.5 mL, and the contents of each of the two tubes were frozen by submerging the tube in liquid nitrogen, and 10 equivalents of dry ethylene or 1 equivalent of dry carbon monoxide was added at this temperature by vacuum transfer using a 13.1 cm^3 calibrated gas bulb. The contents of each tube were thawed by placing the tube in a water bath.

Reaction progress in all J. Young tubes was then followed at room temperature or 80 °C by quantitative ^{1}H NMR spectroscopy. The yield of the *ortho*-methylated arene **2** was then determined by integration relative to the TMB internal standard (**Figure S8**).

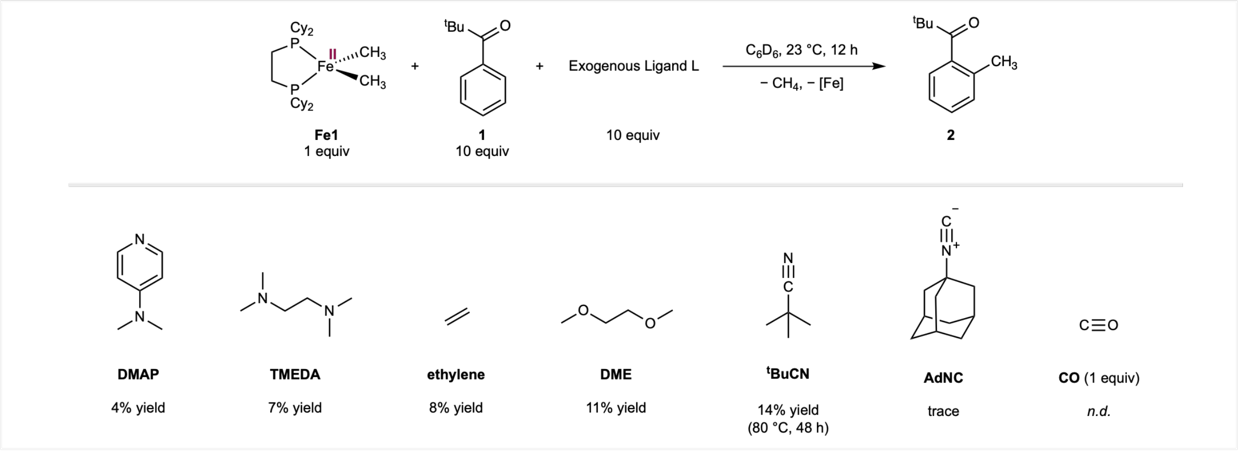

Figure S8. Performance of non-phosphine exogenous ligands as spin modulators to promote C–H functionalization mediated by high-spin iron(II) complex **Fe1**. The yield of arene **2** was measured by quantitative ^{1}H NMR spectroscopy using TMB as an internal standard.

IV.3 Effect of Bite Angle of Ancillary Ligands

In a nitrogen-filled glovebox, 1 equivalent of bis(phosphine) iron (P_2FeCl_2, 0.020 mmol, 1 equiv), 3.4 mg of (TMEDA)Mg(CH_3)$_2$ (0.020 mmol, 1 equiv), prepared from a benzene stock solution of 17 mg (TMEDA)Mg(CH_3)$_2$, were mixed in respective 1-dram vials each loaded with a magnetic stir bar, with benzene added to ensure a total volume of 0.5 mL. After 10 minutes of stirring at room temperature, 32 mg of pivalophenone **1** (0.20 mmol, 10 equiv) and 28 mg of $PhPMe_2$ (0.20 mmol, 10 equiv) dissolved in 0.5 mL benzene was added dropwise. The vial was then sealed and stirred at room temperature for 24 hours, before being taken out of the glovebox, evaporated to dryness, dissolved in 0.5 mL of chloroform-*d* containing 1 equivalent of TMB as an internal standard and characterized by quantitative ^{1}H NMR spectroscopy. The yield of the *ortho*-methylated arene **2** was then determined by integration relative to the internal standard (**Figure S9**).

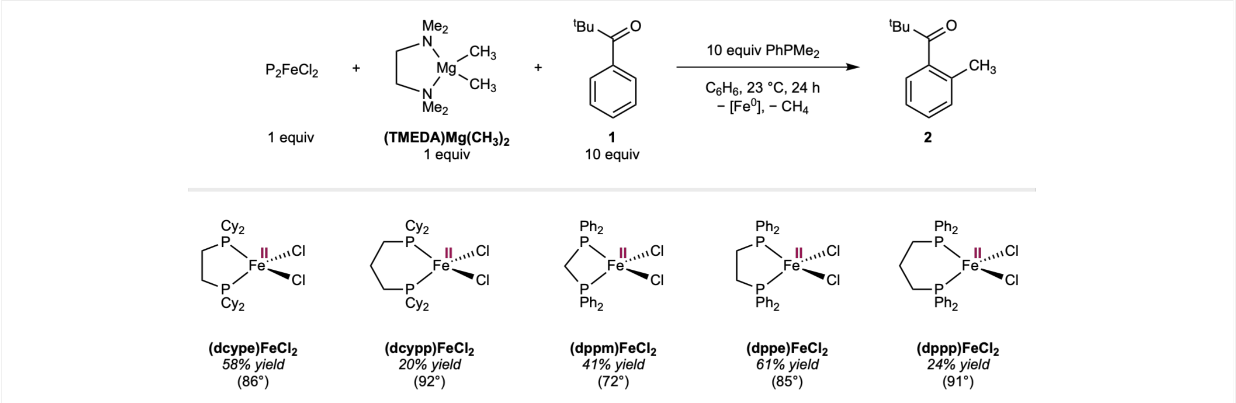

Figure S9. Effect of variation of the ancillary phosphine ligands on reactivity in spin-modulated C–H methylation. The yield of arene **2** was measured by quantitative ^{1}H NMR spectroscopy using TMB as an internal standard. Known bite angles of the bis(phosphine) ligands are included in the parentheses below each structure. [a] with 0 or 10 equivalents of $PhPMe_2$ added.

IV.4 Spin State Modulation with (N,N)-Ancillary Ligands

In a nitrogen-filled glovebox, 1 equivalent of (N,N)$FeCl_2$ (0.020 mmol, 1 equiv), 3.4 mg of (TMEDA)$Mg(CH_3)_2$ (0.020 mmol, 1 equiv) – prepared from a benzene stock solution of 17 mg (TMEDA)$Mg(CH_3)_2$ were mixed in respective 1-dram vials each loaded with a magnetic stir bar, with benzene added to ensure a total volume of 0.5 mL. After 10 minutes of stirring at room temperature, 32 mg of pivalophenone **1** (0.20 mmol, 10 equiv) and 28 mg of $PhPMe_2$ (0.20 mmol, 10 equiv) dissolved in 0.5 mL benzene was added dropwise. The vial was then sealed and stirred at room temperature for 48 hours, before being taken out of the glovebox, evaporated to dryness, dissolved in 0.5 mL of chloroform-*d* containing 1 equivalent of TMB as an internal standard and characterized by quantitative ^{1}H NMR spectroscopy. The yield of product **2** was then determined by integration relative to the TMB internal standard (**Figure S10**).

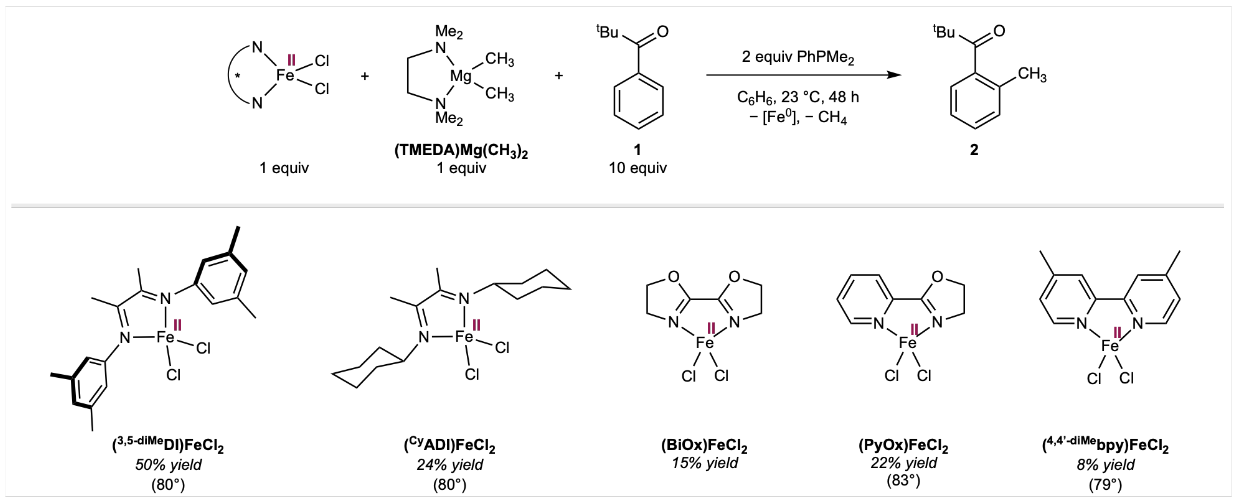

Figure S10. Evaluation of bidentate *N*,*N*-ligands as spin modulating ligands. C–H methylation of arene **1** mediated by *in situ* generated iron(II) dimethyl complexes supported by *N*,*N*-ligands. Reported bite angles of the *N*,*N*-ligands are included in the parentheses.

V. Mechanistic Studies

V.1 Direct Observation of Five-Coordinate, Lower-Spin Intermediate

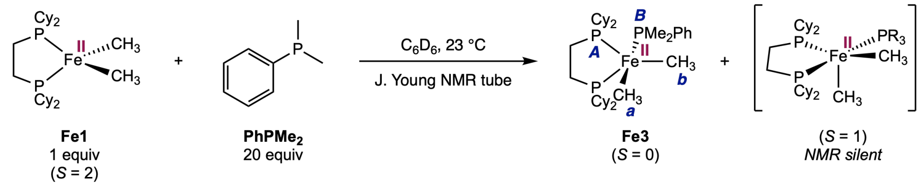

In a nitrogen-filled glovebox, 10 mg (dcype)$Fe(CH_3)_2$ (**Fe1**, 0.020 mmol, 1 equiv) was dissolved in 300 µL of benzene-d_6 solvent and transferred to an oven-dried J. Young tube. To this solution, 83 mg of $PhPMe_2$ (0.60 mmol, 20 equiv) in 200 µL of benzene-d_6 was added, resulting in an immediate color change from light yellow to orange. The contents of the tube were subsequently characterized by ^1H and $^{31}P\{^1H\}$ NMR spectroscopies at room temperature (**Figure S11–13**). Within 15 minutes, complete conversion of the high-spin complex **Fe1** and the formation of the diamagnetic (S = 0) five-coordinate intermediate **Fe3** were observed.

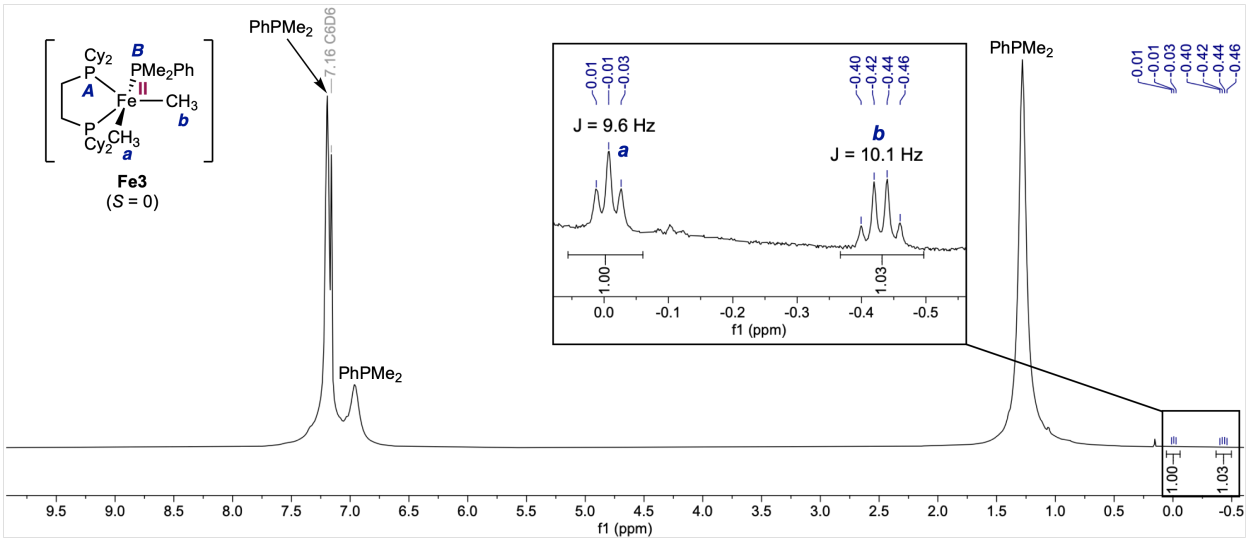

Figure S11. Representative ^1H NMR spectrum (400 MHz, benzene-d_6, 25 °C, 10.0 to -0.5 ppm) of the mixture of **Fe1** in the presence of 20 equivalents of $PhPMe_2$**.** Broadened resonances of $PhPMe_2$ were labeled.

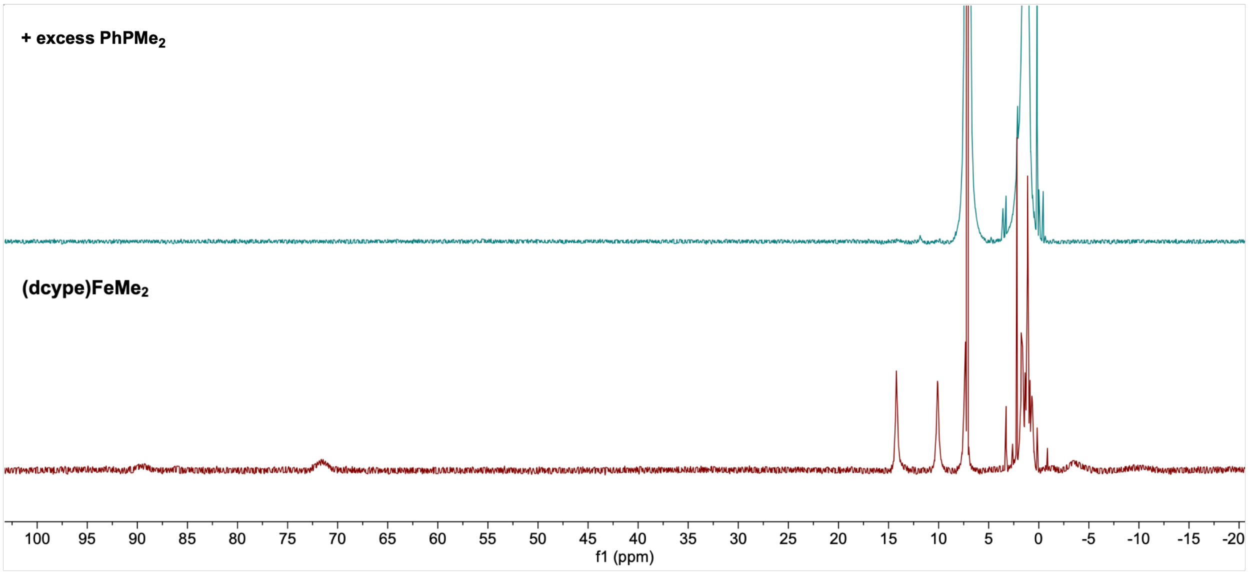

Figure S12. Representative stacked ^{1}H NMR spectra (400 MHz, benzene-d_6, 25 °C, 105 to -20 ppm) of complex **Fe1** before (bottom) and after (top) the addition of 20 equivalents of $PhPMe_2$, demonstrating the complete disappearance of the paramagnetically shifted broad resonances characteristic of complex **Fe1**.

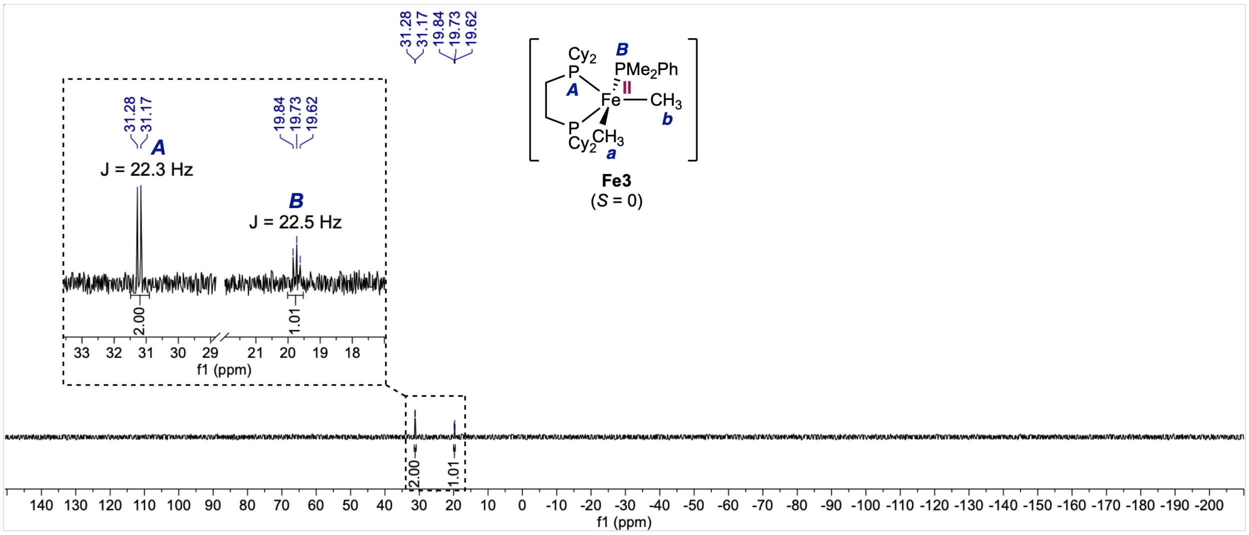

Figure S13. Representative $^{31}P\{^{1}H\}$ NMR spectrum (162 MHz, benzene-d_6, 25 °C) of the reaction mixture upon addition of 20 equivalents of $PhPMe_2$, demonstrating the formation of the diamagnetic (S = 0) five-coordinate intermediate **Fe3**.

In comparison, addition of 2 equivalents of $PhPMe_2$ to 1 equivalent of complex **Fe1** resulted in no significant conversion of **Fe1** and no detectable formation of the diamagnetic (S = 0 spin isomer) complex **Fe3**. Broadening of the $PhPMe_2$ resonance in the $^{31}P\{^{1}H\}$ NMR spectrum (**Figure S15**)

is consistent with dynamic association of $PhPMe_2$ with complex **Fe3**. Overall, this is consistent with five-coordinate intermediate **Fe3** being slightly energetically uphill from **Fe1** and $PhPMe_2$.

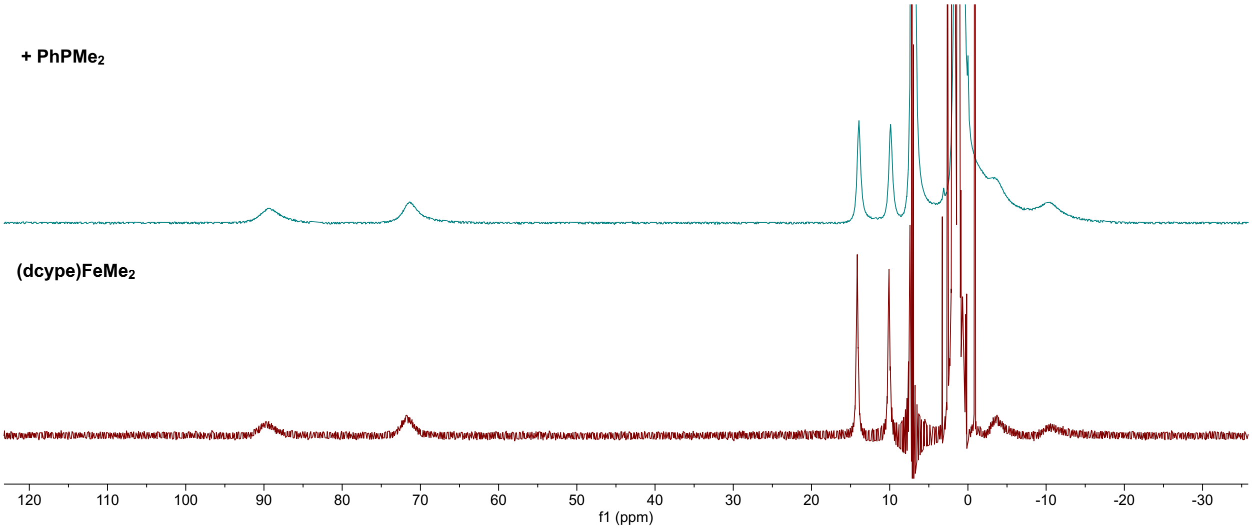

Figure S14. Representative stacked ^{1}H NMR spectrum (400 MHz, benzene-d_6, 25 °C, 120 to -30 ppm) of (dcype)Fe(CH_3)$_2$ (complex **Fe1**) before (bottom) and after (top) the addition of 2 equivalents of $PhPMe_2$.

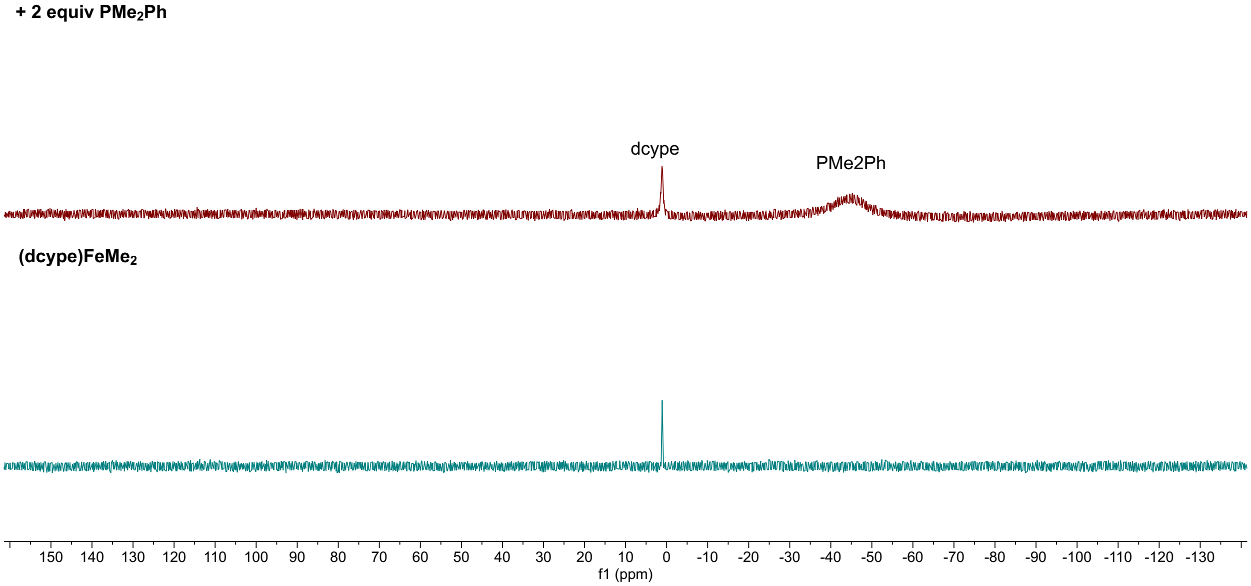

Figure S15. Representative stacked $^{31}P\{^1H\}$ NMR spectrum (162 MHz, benzene-d_6, 25 °C) of (dcype)Fe(CH_3)$_2$ (complex **Fe1**) before (bottom) and after (top) the addition of 2 equivalents of $PhPMe_2$.

V.2 NMR Titration Experiment by Evans Method

In a nitrogen-filled glovebox, 15 mg (dcype)Fe(CH_3)$_2$ (**Fe1**, 0.030 mmol, 1 equiv) was dissolved in 1140 μL of benzene-d_6 solvent and distributed evenly to three oven-dried J. Young tubes containing benzene-d_6 capillaries. In a separate vial, 124 mg of PhPMe$_2$ (0.90 mmol, 30 equiv) was dissolved in 900 μL of benzene-d_6 as a stock solution. To each J. Young tube, 50 μL of the PhPMe$_2$ stock solution was added each time, followed by characterization by ^{1}H NMR spectroscopy (**Figure S16 and 17**). This step was repeated until 30 equivalents of PhPMe$_2$ was added to each J. Young tube.

Plotting the volume-corrected peak separation (V·Δv/v) against the concentration of PhPMe$_2$ led to a linear fit (**Figure S18**), consistent with coordination of one equivalent of PhPMe$_2$ to one equivalent of complex **Fe1** to generate the lower-spin, five-coordinate **Fe3**.

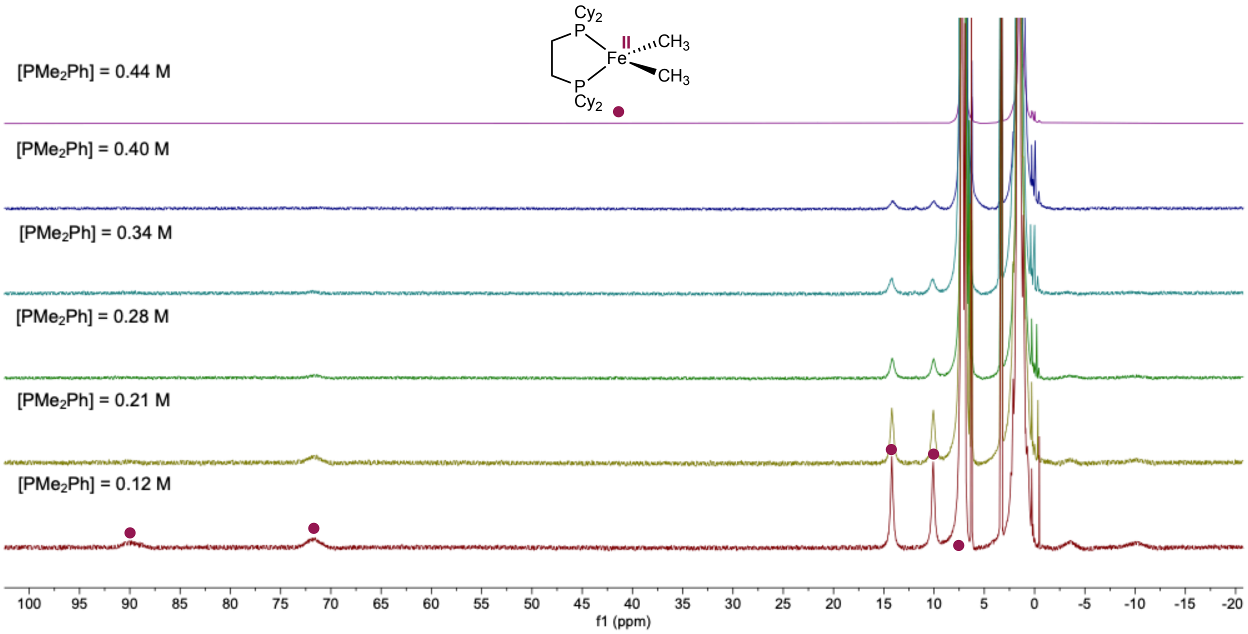

Figure S16. Representative stacked ^{1}H NMR spectra (500 MHz, benzene-d_6, 25 °C, 100 to -20 ppm) of the mixture of **Fe1** in the presence of varied concentrations of PhPMe$_2$.

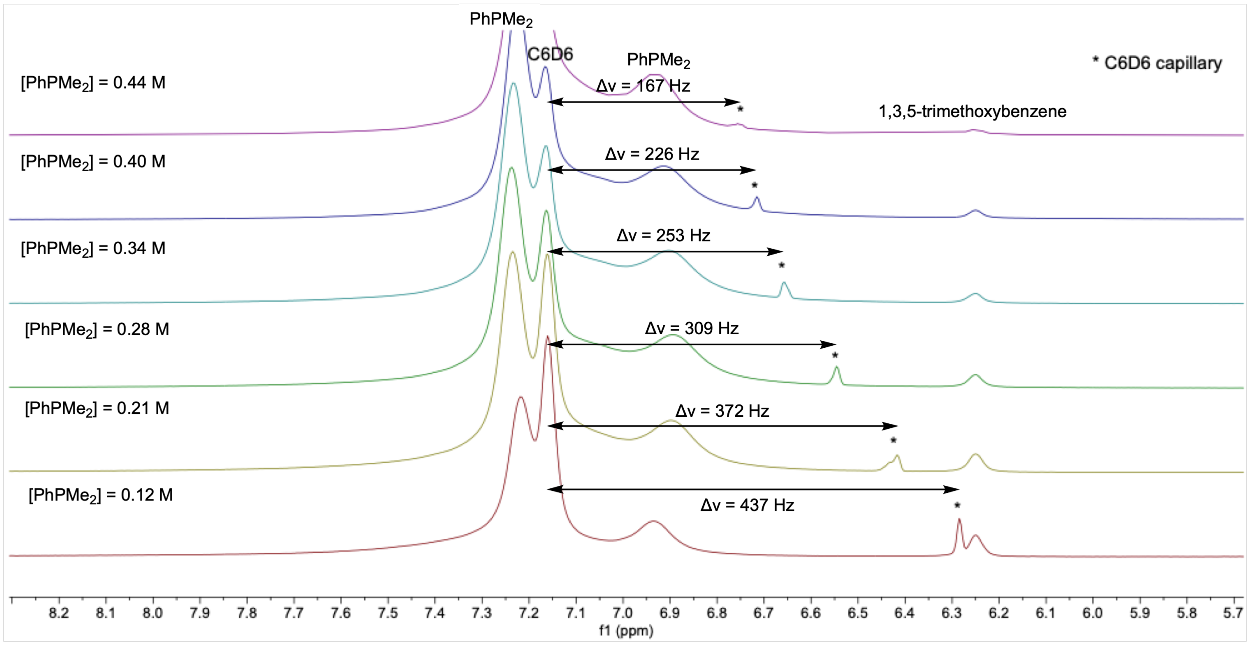

Figure S17. Representative stacked ^{1}H NMR spectra (500 MHz, benzene-d_6, 25 °C, 8.3 to 5.7 ppm) of the mixture of **Fe1** and varied concentrations of $PhPMe_2$, showing the peak separation between benzene-d_6 in bulk solution and the benzene-d_6 capillary with the increase in [$PhPMe_2$].

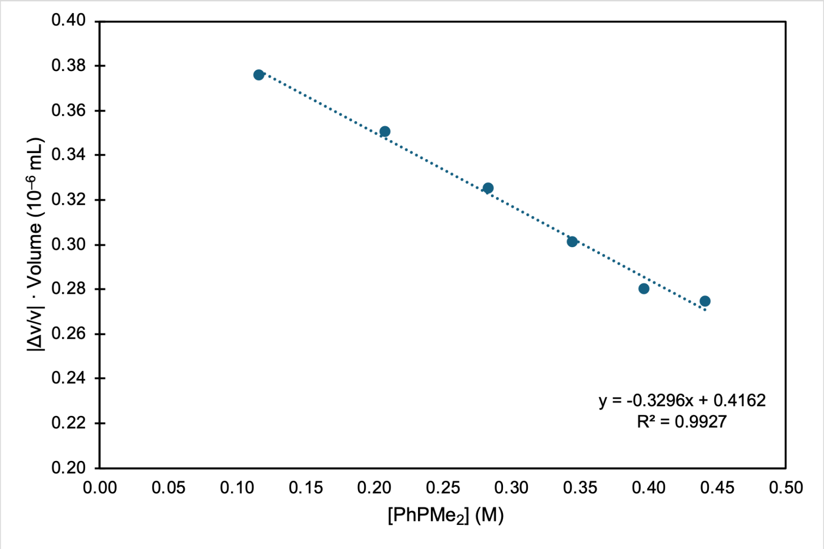

Figure S18. Linear correlation between volume-corrected peak separation (V·Δv/v) and concentration of added $PhPMe_2$.

V.3 Chiral Bis(Phosphine) Iron(II) Complexes as Stereochemical Probe

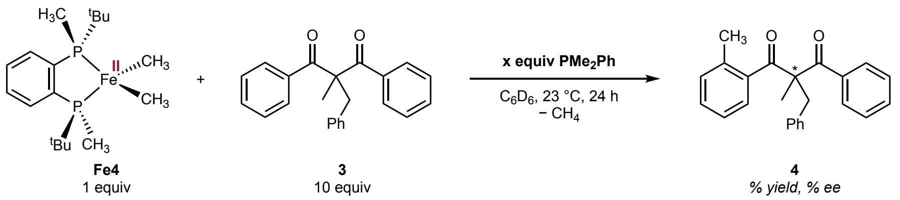

x	Yield (%)	ee (%)
**10**	47	62
**5**	41	60
**2**	41	62

Following the General Procedure using 7.4 mg of Fe4 (0.020 mmol, 1 equiv), 66 mg of arene **3** (0.20 mmol, 10 equiv) and 10 or 5 or 2 equivalents of $PhPMe_2$ (added separately as 400 μL, 200 μL, 80 μL of a benzene stock solution made from 48 mg of $PhPMe_2$ and 680 μL benzene), each reaction was stirred in a 1-dram vial charged with a magnetic stir bar at room temperature for 24 hours. Upon evaporation to dryness under air flow outside the glovebox, 0.33 equivalents of TMB were added as a stock solution in $CDCl_3$ (12 mg TMB in 1070 μL $CDCl_3$) and NMR yield of **4** was determined by quantitative ^{1}H NMR spectroscopy. Preparative TLC using EtOAc/hexanes (1:3) as the eluent allowed **4** to be isolated as a colorless oil for ee determination using chiral SFC.

Characterization Data for Compound **4**:

^{1}H NMR (500 MHz, $CDCl_3$, 25 °C): δ 7.99 – 7.88 (m, 2H), 7.54 – 7.47 (m, 1H), 7.40 (td, J = 7.8, 1.6 Hz, 2H), 7.30 – 7.20 (m, 3H), 7.17 (dd, J = 4.4, 2.5 Hz, 3H), 7.03 (t, J = 7.5 Hz, 1H), 6.91 – 6.85 (m, 2H), 3.60 (d, J = 14.0 Hz, 1H), 3.53 (d, J = 14.0 Hz, 1H), 2.42 (s, 3H), 1.49 (d, J = 1.4 Hz, 3H).

^{13}C{^{1}H} NMR (126 MHz, $CDCl_3$, 25 °C): δ 203.0, 198.7, 139.4, 137.1, 136.8, 136.4, 133.0, 132.4, 131.2, 130.8, 129.4, 128.8, 128.1, 127.4, 126.9, 125.5, 66.0, 43.0, 22.3, 21.7.

HRMS (ESI+) *m/z* calculated for $[C_{24}H_{23}O_2]^+$ ($[M+H]^+$) 343.1693, found *m/z* 343.1694.

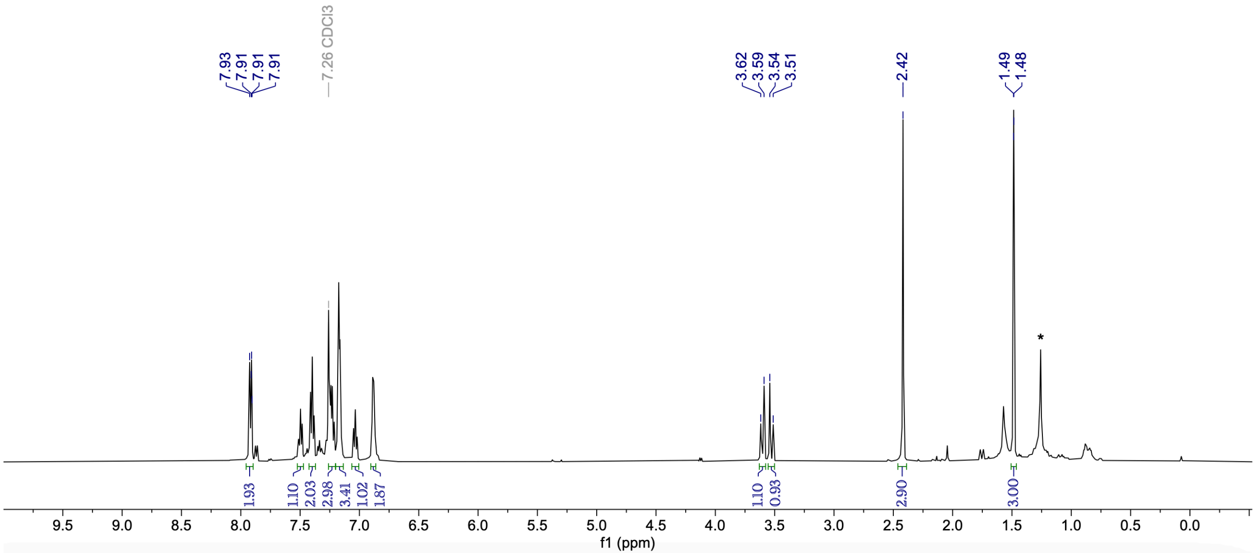

Figure S19. ^{1}H NMR (500 MHz, $CDCl_3$, 23 °C) spectrum of compound **4** after purification by preparative TLC.

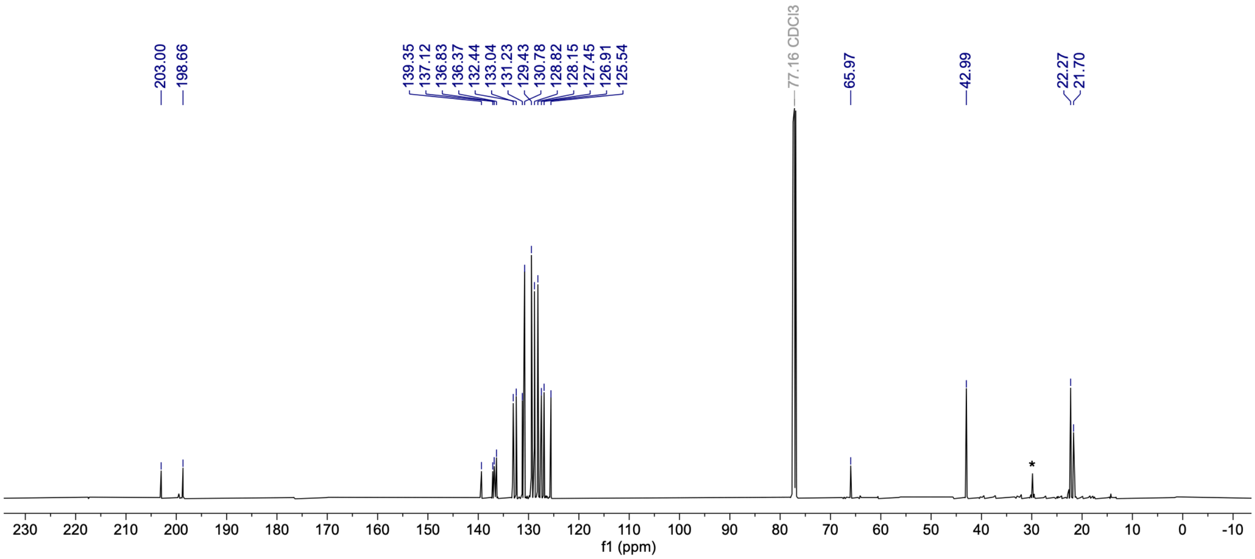

Figure S20. $^{13}C\{^{1}H\}$ NMR (126 MHz, $CDCl_3$, 25 °C) spectrum of compound **4** after purification by preparative TLC.

(a)

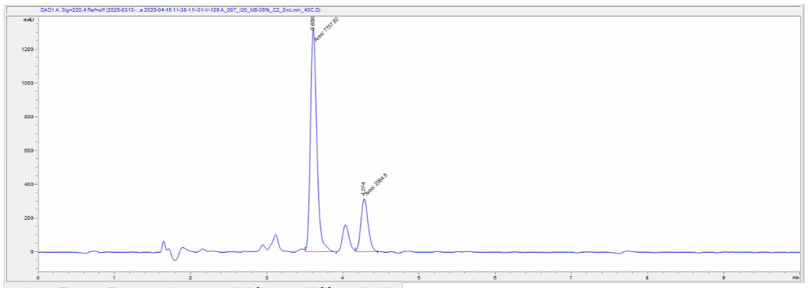

#	Time	Type	Area	Height	Width	Area%
1	3.606	MM	7757.8	1328.3	0.0973	78.821
2	4.274	MM	2084.5	316.3	0.1098	21.179

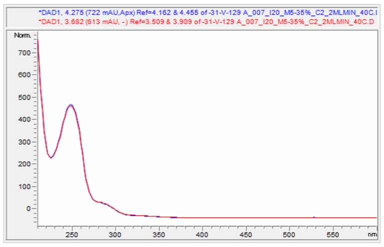

(b)

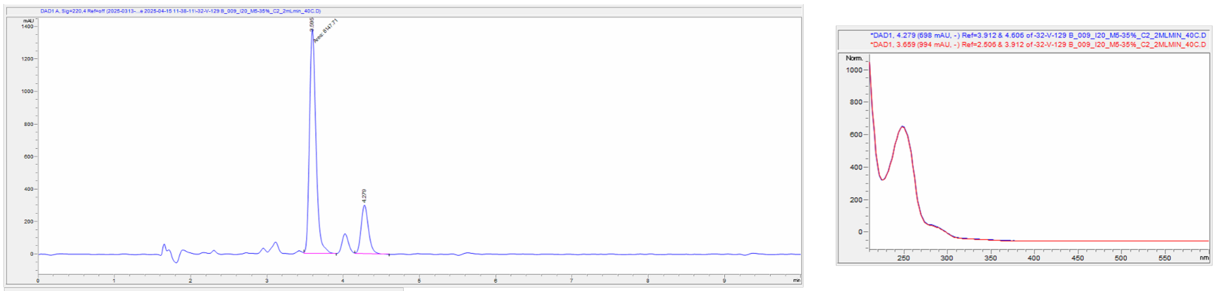

#	Time	Type	Area	Height	Width	Area%
1	3.595	FM	8147.7	1384.3	0.0981	80.109
2	4.279	VB	2023.1	300.5	0.1045	19.891

(c)

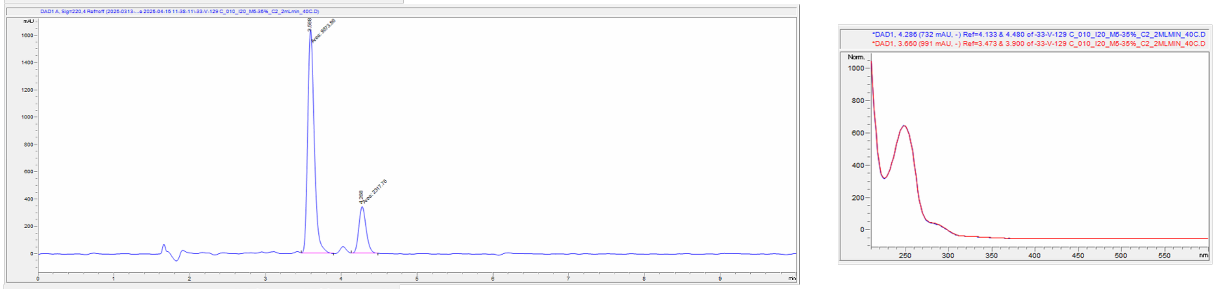

#	Time	Type	Area	Height	Width	Area%
1	3.588	MM	9873.9	1640	0.1003	80.989
2	4.268	MM	2317.8	343.7	0.1124	19.011

Figure S21. Chiral SFC chromatogram for determination of enantiomeric excess of product **4** obtained from stoichiometric reaction between arene **3** and (*R*,*R*)-BenzP*-supported complex **Fe4** in the presence of (a) 10 equivalents, (b) 5 equivalents, and (c) 2 equivalents of exogenous $PhPMe_2$.

Chiral bidentate phosphines other than (*R*,*R*)-BenzP* as the ancillary ligand were also competent stereochemical probes (**Figure S22 and 23**). A modified version of the general procedure was used, where the bis(phosphine) iron dimethyl was generated in situ – by stirring the corresponding chiral bis(phosphine)-ligated iron dichloride **(P^P*)$FeCl_2$** and stoichiometric amount of $(TMEDA)Mg(CH_3)_2$ in benzene at room temperature for 30 minutes – and used directly after filtration through a pad of celite. These results provided further evidence that the bis(phosphine) ligand remains coordinated to the iron center throughout the C–H methylation reaction.

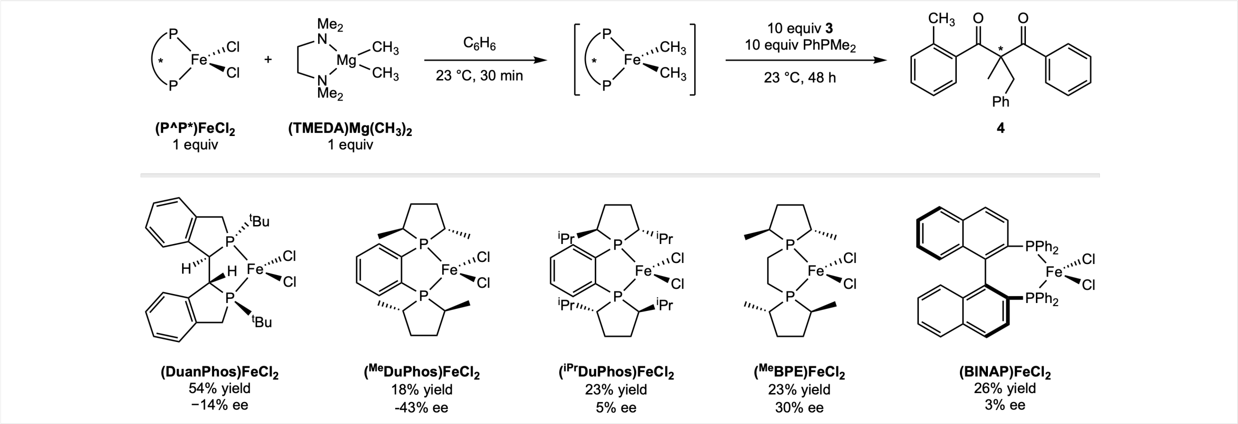

Figure S22. Other chiral bis(phosphine) ligands as stereochemical probes.

(a)

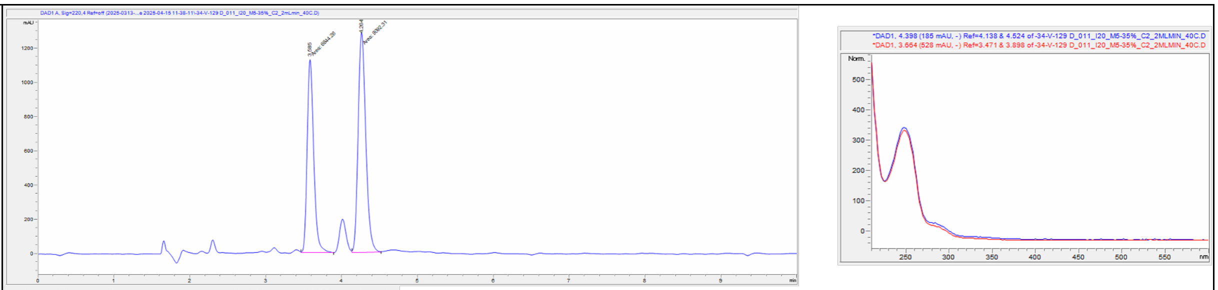

#	Time	Type	Area	Height	Width	Area%
1	3.585	MM	6844.3	1134.4	0.1006	42.947
2	4.264	MM	9092.3	1291.7	0.1173	57.053

(b)

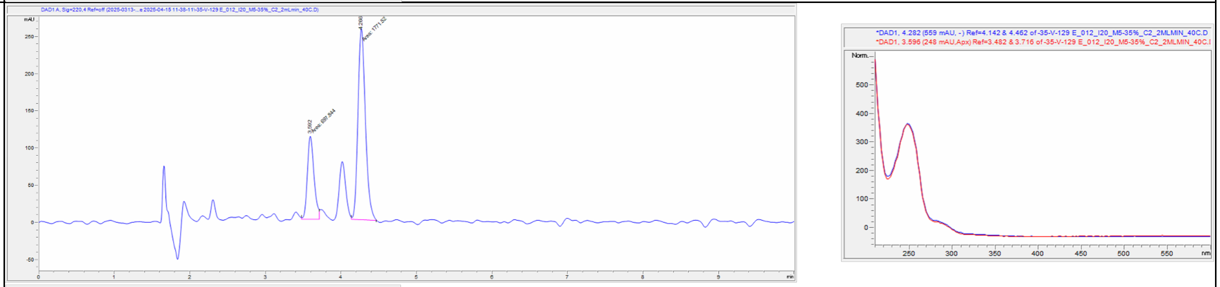

#	Time	Type	Area	Height	Width	Area%
1	3.592	MM	697.8	112.5	0.1034	28.260
2	4.266	MM	1771.5	258.2	0.1144	71.740

(c)

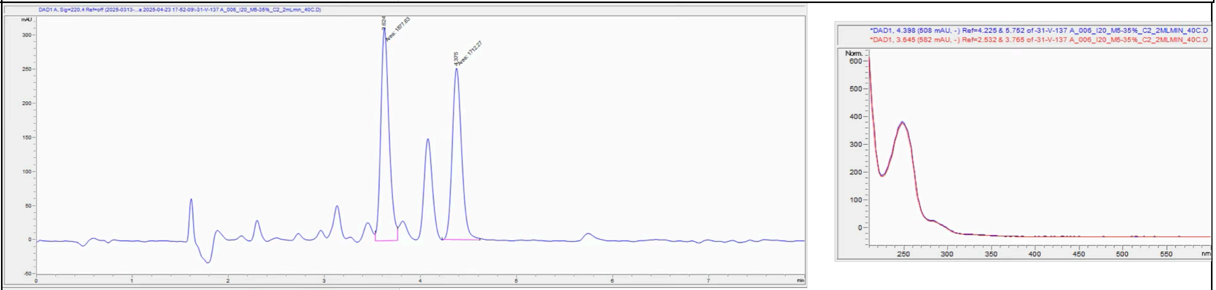

#	Time	Type	Area	Height	Width	Area%
1	3.624	FM	1877.6	312.2	0.1002	52.303
2	4.375	MF	1712.3	252.1	0.1132	47.697

(d)

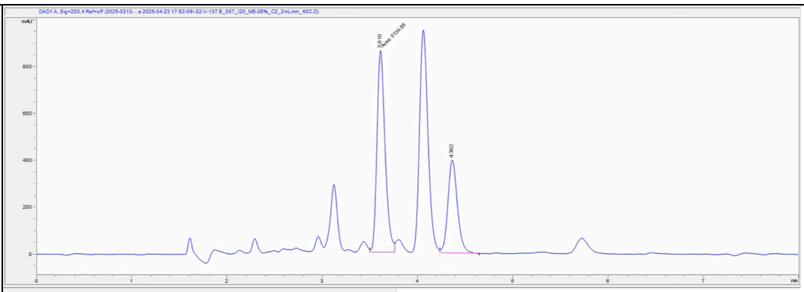

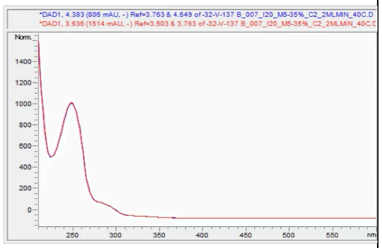

#	Time	Type	Area	Height	Width	Area%
1	3.61	MM	5134.9	863	0.0992	64.995
2	4.362	VB	2765.5	397	0.1073	35.005

(e)

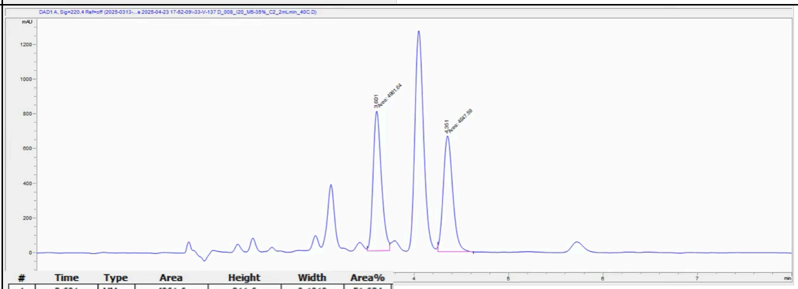

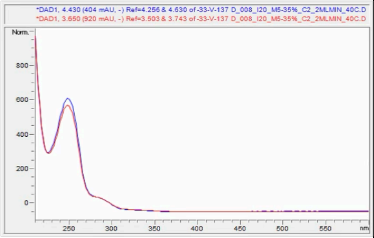

#	Time	Type	Area	Height	Width	Area%
1	3.601	MM	4961.6	811.6	0.1019	51.634
2	4.351	MM	4647.6	670.3	0.1156	48.366

Figure S23. Chiral SFC chromatogram for determination of enantiomeric excess of product **4** obtained from stoichiometric reaction between arene **3** and an in situ generated chiral bis(phosphine)-supported iron(II) dimethyl complex in the presence of 10 equivalents of exogenous $PhPMe_2$, where the chiral bis(phosphine) is (a) (1*R*,1'*R*,2*S*,2'*S*)-DuanPhos; (b) (*R*,*R*)-Me-DuPhos; (c) (*R*,*R*)-iPr-DuPhos; (d) (*R*,*R*)-Me-BPE; (e) (*R*)-(+)-BINAP.

VI. Kinetic Data

VI.1 Kinetic Distinction between Pathway 1 and Pathway 2

Rate law derivation was performed under steady-state approximation using the "rule of thumb" technique outlined by Gilbert,[29] with C−H cleavage as the rate-determining step (RDS).

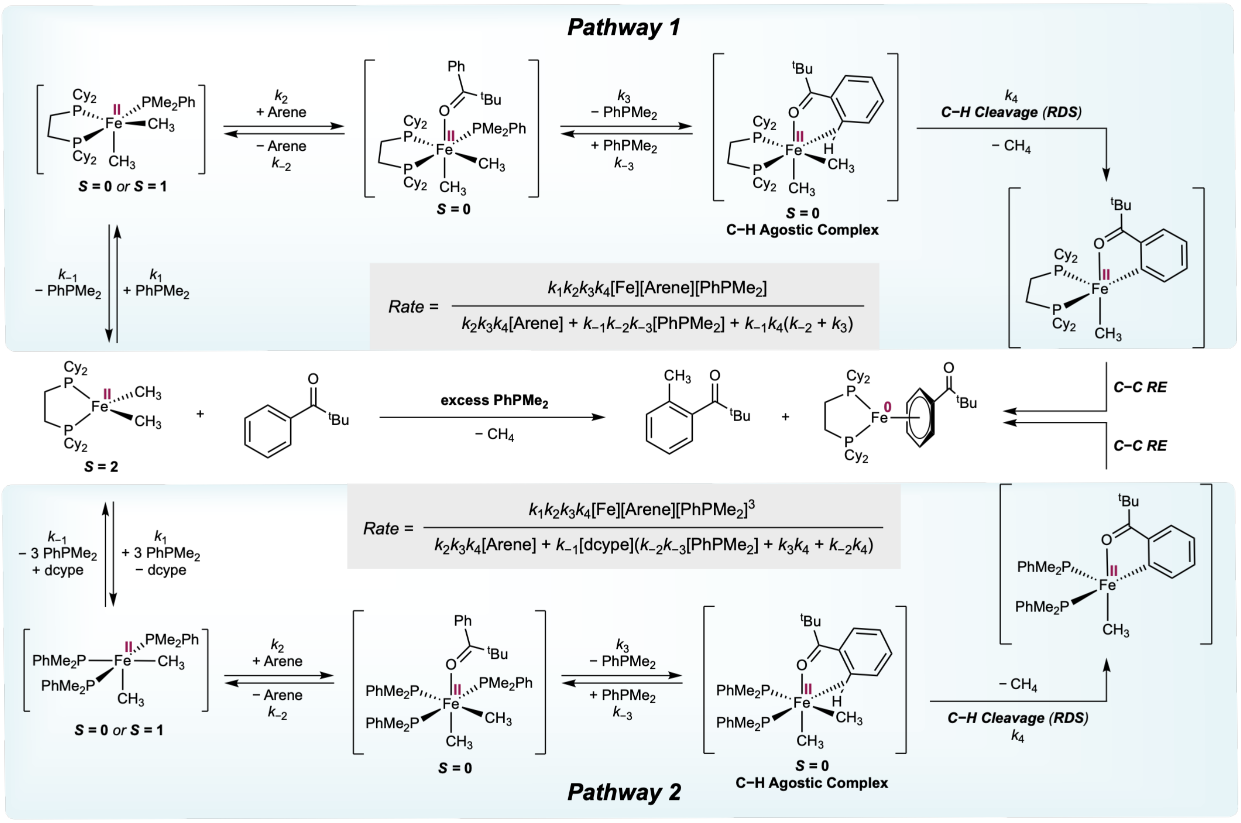

VI.2 Determination of Empirical Rate Law

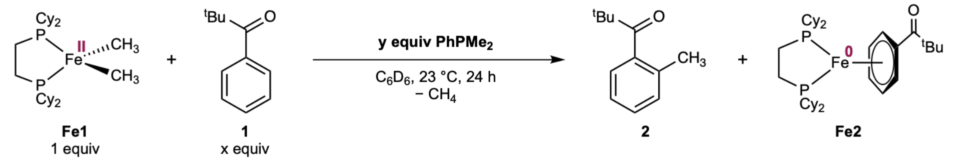

General Procedure for ^{1}H NMR Kinetic Studies: In a N_2-filled glovebox, 1 equivalent of **Fe1** (320 µL, prepared as a 0.12 M stock solution in benzene-d_6) was added to a J. Young tube containing x equivalents of arene **1**, y equivalents of $PhPMe_2$, 0.33 equivalents of TMB internal standard, and 180 µL of benzene-d_6 solvent in a J. Young NMR tube. If applicable, additional benzene-d_6 was added to maintain a constant total volume of 500 µL for all samples. Note that the mixture of arene **1**, $PhPMe_2$, and TMB was prepared as respective stock solutions in pentane, mixed and concentrated *in vacuo* in a 1-dram vial, before being dissolved in benzene-d_6 and transferred to a J. Young tube.

The reaction progress was subsequently monitored by the concentration of complex **Fe2** formed using quantitative ^{1}H NMR spectroscopy at 23 °C. The results were analyzed using the visual time normalization analysis (VTNA) method developed by Burés and co-workers.[30,31,32]

Evaluation of order dependence on [$PhPMe_2$]: Reactions were performed according to General Procedure with 49 mg of arene **1** (0.30 mmol, 10 equiv), 15 mg of complex **Fe1** (0.030 mmol, 1 equiv), and 5.0 mg of TMB internal standard (0.030 mmol, 1 equiv) evenly distributed to three J. Young tubes. The only modification was that 21 mg of $PhPMe_2$ (0.015 mmol) was dissolved in 1.5 mL pentane and the volume of pentane stock solution of $PhPMe_2$ added to each sample was varied, as follows:

- [$PhPMe_2$] = 40 mM: 200 µL of $PhPMe_2$ stock solution in pentane
- [$PhPMe_2$] = 60 mM: 400 µL of $PhPMe_2$ stock solution in pentane
- [$PhPMe_2$] = 80 mM: 800 µL of $PhPMe_2$ stock solution in pentane

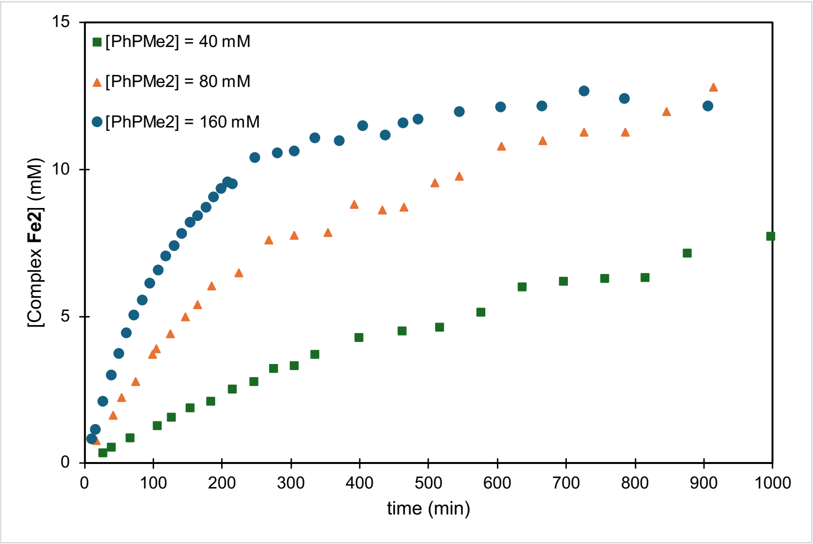

Figure S24. Time course of formation of complex **Fe2** with varying initial concentrations of $PhPMe_2$.

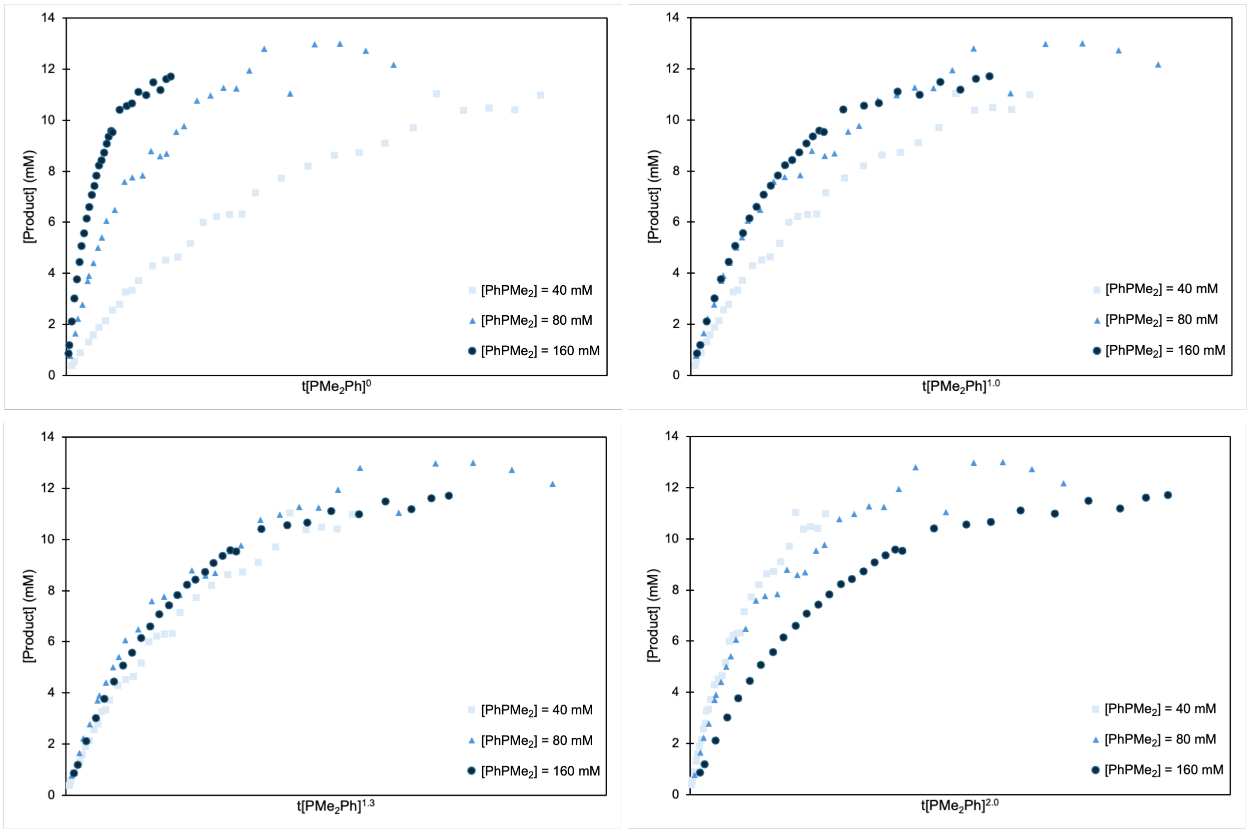

Figure S25. Determination of approximate first-order dependence on $PhPMe_2$ concentration by VTNA overlay.

Evaluation of order dependence on [arene 1]: Reactions were performed according to General Procedure with 11 mg of $PhPMe_2$ (0.080 mmol, 2 equiv), 20 mg of complex **Fe1** (0.040 mmol, 1

equiv), and 6.7 mg of TMB internal standard (0.040 mmol, 1 equiv) evenly distributed to three J. Young tubes – 200 µL stock solution per tube. 74 mg of arene **1** (0.46 mmol) was then dissolved in 900 µL benzene-d_6 and the volume of benzene-d_6 stock solution of arene **1**, as well as the volume of benzene-d_6 to ensure a constant total volume of 500 µL, was varied as follows:

- [arene **1**] = 0.15 M: 150 µL of **1** stock solution in benzene-d_6 + 150 µL benzene-d_6
- [arene **1**] = 0.20 M: 200 µL of **1** stock solution in benzene-d_6 + 100 µL benzene-d_6
- [arene **1**] = 0.25 M: 250 µL of **1** stock solution in benzene-d_6 + 50 µL benzene-d_6
- [arene **1**] = 0.30 M: 300 µL of **1** stock solution in benzene-d_6

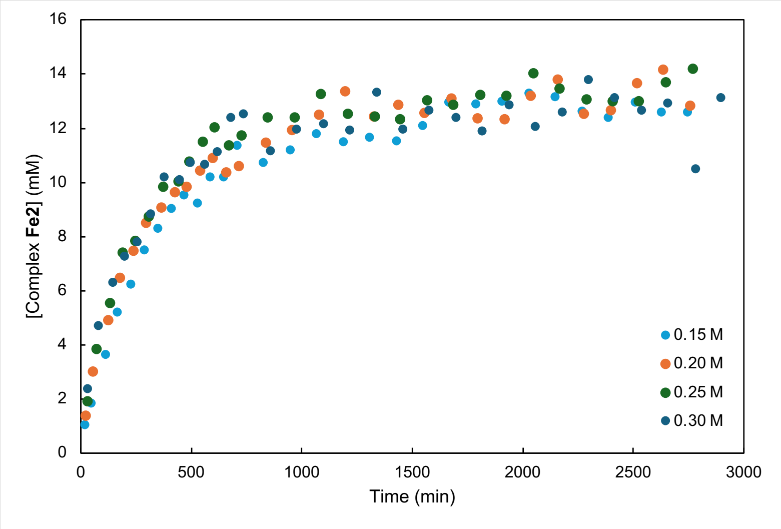

Figure S26. Time course of the formation of complex **Fe2** with varying initial concentrations of arene **1**. Initial concentration of **1** for each run was specified in the legend.

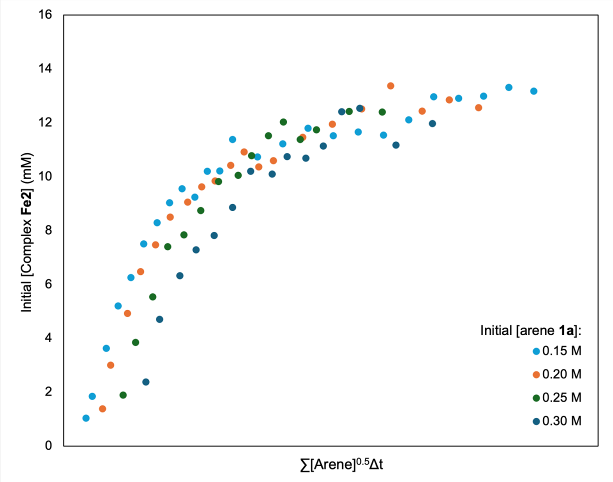

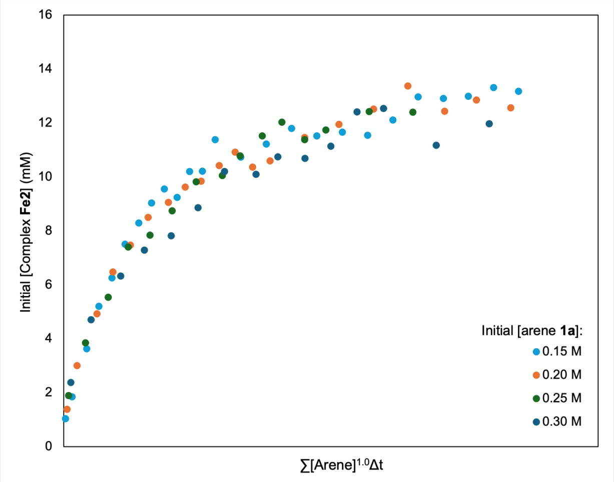

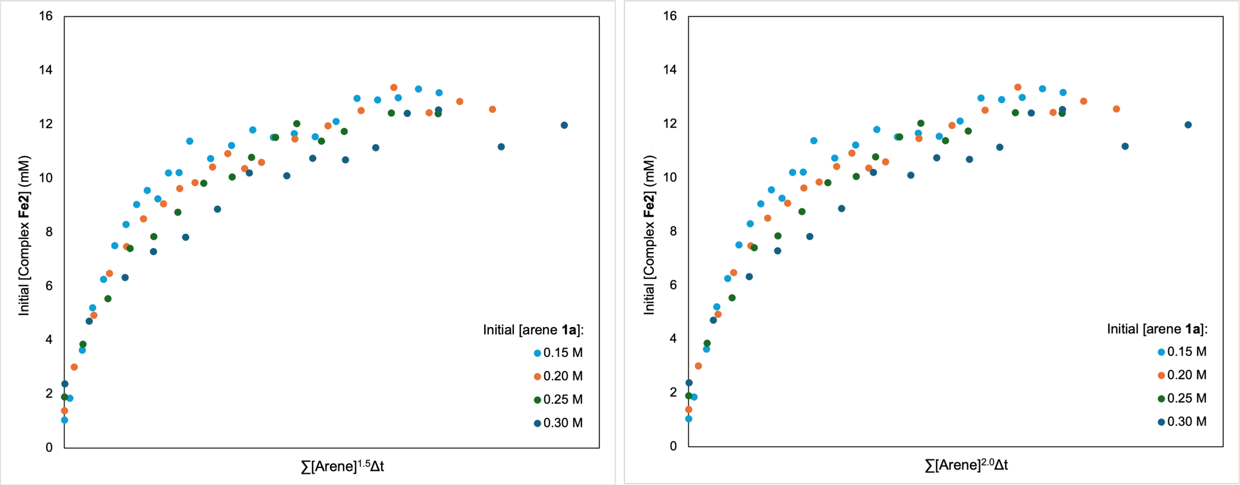

Figure S27. Determination of first-order dependence on arene **1** concentration by VTNA overlay.

Evaluation of order dependence on [Fe1]: Reactions were performed according to General Procedure with 12 mg of $PhPMe_2$ (0.087 mmol, 2.2 equiv), 67 mg of arene **1** (0.041 mmol, 10 equiv), and 8 mg of TMB internal standard (0.048 mmol, 1.2 equiv) evenly distributed to three J. Young tubes – as a 150 µL stock solution per tube. 18 mg of complex **Fe1** (0.035 mmol) was then dissolved in 1000 µL benzene-d_6. The volume of benzene-d_6 stock solution of complex **Fe1**, as well as the volume of benzene-d_6 to ensure a constant total volume of 500 µL, was varied as follows:

- [**Fe1**] = 10 mM: 140 µL of **Fe1** stock solution in benzene-d_6 + 210 µL benzene-d_6
- [**Fe1**] = 15 mM: 210 µL of **Fe1** stock solution in benzene-d_6 + 140 µL benzene-d_6
- [**Fe1**] = 20 mM: 280 µL of **Fe1** stock solution in benzene-d_6 + 70 µL benzene-d_6
- [**Fe1**] = 25 mM: 350 µL of **Fe1** stock solution in benzene-d_6

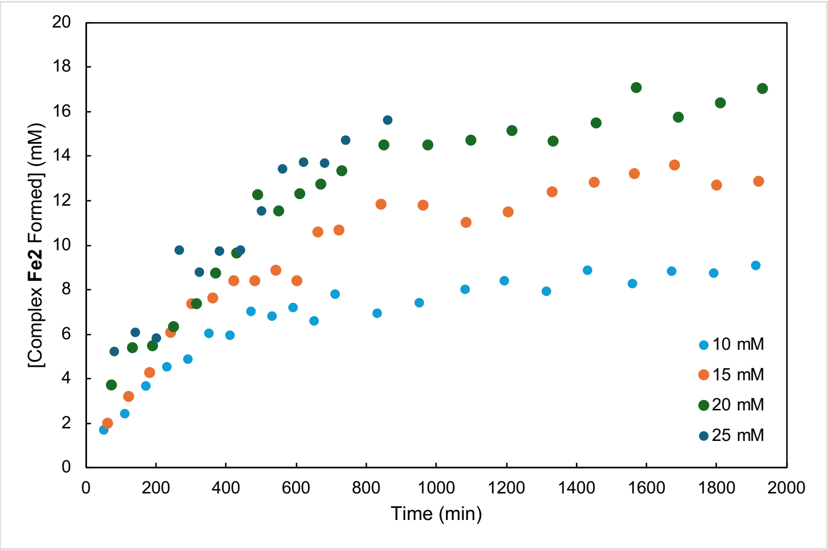

Figure S28. Time course of the formation of complex **Fe2** with varying initial concentrations of **Fe1**. Initial concentration of **Fe1** for each run was specified in the legend.

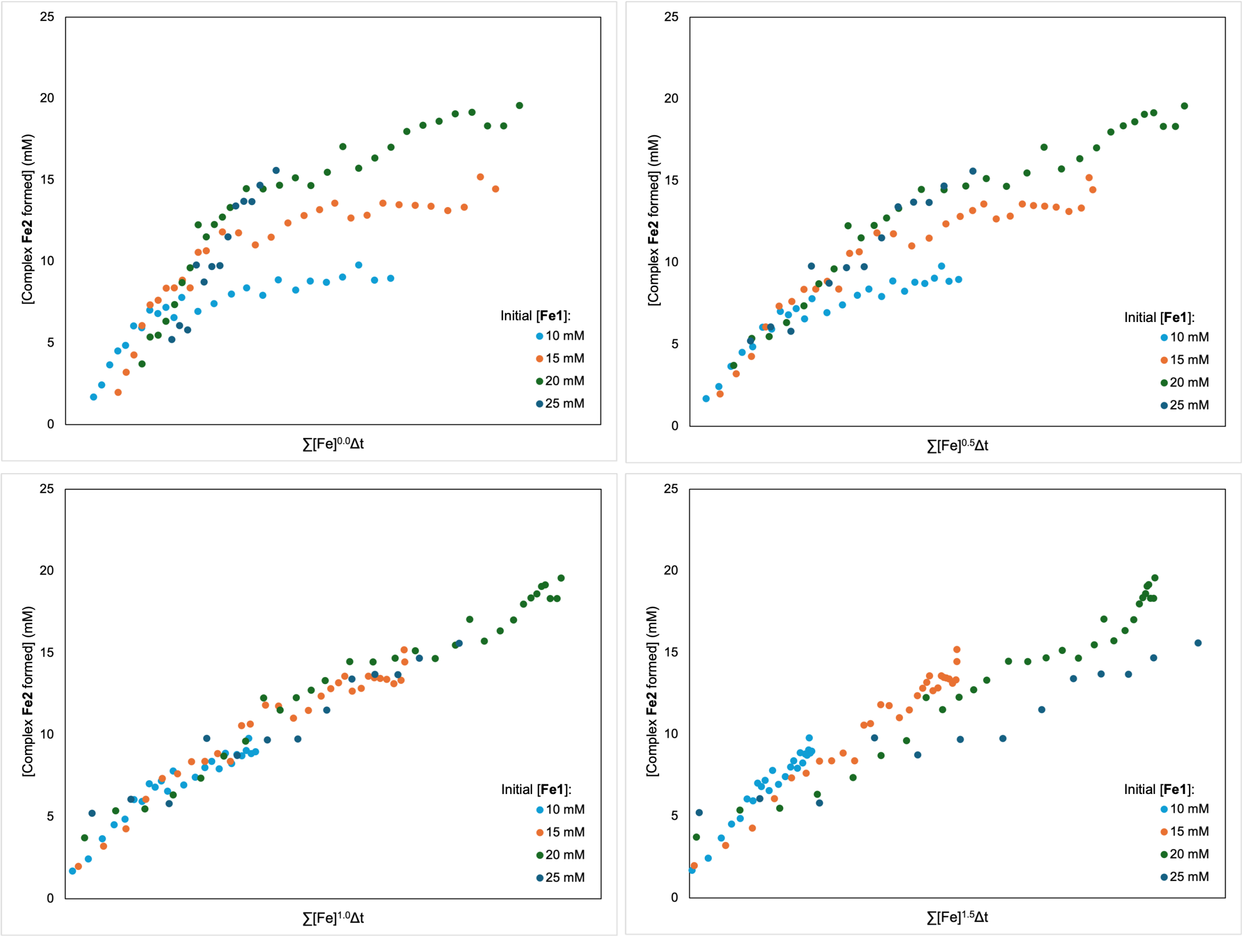

Figure S29. Determination of first-order dependence on the concentration of complex **Fe1** by VTNA overlay.

VI.3 Parallel Deuterium Kinetic Isotope Effect Experiments

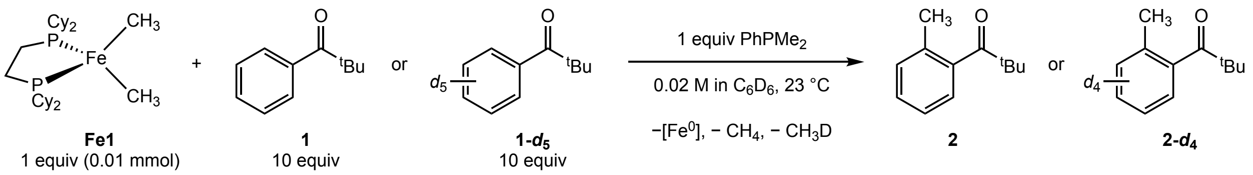

In two sets of two separate J. Young tubes in a N_2-filled glovebox, the reaction was set up with the natural abundance arene (**1**) or the deuterated arene (**1-*d*$_5$**) based on the General Procedure described above. The amount of functionalized arene (**2** or **2-*d*$_4$**) formed was analyzed by quantitative ^{1}H NMR with respect to the TMB internal standard. Product concentration was plotted against time lapsed from 0 to 10% NMR yield of functionalized arene (**Figure S30**). The reaction with natural abundance versus the deuterated arene was compared and the value of KIE was determined from the average value of the slope of the two plots (KIE: k_H / k_D = 4.4(3)).

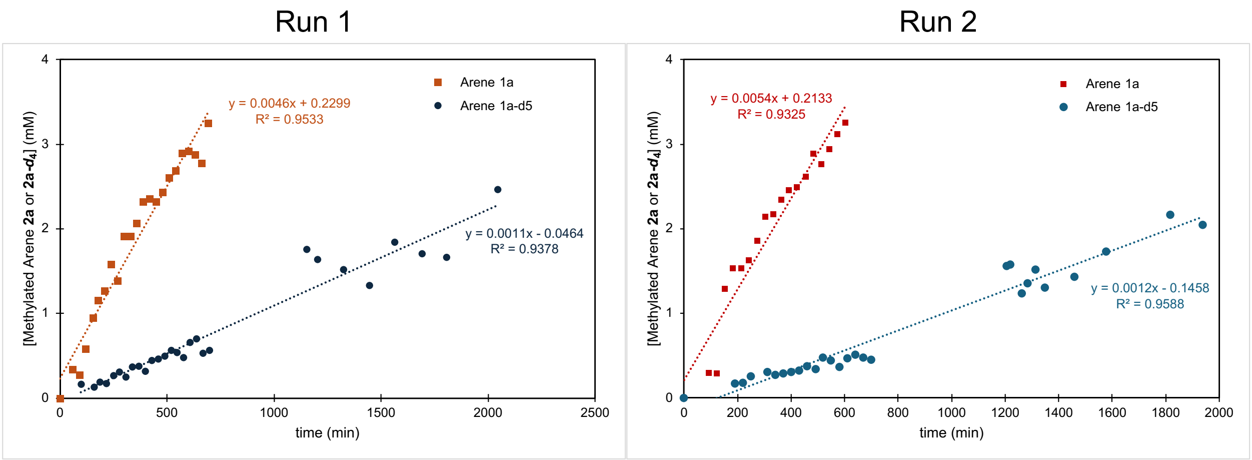

Figure S30. Plot of concentration of *ortho*-methylated arene **2** or **2-*d*$_4$** against time lapsed for two separate runs of parallel deuterium KIE experiments.

VII. X-Ray Crystallographic Data

Table S1. Crystal data for complex **Fe1** (CCDC 2505728).

Crystal data	
Chemical formula	2($C_{28}H_{54}FeP_2$)
M_r	1017.00
Crystal system, space group	Orthorhombic, $Pmn2_1$
Temperature (K)	100
a, *b*, *c* (Å)	22.294 (3), 15.4450 (18), 8.3791 (10)
V (Å^3)	2885.2 (6)
Z	2
Radiation type	Cu *K*α
μ (mm^{-1})	5.31
Crystal size (mm)	0.29 × 0.14 × 0.10
Data collection	
Diffractometer	XtaLAB Synergy, Dualflex, HyPix-Arc 150
Absorption correction	Multi-scan<br>*CrysAlis PRO* 1.171.44.128a (Rigaku Oxford Diffraction, 2025) Empirical absorption correction using spherical harmonics, implemented in SCALE3 ABSPACK scaling algorithm.
T_{min}, T_{max}	0.576, 1.000
No. of measured, independent and observed [$I > 2\sigma(I)$] reflections	23021, 5580, 2735
R_{int}	0.167
(sin θ/λ)$_{max}$ (Å^{-1})	0.626
Refinement	
$R[F^2 > 2\sigma(F^2)]$, $wR(F^2)$, S	0.150, 0.421, 1.24
No. of reflections	5580
No. of parameters	146
No. of restraints	10
H-atom treatment	H-atom parameters constrained
$\Delta\rangle_{max}$, $\Delta\rangle_{min}$ (e Å^{-3})	0.81, -1.00
Absolute structure	Refined as an inversion twin.
Absolute structure parameter	0.33 (3)

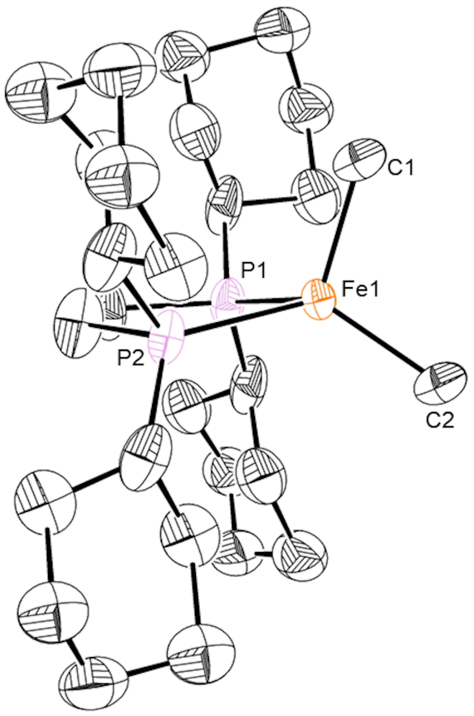

Figure S31. Representation of the solid-state structure of (dcype)Fe(CH_3)$_2$ (**Fe1**) at 30% probability ellipsoids, as determined from SC-XRD. H atoms are omitted for clarity. CCDC 2505728.

Table S2. Crystal data for complex **Fe4** (CCDC 2515364).

Crystal data	
Chemical formula	2($C_{18}H_{34}FeP_2$)
M_r	736.48
Crystal system, space group	Monoclinic, $P2_1$
Temperature (K)	100
a, *b*, *c* (Å)	12.8356 (13), 9.6214 (7), 17.6559 (18)
β (°)	106.371 (10)
V (Å^3)	2092.0 (4)
Z	2
Radiation type	Cu *K*α
μ (mm^{-1})	7.15
Crystal size (mm)	0.32 × 0.08 × 0.07
Data collection	
Diffractometer	XtaLAB Synergy, Dualflex, HyPix-Arc 150
Absorption correction	Multi-scan<br>*CrysAlis PRO* 1.171.44.128a (Rigaku Oxford

	Diffraction, 2025) Empirical absorption correction using spherical harmonics, implemented in SCALE3 ABSPACK scaling algorithm.
T_{min}, T_{max}	0.829, 1.000
No. of measured, independent and observed [$I > 2\sigma(I)$] reflections	40011, 7320, 4045
R_{int}	0.117
$(\sin \theta/\lambda)_{max}$ ($Å^{-1}$)	0.630
Refinement	
$R[F^2 > 2\sigma(F^2)]$, $wR(F^2)$, S	0.106, 0.338, 1.11
No. of reflections	7320
No. of parameters	399
No. of restraints	1
H-atom treatment	H-atom parameters constrained
$\Delta\rangle_{max}$, $\Delta\rangle_{min}$ (e $Å^{-3}$)	0.82, -1.52
Absolute structure	Flack x determined using 968 quotients [(I+)-(I-)]/[(I+)+(I-)] (Parsons, Flack and Wagner, Acta Cryst. B69 (2013) 249-259).
Absolute structure parameter	-0.025 (9)

VIII. Computational Analyses

In the DFT-computed structures of the intermediate **Int1** formed from coordination of pivalophenone **1** to the five-coordinate complex **Fe3**, the singlet state ^{1}**[Int1]** was distinguished by the evident formation of a CH-π interaction between the pivalophenone C(sp^2)−H bond and the π-system of the $PhPMe_2$ phenyl ring, which contributes to a less thermodynamically uphill formation of the five-coordinate (dcype)(PR_3)Fe(CH_3)$_2$ intermediate for PR_3 = $PhPMe_2$ (**Fe3**) than PR_3 = $PhMe_3$ (**Fe3-PMe$_3$**) as shown in **Figure 4a**. This is corroborated by the higher yield of C–H methylation with $PhPMe_2$ than with PMe_3 as the spin modulator.

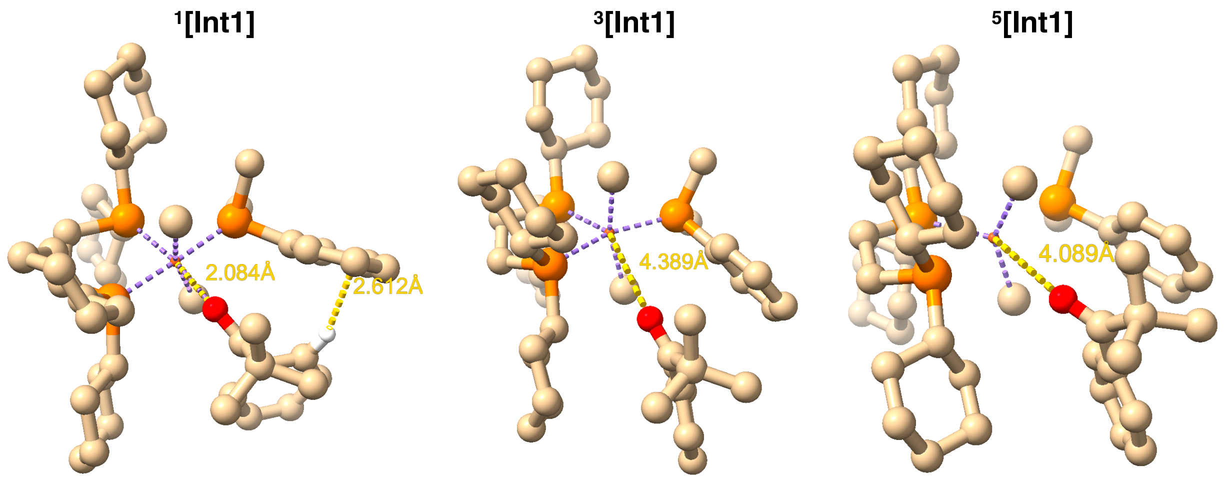

Figure S32. DFT-computed structures for singlet, triplet and quintet states of 6-coordinate intermediate **Int1** formed from coordination of arene **1** (via the ketone directing group) to the 5-coordinate complex **Fe3**. All except selected H atoms are omitted for clarity. Selected interatomic distances are labelled as yellow dashed lines. All structures were visualized with ChimeraX 1.9 software.[25]

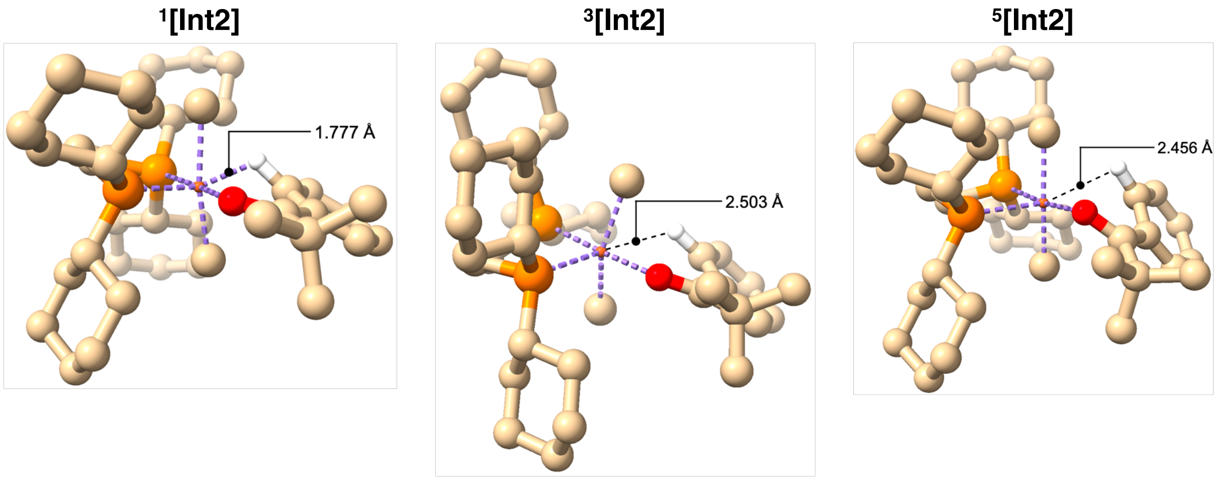

Figure S33. DFT-computed structures for singlet, triplet and quintet states of C–H σ-agostic complex **Int2**. All except selected H atoms are omitted for clarity. Selected interatomic distances are labelled. All structures were visualized with ChimeraX 1.9 software.[25]

For completeness, all possible configurations of the iron(II) intermediates and transition states involved in arene coordination, C–H activation, and C–C reductive elimination have been considered in the DFT calculations (**Figure S34**). The lowest-barrier pathway was found to be proceeding through the **Int1** → **Int2** → **TS1** → **Int3** → **Int4** → **TS2** sequence.

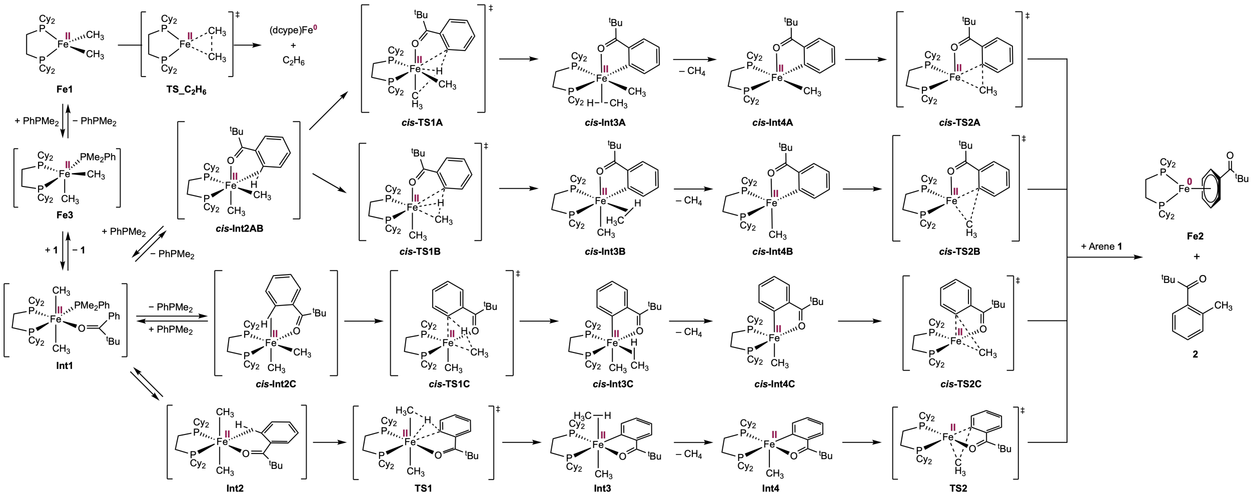

Figure S34. All probable mechanistic pathways for *ortho*-C–H methylation of pivalophenone **1** starting from **Fe1** and $PhPMe_2$ were considered in DFT computations.

The potential energy surfaces of the other three potential but higher-barrier mechanistic pathways involving isomeric iron(II) intermediates or transition states of different configurations have also been calculated (**Figure S35 to 37**).

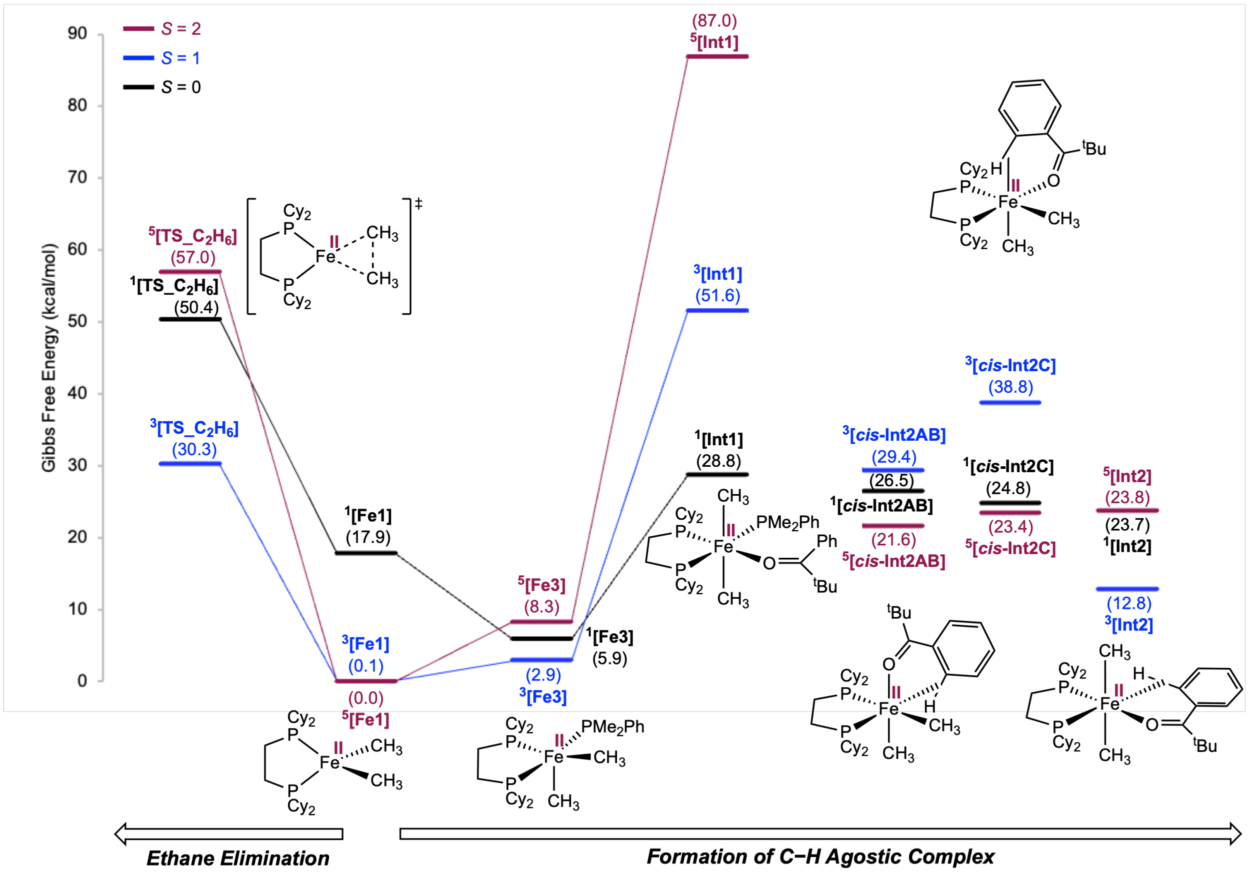

Figure S35. Calculated potential energy surface showing the comparison between the formation of C–H agostic complex (productive pathway) with the unproductive ethane elimination side reaction, based on DFT computations performed at the (U)TPSSh/def2-TZVPP/SMD(benzene)//(U)TPSS/def2-TZVP level of theory.

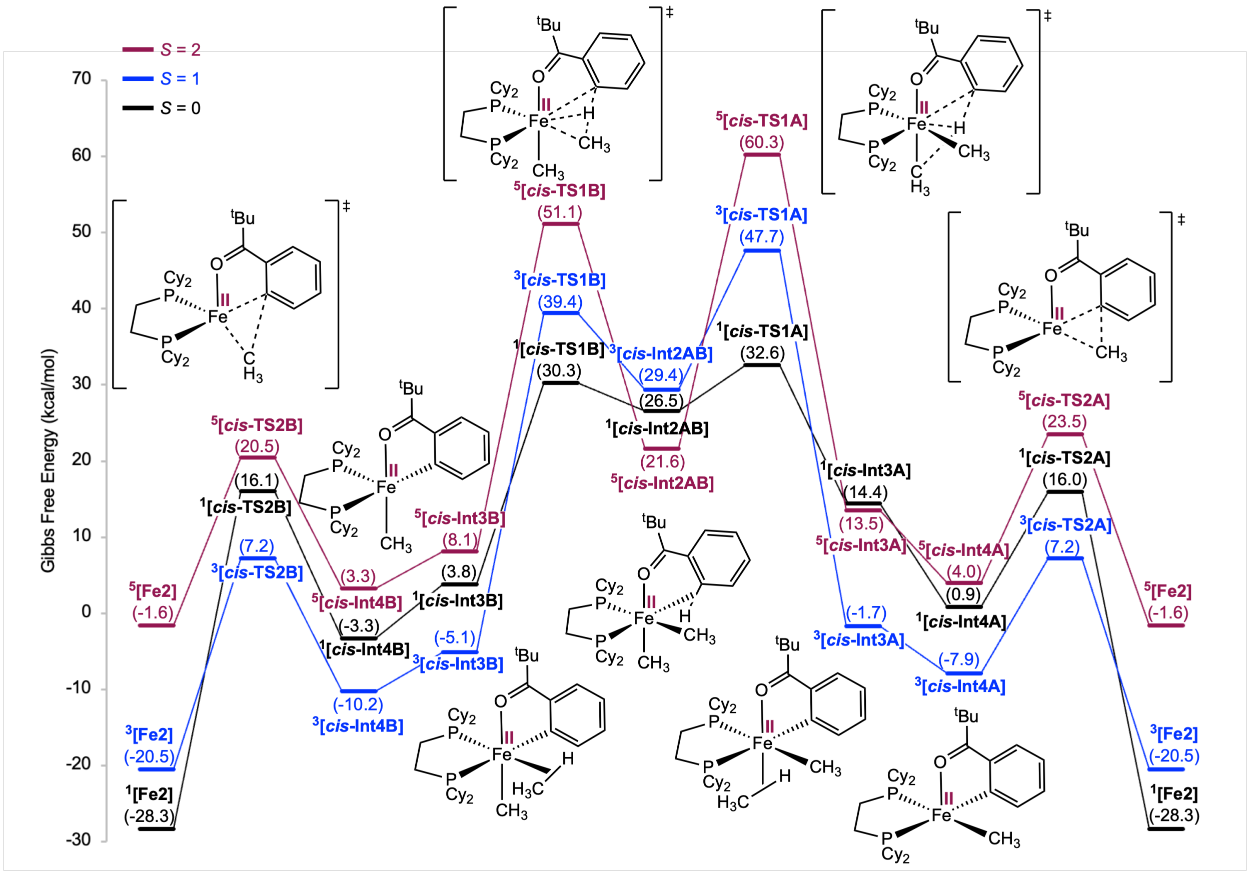

Figure S36. Calculated potential energy surface for C–H methylation of pivalophenone **1** starting from C–H agostic complex ***cis*-Int2AB**, based on DFT computations performed at the (U)TPSSh/def2-TZVPP/SMD(benzene)//(U)TPSS/def2-TZVP level of theory.

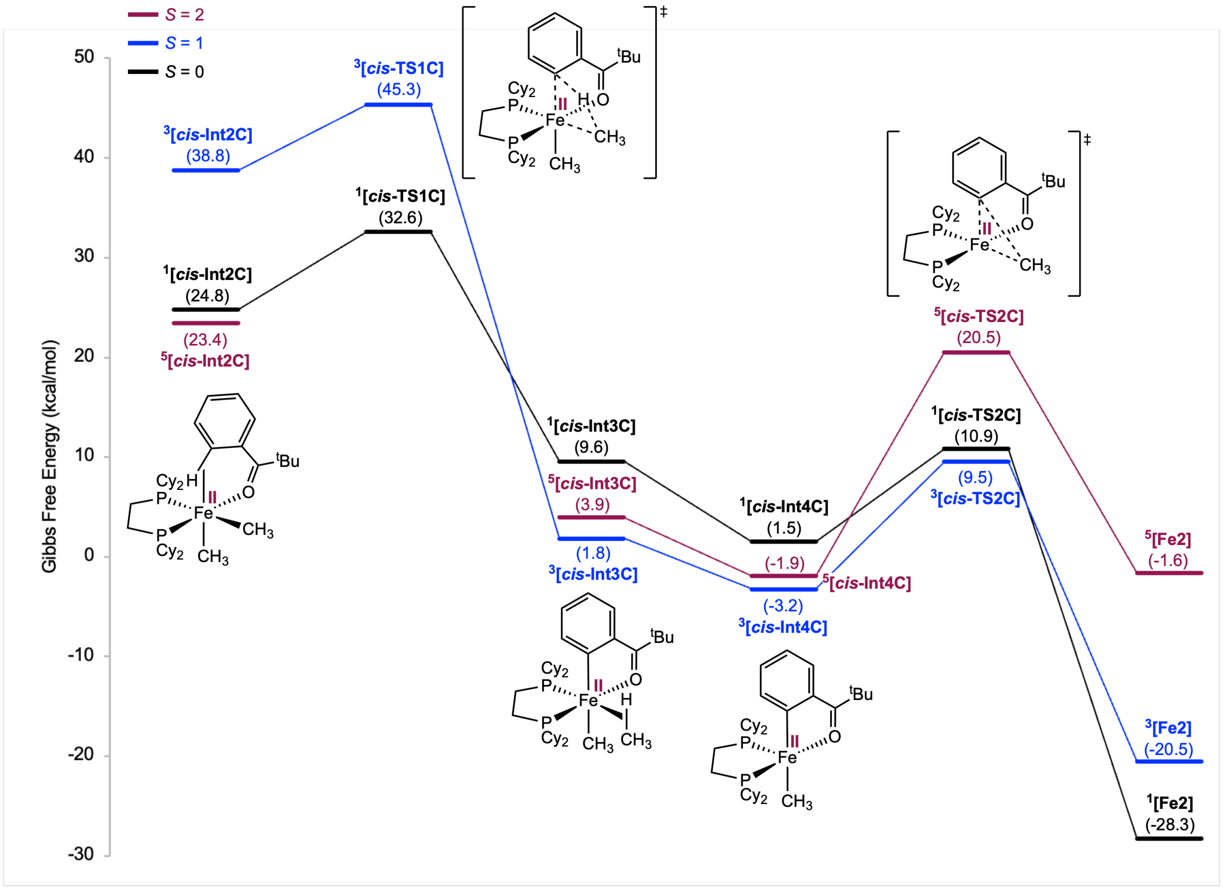

Figure S37. Calculated potential energy surface for C–H methylation of pivalophenone **1** starting from C–H agostic complex ***cis*-Int2C**, based on DFT computations performed at the (U)TPSSh/def2-TZVPP/SMD(benzene)//(U)TPSS/def2-TZVP level of theory.

The deuterium kinetic isotope effect was calculated by replacing the five aryl C–H hydrogen atoms in arene **1** with deuterium atoms (M = 2.00141) and recalculating the zero-point energies (ZPE) at 23 °C (296 K). The calculated ZPE values are tabulated below:

	ZPE (Eh)
natural abundance arene **1**	0.21851656
deuterated arene **1-*d*$_5$**	0.20240718
natural abundance ^{1}**[TS1]**	0.98223997
deuterated ^{1}**[TS1]-*d*$_5$**	0.96691012

ΔZPE(H) – ΔZPE(D) was calculated to be -0.489 kcal/mol, corresponding to k_H/k_D = 2.3.

VIII.1 Sample Geometry Optimization Input File

```
! RKS Opt TPSS D4 RIJCOSX DKH-def2-TZVP def2/J def2-TZVP/C Freq TightOpt TightSCF
Slowconv

%base " insert_base_name_for_this_job"

%SCF
        MaxIter 5000
end

* xyzfile 0 1 [insert_input_geometry_name_here].xyz

%pal nprocs 16 end

%maxcore 7000
```

VIII.2 Sample Transition State Geometry Optimization Input File

```
! RKS OptTS TPSS D4 RIJCOSX DKH-def2-TZVP def2/J def2-TZVP/C Freq TightOpt TightSCF
Slowconv

%base "insert_base_name_for_this_job"

%method
	Z_Tol 2e-4
	Z_MaxIter 500
end

%geom
	TS_Mode {B 1 2} end
	Calc_Hess true
	MaxIter 500
end

* xyzfile 0 1 [insert_input_geometry_name_here].xyz

%pal nprocs 16 end

%maxcore 8000
```

VIII.3 Sample Single Point Calculation Input File

```
! RKS SP TPSSh D4 RIJCOSX DKH-def2-TZVPP def2/J def2-TZVPP/C TightSCF CPCM

%base "enter_base_name_for_this_job"

%SCF
        MaxIter 5000
end

%cpcm
        smd true
        SMDsolvent "benzene"
end

* xyzfile 0 1 [input_geometry_name].xyz

%pal nprocs 8 end

%maxcore 2500

%output
        Print[P_Basis] 2
        Print[P_MOs] 1
end
```

VIII.4 Coordinates and Energies

The coordinates of all computed structures and intermediates are included as .xyz files in a compressed .zip file. The single point energies of the computed structures in **Figure 4** and **Figure S35 to 37** are provided in the data tables below.

Table S3. Solvent corrections (E_{solv}; benzene), SCF energy (E_{SCF}), dispersion energy ($E_{dispersion}$) and solvation-corrected electronic energy (E_{el}) for TPSSh-D4, and the sum of enthalpy and entropy corrections (G-E_{el}) for structures shown in **Figure 4a**. All energies are given in Hartree.

Structure	spin state	E_{solv}	E_{SCF}	E_{disp}	E_{el}	G-E_{el}	Final Gibbs
(dcype)FeMe$_2$, **Fe1**	S = 0	-0.01952983	-3044.712052	-0.1633682	-3044.89495	0.70017618	-3044.194774
	S = 1	-0.01978947	-3044.740808	-0.160500301	-3044.921098	0.69799475	-3044.223103
	S = 2	-0.01983841	-3044.740406	-0.158208116	-3044.918453	0.69518016	-3044.223273
PhPMe$_2$	S = 0	-0.00457784	-652.8974924	-0.027897278	-652.9299676	0.12934391	-652.8006236
(dcype)(PMe$_2$Ph)FeMe$_2$, **Fe3**	S = 0	-0.02151687	-3697.629201	-0.225044132	-3697.875762	0.86122381	-3697.014538
	S = 1	-0.02146964	-3697.632787	-0.224095038	-3697.878352	0.85909744	-3697.019254
	S = 2	-0.02223023	-3697.623796	-0.214367093	-3697.860393	0.84969094	-3697.010702
PMe$_3$	S = 0	-0.00023861	-461.0854368	-0.011631264	-461.0973067	0.08149797	-461.0158087
(dcype)(PMe$_3$)FeMe$_2$, **Fe3–PMe$_3$**	S = 0	-0.0184898	-3505.822193	-0.199655756	-3506.040339	0.81046875	-3505.22987
	S = 1	-0.01847849	-3505.825658	-0.199000216	-3506.043137	0.80890194	-3505.234235
	S = 2	-0.01929355	-3505.814489	-0.190083073	-3506.023866	0.80015385	-3505.223712
PEt$_3$	S = 0	-0.00416143	-579.0690962	-0.023901578	-579.0971592	0.16025715	-578.9369021
(dcype)(PEt$_3$)FeMe$_2$, **Fe3–PEt$_3$**	S = 0	-0.00534123	-3623.795931	-0.220451382	-3624.021724	0.8922909	-3623.129433
	S = 1	-0.00574598	-3623.798366	-0.21913073	-3624.023243	0.89051011	-3623.132733
	S = 2	-0.00567376	-3623.790036	-0.208030255	-3624.003740	0.88225969	-3623.121480
P^{i}Pr$_3$	S = 0	-0.00538998	-697.048727	-0.041646816	-697.0957638	0.24022271	-696.855541
(dcype)(P^{i}Pr$_3$)FeMe$_2$, **Fe3–P^{i}Pr$_3$**	S = 0	-0.02166392	-3741.753331	-0.242168145	-3742.017164	0.97436374	-3741.042800
	S = 1	-0.02194736	-3741.761951	-0.239946426	-3742.023844	0.97378593	-3741.050059
	S = 2	-0.02245451	-3741.782318	-0.219075666	-3742.023848	0.96088165	-3741.062966

Table S4. Solvent corrections (E_{solv}; benzene), SCF energy (E_{SCF}), dispersion energy ($E_{dispersion}$) and solvation-corrected electronic energy (E_{el}) for TPSSh-D4, and the sum of enthalpy and entropy corrections (G-E_{el}) for structures shown in **Figure 4b**. All energies are given in Hartree.

Structure	spin state	E_{solv}	E_{SCF}	E_{disp}	E_{el}	G-E_{el}	Final Gibbs
κ^1-O-arene complex with *trans*-Me$_2$ **Int1**	S = 0	-0.02523724	-4200.631213	-0.297955831	-4200.954406	1.07548496	-4199.878921
	S = 1	-0.02462643	-4200.594457	-0.298892926	-4200.917977	1.07534342	-4199.842633
	S = 2	-0.02466662	-4200.538043	-0.298892926	-4200.861603	1.07534342	-4199.786260
Pivalophenone **1**	S = 0	-0.00839383	-503.037751	-0.036278136	-503.082423	0.1814595	-502.900963
σ-agostic complex with *trans*-Me$_2$, **Int2**	S = 0	-0.02383988	-3547.752537	-0.227118467	-3548.003496	0.91706401	-3547.086432
	S = 1	-0.02465039	-3547.765209	-0.222677165	-3548.012536	0.90873718	-3547.103799
	S = 2	-0.02480269	-3547.746355	-0.22211714	-3547.993275	0.90688575	-3547.086389
C–H cleavage transition state with *trans*-Me$_2$, **TS1**	S = 0	-0.02390631	-3547.737722	-0.224719077	-3547.986347	0.91142727	-3547.07492
	S = 1	-0.02404037	-3547.710558	-0.222441369	-3547.95704	0.9058275	-3547.051212
	S = 2	-0.02399111	-3547.667682	-0.222441369	-3547.914115	0.9058275	-3547.008287

6-coordinate cyclometallation complex with σ-CH_4, **Int3**	S = 0	-0.02331695	-3547.77839	-0.226197314	-3548.027904	0.90723478	-3547.12067
	S = 1	-0.02332076	-3547.783547	-0.224334914	-3548.031203	0.90494006	-3547.126263
	S = 2	-0.0237135	-3547.781751	-0.217618134	-3548.023083	0.89924767	-3547.123835
5-coordinate cyclometallation complex after CH_4 dissociation, **Int4**	S = 0	-0.02467979	-3507.242358	-0.217478453	-3507.484516	0.8657724	-3506.618744
	S = 1	-0.02468227	-3507.247593	-0.215404962	-3507.487681	0.86390663	-3506.623774
	S = 2	-0.02474377	-3507.244663	-0.209901847	-3507.479308	0.85910906	-3506.620199
C−C Reductive Coupling Transition State with *trans*-Me_2, **TS2**	S = 0	-0.02472078	-3507.203202	-0.214978067	-3507.442901	0.86647525	-3506.576425
	S = 1	-0.0245275	-3507.224841	-0.215104522	-3507.464473	0.86570978	-3506.598763
	S = 2	-0.02476011	-3507.210174	-0.209566874	-3507.444501	0.86079347	-3506.583707
(dcype)Fe(η^6-**1**), **Fe2**	S = 0	-0.02318962	-3467.96045	-0.211127212	-3468.194767	0.8471723	-3467.347594
	S = 1	-0.02396283	-3467.946016	-0.206571066	-3468.17655	0.84130515	-3467.335245
	S = 2	-0.02497263	-3467.914157	-0.204491903	-3468.143622	0.83857377	-3467.305048
o-methyl pivalophenone, **2**	S = 0	-0.00879899	-542.3676826	-0.041835831	-542.4183174	0.20689826	-542.2114191

Table S5. Solvent corrections (E_{solv}; benzene), SCF energy (E_{SCF}), dispersion energy ($E_{dispersion}$) and solvation-corrected electronic energy (E_{el}) for TPSSh-D4, and the sum of enthalpy and entropy corrections (G-E_{el}) for structures shown in **Figure S36**. All energies are given in Hartree.

Structure	spin state	E_{solv}	E_{SCF}	E_{disp}	E_{el}	G-E_{el}	Final Gibbs
C-C reductive coupling transition state **TS_C_2H_6**	S = 0	-0.01959815	-3044.659922	-0.16298278	-3044.8425	0.69958608	-3044.14292
	S = 1	-0.02013902	-3044.693793	-0.15861012	-3044.87254	0.69754033	-3044.175
	S = 2	-0.02056934	-3044.652616	-0.155082494	-3044.828270	0.69579858	-3044.132470
σ-agostic complex with *cis*-Me_2, ***cis*-Int2AB**	S = 0	-0.02303637	-3547.742886	-0.231536232	-3547.997459	0.91552345	-3547.081936
	S = 1	-0.02294289	-3547.7353	-0.230713499	-3547.988956	0.91151175	-3547.077444
	S = 2	-0.02399294	-3547.747749	-0.223969751	-3547.995711	0.90591471	-3547.089797
C–H cleavage transition state with *cis*-Me_2, ***cis*-TS1A**	S = 0	-0.02282774	-3547.728361	-0.23280175	-3547.983991	0.91167734	-3547.072313
	S = 1	-0.02289105	-3547.701449	-0.231278604	-3547.955619	0.9073285	-3547.04829
	S = 2	-0.02400932	-3547.678327	-0.224906204	-3547.927242	0.89905296	-3547.028189
C–H cleavage transition state with *cis*-Me_2, ***cis*-TS1B**	S = 0	-0.02314311	-3547.732796	-0.231426144	-3547.987365	0.91135024	-3547.076015
	S = 1	-0.02280764	-3547.716701	-0.230781496	-3547.97029	0.90890237	-3547.061388
	S = 2	-0.02317851	-3547.691471	-0.227965365	-3547.942615	0.8998554	-3547.042759
6-coordinate cyclometallation complex with σ-CH_4, ***cis*-Int3A**	S = 0	-0.02285245	-3547.765675	-0.23021748	-3548.018745	0.91753086	-3547.101214
	S = 1	-0.02359276	-3547.783645	-0.225846806	-3548.033085	0.90614398	-3547.126941
	S = 2	-0.0234146	-3547.758309	-0.222720263	-3548.004444	0.9016964	-3547.102747
6-coordinate cyclometallation complex with σ-CH_4, ***cis*-Int3B**	S = 0	-0.02253351	-3547.775447	-0.230901703	-3548.028882	0.91069535	-3547.118187
	S = 1	-0.02273163	-3547.787574	-0.228423724	-3548.038729	0.90636461	-3547.132364
	S = 2	-0.02302598	-3547.765180	-0.225325417	-3548.013531	0.90224233	-3547.111289
5-coordinate cyclometallation complex after CH_4 dissociation, ***cis*-Int4A**	S = 0	-0.02403636	-3507.235292	-0.219755332	-3507.479084	0.86749502	-3506.611589
	S = 1	-0.02443504	-3507.249104	-0.216175605	-3507.489715	0.86411175	-3506.625603
	S = 2	-0.0238764	-3507.22555	-0.218331392	-3507.467758	0.86115001	-3506.606608
5-coordinate cyclometallation complex after CH_4 dissociation, ***cis*-Int4B**	S = 0	-0.02381398	-3507.241104	-0.221906408	-3507.486824	0.86858426	-3506.61824
	S = 1	-0.02372733	-3507.251552	-0.219960364	-3507.49524	0.86602661	-3506.629213
	S = 2	-0.0240298	-3507.228806	-0.21699273	-3507.469829	0.86205476	-3506.607774
C−C Reductive Coupling Transition State with *cis*-Me_2, ***cis*-TS2A**	S = 0	-0.02342101	-3507.207208	-0.224630989	-3507.45526	0.86777696	-3506.587483
	S = 1	-0.02384585	-3507.222161	-0.219387756	-3507.465395	0.86391739	-3506.601478
	S = 2	-0.02440696	-3507.195495	-0.216283832	-3507.436186	0.86072583	-3506.57546
C−C Reductive Coupling Transition State with *cis*-Me_2, ***cis*-TS2B**	S = 0	-0.02326372	-3507.205881	-0.225471083	-3507.454616	0.8672625	-3506.587353
	S = 1	-0.02384585	-3507.222161	-0.219387756	-3507.465395	0.86391739	-3506.601478
	S = 2	-0.02480449	-3507.202306	-0.21539378	-3507.442504	0.86215722	-3506.580347

Table S6. Solvent corrections (E_{solv}; benzene), SCF energy (E_{SCF}), dispersion energy ($E_{dispersion}$) and solvation-corrected electronic energy (E_{el}) for TPSSh-D4, and the sum of enthalpy and entropy corrections (G-E_{el}) for structures shown in **Figure S37**. All energies are given in Hartree.

Structure	spin state	E_{solv}	E_{SCF}	E_{disp}	E_{el}	G-E_{el}	Final Gibbs
σ-agostic complex with *cis*-Me_2, ***cis*-Int2C**	S = 0	-0.02343273	-3547.748032	-0.23056704	-3548.002032	0.9173296	-3547.084702
	S = 1	-0.023099	-3547.728201	-0.230120279	-3547.981421	0.91893751	-3547.062483
	S = 2	-0.0238563	-3547.745053	-0.224961534	-3547.993871	0.90695315	-3547.086918
C–H cleavage transition state with *cis*-Me_2, ***cis*-TS1C**	S = 0	-0.02298139	-3547.730839	-0.232971988	-3547.986792	0.91454458	-3547.072248
	S = 1	-0.02285179	-3547.706658	-0.232666388	-3547.962176	0.91016597	-3547.05201
6-coordinate cyclometallation complex with σ-CH_4, ***cis*-Int3C**	S = 0	-0.02278357	-3547.762544	-0.239236985	-3548.024565	0.91559542	-3547.108969
	S = 1	-0.02289975	-3547.774927	-0.230282163	-3548.028109	0.90681571	-3547.121294
	S = 2	-0.02351751	-3547.774580	-0.21808286	-3548.016180	0.89821821	-3547.117962
5-coordinate cyclometallation complex after CH_4 dissociation, ***cis*-Int4C**	S = 0	-0.02366611	-3507.231668	-0.225285157	-3507.480619	0.87005541	-3506.610564
	S = 1	-0.0236925	-3507.238154	-0.222870751	-3507.484717	0.86660532	-3506.618112
	S = 2	-0.02454657	-3507.23737	-0.212662694	-3507.474579	0.85853403	-3506.616045
C−C Reductive Coupling Transition State with *cis*-Me_2, ***cis*-TS2C**	S = 0	-0.02355032	-3507.21687	-0.224249997	-3507.46467	0.86901879	-3506.595651
	S = 1	-0.02422143	-3507.219756	-0.219122465	-3507.4631	0.86534576	-3506.597754
	S = 2	-0.02469818	-3507.204139	-0.21244679	-3507.441284	0.86105302	-3506.580231

Table S7. Solvent corrections (E_{solv}; benzene), SCF energy (E_{SCF}), dispersion energy ($E_{dispersion}$) and solvation-corrected electronic energy (E_{el}) for TPSSh-D4, and the sum of enthalpy and entropy corrections (G-E_{el}) for structures related to minimum energy crossing point (MECP) of the spin crossover from complex **Fe1** to **Fe3** during $PhPMe_2$ coordination. All energies are given in Hartree.

MECP	spin state	E_{solv}	E_{SCF}	E_{disp}	E_{el}	G-E_{el}	Final Gibbs
From *S* = 0 to *S* = 1	S = 0	-0.02145769	-3697.619742	-0.2241068	-3697.865306	0.85957096	-3697.005735
	S = 1	-0.02146537	-3697.632647	-0.2241068	-3697.87822	0.85957096	-3697.018649
From *S* = 1 to *S* = 0	S = 2	-0.02145277	-3697.619659	-0.224149283	-3697.865261	0.85957096	-3697.005690
	S = 0	-0.02146065	-3697.63259	-0.224149283	-3697.8782	0.85957096	-3697.018629
From *S* = 0 to *S* = 2	S = 1	-0.02227416	-3697.591837	-0.215854771	-3697.829966	0.85676169	-3696.973204
	S = 2	-0.02215823	-3697.621747	-0.215854771	-3697.85976	0.85676169	-3697.002999
From *S* = 2 to *S* = 0	S = 0	-0.02228579	-3697.592762	-0.215416008	-3697.830464	0.85383813	-3696.976626
	S = 1	-0.02216666	-3697.621789	-0.215416008	-3697.859372	0.85383813	-3697.005534
From *S* = 1 to *S* = 2	S = 2	-0.02233161	-3697.614263	-0.215089653	-3697.851684	0.85502151	-3696.996663
	S = 0	-0.02221633	-3697.623528	-0.215089653	-3697.860834	0.85502151	-3697.005813
From *S* = 2 to *S* = 1	S = 1	-0.02233843	-3697.614096	-0.214620368	-3697.851055	0.85332872	-3696.997726
	S = 2	-0.02221456	-3697.623373	-0.214620368	-3697.860208	0.85332872	-3697.006879

IX. References

1. Pangborn, A. B.; Giardello, M. A.; Grubbs, R. H.; Rosen, R. K.; Timmers, F. J. Safe and Convenient Procedure for Solvent Purification. *Organometallics* **1996**, *15*, 1518–1520.

2. Inoue, R.; Yamaguchi, M.; Murakami, Y.; Okano, K.; Mori, A. Revisiting of Benzophenone Ketyl Still: Use of a Sodium Dispersion for the Preparation of Anhydrous Solvents. *ACS Omega* **2018**, *3*, 12703–12706.

3. Zhang, T.; Whitehurst, W. G.; Pecoraro, M. V.; Kim, J.; Koenig, S. G.; Chirik, P. J. Redox-Neutral, Iron-Mediated Directed C–H Activation: General Principles and Mechanistic Insights. *J. Am. Chem. Soc.* **2024**, *146*, 30637–30652.

4. Casitas, A.; Krause, H.; Goddard, R.; Fürstner, A. Elementary Steps of Iron Catalysis: Exploring the Links between Iron Alkyl and Iron Olefin Complexes for Their Relevance in C–H Activation and C-C Bond Formation. *Angew. Chem., Int. Ed.* **2015**, *54*, 1521–1526.

5. Gabor, B.; Holle, S.; Jolly, P. W.; Mynott, R. The Preparation of Bis(η^3-Allyl)Iron(II) Complexes and Their Reactions with 1,3-Dienes. *J. Organomet. Chem.* **1994**, *466*, 201–209.

6. Evans, D. J.; Hitchcock, P. B.; Leigh, G. J.; Nicholson, B. K.; Niedwieski, A. C.; Nunes, F. S.; Soares, J. F. The Synthesis of Triangulo-Trimetal Complexes Containing Both Iron(II) and Vanadium(II). *Inorg. Chim. Acta* **2001**, *319*, 147–158.

7. Davies, S. G.; Felkin, H.; Watts, O. Hydride Reduction of the Cation [(η^5-C_5H_5)Fe(Triphos)]PF_6: Direct Nucleophilic Attack on the Metal, and Hydrogen Exchange in the Product Hydride (η^5-C_5H_5)FeH(Triphos)(Triphos = $Ph_2PCH_2CH_2PPhCH_2CH_2PPh_2$). *J. Chem. Soc., Chem. Commun.* **1980**, No. 4, 159–160.

8. Fulmer, G. R.; Miller, A. J. M.; Sherden, N. H.; Gottlieb, H. E.; Nudelman, A.; Stoltz, B. M.; Bercaw, J. E.; Goldberg, K. I. NMR Chemical Shifts of Trace Impurities: Common Laboratory Solvents, Organics, and Gases in Deuterated Solvents Relevant to the Organometallic Chemist. *Organometallics* **2010**, *29*, 2176–2179.

9. Neese, F. The ORCA program system. *WIREs Comput. Mol. Sci.* **2012**, *2*, 73–78.

10. Neese, F.; Wennmohs, F.; Becker, U.; Riplinger, C. The ORCA Quantum Chemistry Program Package. *J. Chem. Phys.* **2020**, *152*, 224108.

11. Neese, F. Software Update: The ORCA Program System, Version 4.0. *WIREs Comput. Mol. Sci.* **2018**, *8*, e1327.

12. Grimme, S.; Antony, J.; Ehrlich, S.; Krieg, H. A Consistent and Accurate Ab Initio Parametrization of Density Functional Dispersion Correction (DFT-D) for the 94 Elements H-Pu. *J. Chem. Phys.* **2010**, *132*, 154104.

13. Grimme, S.; Ehrlich, S.; Goerigk, L. Effect of the Damping Function in Dispersion Corrected Density Functional Theory. *J. Comput. Chem.* **2011**, *32*, 1456–1465.

14. Schäfer, A.; Huber, C.; Ahlrichs, R. Fully Optimized Contracted Gaussian Basis Sets of Triple Zeta Valence Quality for Atoms Li to Kr. *J. Chem. Phys.* **1994**, *100*, 5829–5835.

15. Eichkorn, K.; Weigend, F.; Treutler, O.; Ahlrichs, R. Auxiliary Basis Sets for Main Row Atoms and Transition Metals and Their Use to Approximate Coulomb Potentials. *Theor. Chem. Acc.* **1997**, *97*, 119–124.

16. Eichkorn, K.; Treutler, O.; Öhm, H.; Häser, M.; Ahlrichs, R. Auxiliary Basis Sets to Approximate Coulomb Potentials. *Chem. Phys. Lett.* **1995**, *240*, 283–290.

17. Eichkorn, K.; Treutler, O.; Öhm, H.; Häser, M.; Ahlrichs, R. Auxiliary Basis Sets to Approximate Coulomb Potentials (Chem. Phys. Letters 240 (1995) 283-290). *Chem. Phys. Lett.* **1995**, *242*, 652–660.

18. Kossmann, S.; Neese, F. Comparison of Two Efficient Approximate Hartee–Fock Approaches. *Chem. Phys. Lett.* **2009**, *481*, 240–243.

19. Neese, F.; Wennmohs, F.; Hansen, A.; Becker, U. Efficient, Approximate and Parallel Hartree–Fock and Hybrid DFT Calculations. A 'Chain-of-Spheres' Algorithm for the Hartree–Fock Exchange. *Chem. Phys.* **2009**, *356*, 98–109.

20. Neese, F. An Improvement of the Resolution of the Identity Approximation for the Formation of the Coulomb Matrix. *J. Comput. Chem.* **2003**, *24*, 1740–1747.

21. Ishida, K.; Morokuma, K.; Komornicki, A. The Intrinsic Reaction Coordinate. An Ab Initio Calculation for HNC→HCN and H^-+CH_4 → CH_4 + H^-. *J. Chem. Phys.* **1977**, *66*, 2153–2156.

22. Rolfes, J. D.; Neese, F.; Pantazis, D. A. All-Electron Scalar Relativistic Basis Sets for the Elements Rb–Xe. *J. Comput. Chem.* **2020**, *41*, 1842–1849.

23. Marenich, A. V.; Cramer, C. J.; Truhlar, D. G. Universal Solvation Model Based on Solute Electron Density and on a Continuum Model of the Solvent Defined by the Bulk Dielectric Constant and Atomic Surface Tensions. *J. Phys. Chem. B* **2009**, *113*, 6378–6396.

24. Harvey, J. N.; Aschi, M.; Schwarz, H.; Koch, W. The Singlet and Triplet States of Phenyl Cation. A Hybrid Approach for Locating Minimum Energy Crossing Points between Non-Interacting Potential Energy Surfaces. *Theor. Chem. Acc.* **1998**, *99*, 95–99.

25. Pettersen, E. F.; Goddard, T. D.; Huang, C. C.; Couch, G. S.; Greenblatt, D. M.; Meng, E. C.; Ferrin, T. E. UCSF Chimera—A Visualization System for Exploratory Research and Analysis. *J. Comput. Chem.* **2004**, *25*, 1605–1612.

26. Echemendía, R.; de Oliveira, K. T.; Burtoloso, A. C. B. Visible-Light-Promoted Synthesis of 1,3-Dicarbonyl Sulfoxonium Ylides. *Org. Lett.* **2022**, *24*, 6386–6390.

27. Dehmlow, E. V.; Kaiser, M.; Bollhöfer, J. Influence of Phase Transfer Catalyst Structure on the E:Z Ratio in the O-Alkylation of an Enolate. *New J. Chem.* **2001**, *25*, 588–590.

28. Christoffers, J. Facile nBu_4NF Mediated Benzylation Of Congested 1,3-Diketones. *Synth. Commun.* **1999**, *29*, 117–122.

29. Gilbert, H. F. “Rule of Thumb” for Deriving Steady State Rate Equations. *J. Chem. Educ.* **1977**, *54*, 492.

30. Nielsen, C. D.-T.; Burés, J. Visual Kinetic Analysis. *Chem. Sci.* **2019**, *10*, 348–353.

31. Burés, J. Variable Time Normalization Analysis: General Graphical Elucidation of Reaction Orders from Concentration Profiles. *Angew. Chem., Int. Ed.* **2016**, *55*, 16084–16087.

32. Burés, J. A Simple Graphical Method to Determine the Order in Catalyst. *Angew. Chem., Int. Ed.* **2016**, *55*, 2028–2031.
